# Fanconi Anemia Pathway Genes Advance Cervical Cancer *via* Immune Regulation and Cell Adhesion

**DOI:** 10.3389/fcell.2021.734794

**Published:** 2021-11-15

**Authors:** Shizhi Wang, Bo Ding, Mengjing Cui, Wenjing Yan, Qianqian Xia, Dan Meng, Siyuan Shen, Shuqian Xie, Hua Jin, Xing Zhang

**Affiliations:** ^1^ Key Laboratory of Environmental Medicine Engineering, Ministry of Education, School of Public Health, Southeast University, Nanjing, China; ^2^ Department of Gynecology and Obstetrics, Zhongda Hospital, School of Medicine, Southeast University, Nanjing, China; ^3^ Clinical Laboratory, Affiliated Tumor Hospital of Nantong University (Nantong Tumor Hospital), Nantong, China

**Keywords:** fanconi anemia pathway, cervical carcinoma, prognosis, immune, cell adhesion, machine learning

## Abstract

Fanconi anemia (FA) pathway is a typical and multienzyme-regulated DNA damage repairer that influences the occurrence and development of disease including cancers. Few comprehensive analyses were reported about the role of FA-related genes (FARGs) and their prognostic values in cancers. In this study, a comprehensive pan-cancer analysis on 79 FARGs was performed. According to the correlation analyses between HPV integration sites and FARGs, we found that FARGs played specific and critical roles in HPV-related cancers, especially in cervical cancer (CC). Based on this, a FARGs-associated prognostic risk score (FPS) model was constructed, and subsequently a nomogram model containing the FPS was developed with a good accuracy for CC overall survival (OS) and recurrence-free survival (RFS) outcome prediction. We also used the similar expression pattern of FARGs by consensus clustering analysis to separate the patients into three subgroups that exhibited significant differential OS but not RFS. Moreover, differential expressed genes (DEGs) between the two risk groups or three clusters were identified and immune pathways as well as cell adhesion processes were determined by functional enrichment analysis. Results indicated that FARGs might promote occurrence and development of CC by regulating the immune cells’ infiltration and cell adhesion. In addition, through the machine learning models containing decision tree, random forest, naïve bayes, and support vector machine models, screening of important variables on CC prognosis, we finally determined that *ZBTB32* and *CENPS* were the main elements affecting CC OS, while *PALB2* and *BRCA2* were for RFS. Kaplan-Meier analysis revealed that bivariate prediction of CC outcome was reliable. Our study systematically analyzed the prognostic prediction values of FARGs and demonstrated their potential mechanism in CC aggressiveness. Results provided perspective in FA pathway-associated modification and theoretical basis for CC clinical treatments.

## Introduction

Fanconi anemia (FA) is an uncommon congenital autosomal recessive disease with clinical characteristics that include progressive bone marrow failure and cancer predisposition (Kee and D'Andrea, 2010; Kim and D'Andrea, 2012). Patients suffering from FA generally develop pancreatitis rapidly over a short time, and death often occurs because of complications. Studies have shown that the potential genetic defect causing FA can occur in any of the 16 FANC-genes, often resulting in abnormal DNA damage repair pathway (Park et al., 2016; Wang et al., 2020). Increasing direct evidence has indicated the clinical features of FA involve the development of a series of important organ systems, including the heart, kidneys, and gastrointestinal tract (Auerbach, 1995; D'Andrea and Grompe, 2003; Garcia and Benitez, 2008). This indicates that FA-related genes (FARGs) are essential for human development.

The FA pathway acts as a DNA damage repair pathway and crosstalk with the ATM and ATR pathways in cell-cycle control (Spriggs and Laimins, 2017). FARGs collaboratively repair DNA interstrand cross-links (ICLs), a side effect of the DNA replication process with high cellular cytotoxicity, thereby effectively preventing the occurrence and development of many disorders, including cancers (D'Andrea and Grompe, 2003; Shakeel et al., 2019). During ICLs repair, a series of FARGs is activated successively and play their respective parts in different stages according to their functions. FARGs work together to rescue unexpected DNA mutations in a timely manner, which is essential for maintaining good health. In addition to the important role of the FA pathway in DNA replication, increasing evidence has shown that this pathway is inseparable from the occurrence and development of a variety of cancers. One study reported that the FA pathway is associated with upregulated genes in small-cell lung cancer, revealing a potential function of DNA replication in cancer development (Misono et al., 2021). Madubata et al. pointed out that FARGs function in bladder carcinoma genetic predisposition due to their frequent somatic variants and mentioned the feasibility of directed DNA damage repair therapy in bladder carcinomas (Madubata et al., 2017). Interestingly, the inactivation of FA DNA repair leads to genomic instability in patients and increases the risk of HPV-associated disorders such as cervical cancer (CC) and head and neck cancer (Spriggs and Laimins, 2017). Recent results have shown that during HPV16 oncogene E7 caused HPV-related tumors, abnormality in FARGs can significantly accelerate the disease, and can even lose the need for continuous E7 expression in the procedure (Park et al., 2016). The absence of FARGs greatly enhances the success rate of HPV viral DNA integration into the human genome by decreasing genome stability (Spardy et al., 2007). Accordingly, abnormal FARGs are a devastating focus for diseases, especially HPV-associated cancers.

Although research continues to elucidate the specific molecular mechanism of FARGs in pathogenesis, few studies have comprehensively analyzed the role of FARGs in cancers, and there is no analysis to predict tumor prognosis using FARGs. In this study, we performed pan-cancer analyses and identified important elements of FARGs in CC prognosis prediction. From molecular mechanism analysis, our results indicated that FARGs can protect against adverse prognosis by participating in the regulation of immune processes and focal adhesion pathways in CC. These findings provide distinctive insight on the role of FARGs and reveal a sensitive biomarker for CC treatment.

## Materials and Methods

### Study Design and Data Collection

**FIGURE 1:**
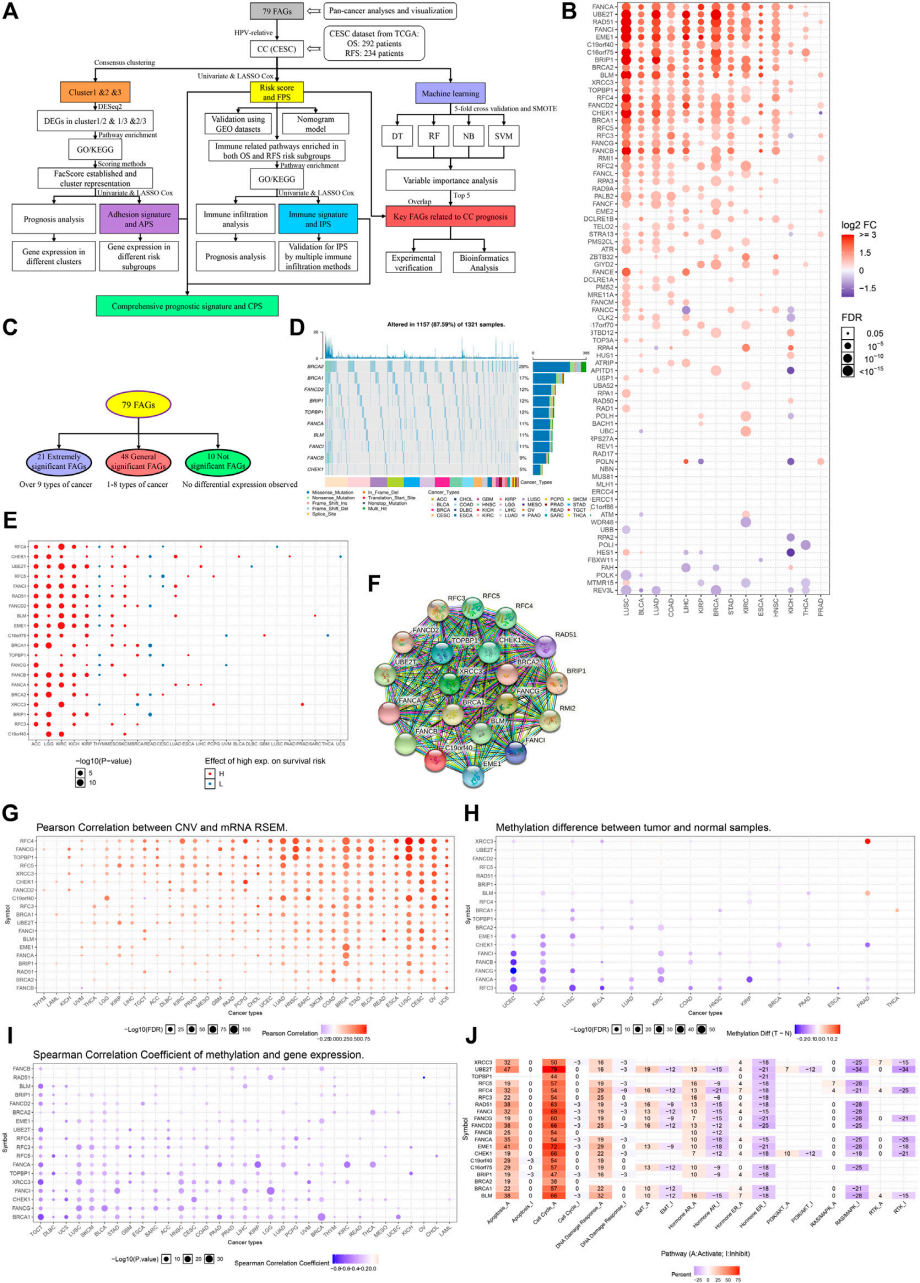
Pan-cancer analyses of FARGs among tumors. **(A)** The flowchart of the whole study. FARGs, Fanconi anemia related genes; CESC, cervical squamous cell carcinoma and endocervical adenocarcinoma; DEGs, differentially expressed genes; GO, gene ontology; KEGG, Kyoto Encyclopedia of Genes and Genomes; OS, overall survival; RFS, recurrence-free survival; LASSO, least absolute shrinkage and selection operator algorithm; TCGA, The Cancer Genome Atlas; GEO, gene expression omnibus; SMOTE, the synthetic minority over-sampling technique; DT, decision tree; RF, random forest; NB, naïve bayes; SVM, support vector machines. **(B)** Different RNA expression patterns of FARGs between tumor and normal tissues among 14 cancers. ACC, adrenocortical carcinoma; BLCA, bladder urothelial carcinoma; BRCA, breast invasive carcinoma; CESC, cervical squamous cell carcinoma and endocervical adenocarcinoma; CHOL, cholangiocarcinoma; COAD, colon adenocarcinoma; DLBC, lymphoid neoplasm diffuse large B-cell lymphoma; ESCA, esophageal carcinoma; GBM, glioblastoma multiforme; HNSC, head and neck squamous cell carcinoma; KICH, kidney chromophobe; KIRC, kidney renal clear cell carcinoma; KIRP, kidney renal papillary cell carcinoma; LAML, acute myeloid leukemia; LGG, brain lower grade glioma; LIHC, liver hepatocellular carcinoma; LUAD, lung adenocarcinoma; LUSC, lung squamous cell carcinoma; MESO, mesothelioma; OV, ovarian serous cystadenocarcinoma; PAAD, pancreatic adenocarcinoma; PCPG, pheochromocytoma and paraganglioma; PRAD, prostate adenocarcinoma; READ, colon adenocarcinoma/rectum adenocarcinoma esophageal carcinoma; SARC, sarcoma; SKCM, skin cutaneous melanoma; STAD, stomach adenocarcinoma; TGCT, testicular germ cell tumors; THCA, thyroid carcinoma; THYM, thymoma; UCEC, uterine corpus endometrial carcinoma; UCS, uterine carcinosarcoma; UVM, uveal melanoma. **(C)** Grouping methods of extremely significant FARGs. **(D)** The relation between extremely significant FARGs and overall survival among cancers. **(F)** The PPI network of extremely significant FARGs. **(E)** Type and frequency analysis of CNV mutations in extremely significant FARGs **(Top 10)**. **(G)** Pearson correlation between CNV and RNA expression. **(H)** Extremely significant FARGs methylation level comparison between cancers and normal samples. **(I)** Spearman correlation analyses of gene methylation and expression. **(J)** The essential pathway enrichment analyses of extremely significant FARGs by GSVA.

All the bioinformatics analyses mentioned in this article were performed as a flow diagram in Figure 1A. The data and its clinical feature information of cancers acquired and visualized from the Gene Set Cancer Analysis (GSCA, bioinfo.life.hust.edu.cn/web/GSCALite/). The detailed data of cervical squamous cell carcinoma (CESC) and other cancers which were used for validation were downloaded from The Cancer Genome Atlas (TCGA) database and standardized by R program. In addition, GSE3791 and GSE63514 were downloaded from the Gene Expression Omnibus (GEO) database. As for prognostic survival analysis of CESC overall survival (OS) and recurrence-free survival (RFS), a total of 292 samples and 234 samples with complete clinicopathological information, respectively.

The Fanconi anemia pathway related genes (FARGs) were obtained from GSEA (PID_FANCONI_PATHWAY, REACTOME_FANCONI_ANEMIA_PATHWAY, REACTOME_REGULATION_OF_THE_FANCONI_ANEMIANEMIA_PATHWAY), KEGG database (map03460) and PathCards (human biological pathway unification, https://pathcards.genecards.org/), forasmuch total of 79 FARGs were identified through the union operation.

### Visualization of Protein-Protein Interaction Network

Through differential expression analysis by GSCA, 79 FARGs were divided into 3 groups. We use STRING (https://www.string-db.org/) to construct and visualize the interaction network of the proteins in each group. In addition to STRING, GeneMANIA (http://genemania.org/) was also used to visualize the connection between proteins of interest.

### Identification of Different Expression Genes

To identify differentially expressed genes (DEGs) in HPV-positive groups (*n* = 286) and HPV-negative groups (*n* = 19), a comparison process was performed with CESC count data by DESeq2 package. The genes with adjusted *p*-values < 0.05 and |log2FoldChange (FC)| > 1 were filtered out as DEGs. Similar analysis procedures were also used for the subgroups with different risk ranks and with distinct clustering results mentioned in this paper. In addition, the intersection of common genes was realized and visualized by the online Venny 2.1 tool (https://bioinfogp.cnb.csic.es/tools/venny/index.html).

### Functional Enrichment Analysis

To explore the activity of cancer hallmark pathways, gene set variation analysis (GSVA) by “GSVA” and “GSEABase” packages was performed to discover potential FARGs functions in CESC. To identify the possible basic functions of *ZBTB32* in CESC, the gene set enrichment analysis (GSEA) by “clusterprofiler” packages was completed. We choose the “c2.cp.kegg.v7.3.symbols.gmt” and “c5.all.v7.3.symbols.gmt”, which were downloaded from online website GSEA, as the reference gene set files for both GSVA and GSEA analyses. Moreover, gene ontology (GO) and Kyoto Encyclopedia of Gens and Genomes (KEGG) pathway enrichment were performed by “clusterprofiler” packages based on DEGs.

### Consensus Clustering on the Basic of FA-Related Genes

To quest the potential function of FARGs in CC, patients from TCGA were separated into three clusters by consensus clustering analysis using “ConsensusClusterPlus” package. Then the principal component analysis (PCA) and t-distributed stochastic neighbor embedding (t-SNE) were utilized to reveal the expression patterns of genes in subgroups by “ggplot2” and “Rtsne” packages. In addition, the Kaplan-Meier analysis was used to assess the different OS and RFS time between subgroups, respectively. Moreover, DEGs were screened by “DESeq2” package among clusters.

### Generation of Cell Adhesion-Related Gene Signature

To qualify the cell adhesion-related patterns in CC, we constructed a scoring system to assess the cell adhesion model among patients, which was named as FacScore. The DEGs obtained from different FARGs clusters were normalized among all CC samples. Then the overlap genes were extracted for deeper analysis by adopting unsupervised clustering method. Next, the prognosis analysis for each overlap gene was calculated by univariate Cox regression and the essential elements were marked. The marked genes were used to PCA for the FacScore. Both the first and second principal component were selected to participate in FacScore calculation.

### Construction and Evaluation of Cervical Cancer Prognosis Risk Score Model

For the FARGs-based prognosis score (FPS) signature model, univariate Cox regression analyses were carried out to distinguish the independent prognostic predictors among FARGs by “survival” package for both OS and RFS. Then the prognostic models were performed by Least Absolute Shrinkage and Selection Operator (LASSO) algorithm by “glmnet” package and powerful predictors were filtered for CESC. According to these signatures, the FPS of LASSO models was calculated for prediction and subsequently divided patients into high-FPS and low-FPS groups based on the median score. Moreover, we use the HPV-related tumors in the GEO database and major gynecological tumors to validate the risk model.

For the immune genes-based prognosis score (IPS) signature model, screening criteria were used as follows: 1) be significantly expressed in high-FPS and low-FPS groups for both OS and RFS, respectively (|log2FC| > 1 and adjust *p*-value < 0.05); 2) overlaps with the immune genes acquired from ImmPort database; 3) choose the specific genes for OS and RFS separately. Next, the univariate Cox regression analyses were performed for input genes screening and the LASSO models based on the specific immune genes were built. According to these, the IPS was calculated for prediction and subsequently divided patients into high-IPS and low-IPS groups based on the median score.

For the cell adhesion-based prognosis score (APS) signature model, genes were extracted from cell adhesion-related terms of function enrichment analysis. Then these genes were treated by univariate Cox regression analyses and LASSO model construction. The APS was calculated for CC prognosis prediction and the high-APS and low-APS groups were divided.

For the comprehensive prognosis score (CPS) signature model, genes were integrated from FPS, IPS, and APS. The Kaplan-Meier survival curve was performed to compare the risks between groups. The accuracy of the model was evaluated by the receiver operating characteristic (ROC) analysis using the “timeROC” package.

To evaluate whether the risk score model could be a steady predictor for CC prognosis, univariate Cox regression and multivariate Cox regression analysis were performed to find the clinical characteristics which were vital to survival and identified the independent factors. Moreover, a nomogram with critical factors was constructed by the “rms” package. Subsequently, the calibration curve was formulated (for 2-years, 3-years, and 5-years) with a 45° line indicating the best prediction effect. Similarly, C-index was calculated of clinical features. In addition, decision curve analysis (DCA) with 2-, 3-, and 5-years was performed to measure whether the established model was suitable for clinical application by utilizing the “ggDCA” package. The *x*-axis of the DCA plot represented the risk threshold, and the *y*-axis represented net benefit.

### Immune Infiltration Analysis Among Different Groups

To further explore the immune infiltration of CC, we used Cell-type Identification By Estimating Relative Subsets Of RNA Transcripts (CIBERSORT, http://cibersort.stanford.edu/) to resolve the abundance of important immune cells (Newman et al., 2015) by R program and only the features with *p*-value < 0.05 were significant. In addition, other tools were also performed to calculate the level of immune infiltration in each patient, which included xcell (Aran et al., 2017), TIMER (Li et al., 2020), quanTIseq (Finotello et al., 2019), Microenvironment Cell Populations-counter (MCP-counter) (Becht et al., 2016), and EPIC (Yang et al., 2021). We aimed to analyze the correlation between IPS and immune infiltration level obtained by distinct methods, and therefore served as an important basis for IPS reliability.

### Identify Important Molecular Markers Through Machine Learning

To further accurately screen out the vital molecules that can affect the prognosis of CC from a series of FARGs, we used four machine learning methods to predict CC survival and arranged the variables according to their importance and visualized the results by the R program at the end. As four of the best classifiers, decision tree (DT), random forest (RF), support vector machines (SVM), and naïve bayes (NB), were performed to participate in the CC prognosis prediction for both OS and RFS.

After excluding unusable samples, the number of available OS patients in TCGA was 292 (219 alive and 71 dead), while the number of RFS was 234 (206 recurrence-free and 28 recurrence). These typical unbalanced data was the consequence of quantitative difference of cases with different survival outcomes. To solve the potential poor fitting effect caused by unbalanced data, the synthetic minority over-sampling technique (SMOTE), a method of interpolating between samples of a minority class to generate additional samples (Roberts, 1991; Fan et al., 2021), was adopted for data preprocessing by “DMwR” package of R.

A decision tree (DT) algorithm is usually a process of recursively filtering the optimal features, and segmenting the training data according to the characters, so that each sub-data set has the best classification process. In machine learning, random forest (RF) algorithm is a classifier that contains multiple decision trees. Sampling with replacement was taken from the original data set to construct a sub-data set. It can be evaluated internally, which means that an unbiased estimate of the error can be established during the generation process. In order to reduce out-of-bag (OBB) errors and save running time, we used 400 trees to build an RF model and arrange the variables in order of importance. DT was performed by the “rpart” package of R and RF was completed by the “randomForest” package.

The naïve Bayes (NB) method is another most commonly used classification algorithm in addition to decision trees. It is implemented based on Bayes’ theorem and the assumption of independence of feature conditions. Before fitting, we assumed that the particular feature in the class was independent of the existence of any other features, which means that each feature is independent of each other. NB mainly calculated the probability that the sample can be divided into each category based on the distribution of features in the new sample and output the category with the highest probability as the prediction result. The “klaR” package of R was used to achieve this step.

SVM is an algorithm that is widely used in two classifications and multiple classifications. In the prediction process of binary classification, the SVM core can describe edge lines from adjacent data points to divide sample groups. In that case, if the data are linearly inseparable, mathematical functions need to be used to transform the data to higher-attribute levels. The multi-dimensional space is carried out until the separability condition is met. The SVM model was performed by the “e1071” package of R.

### Clinical Specimens Collection and QPCR Detection

The authors pointed out that the Ethics Committee of Southeast University approved this study, and informed consent was acquired from each participant recruited and all samples were used in compliance with the institution’s ethical regulations. A total of 41 patients were admitted to Zhongda Hospital Affiliated with Southeast University including 19 CC tissues and 22 normal controls. All samples were stored in the ultra-low temperature freezer with RNAlater™ Stabilization Solution (AM7021, Thermo Fisher, United States) treated for RNA extraction by Trizol (Invitgen, United States) according to protocols and following qPCR using StepOne Plus (ABI, United States). Detailed patient data were uploaded to Supplementary Table S1 and primer sequences for qPCR were listed in Supplementary Table S2.

### Statistical Analyses

All the statistical analyses in this study were performed by R software (v4.0.2). Wilcox test was used to compare the genes expression level between tumor and normal tissues and one-way ANOVA was utilized to compare the different patterns among FARGs-related clusters. Kaplan-Meier survival curve with log-rank test was used to OS and RFS comparison between different groups by “survival” and “survminer” package of R. Spearman correlation analysis was performed to explore the correlation between genes expression. Chi-square test relating to CPS risk was carried out for each risk group. The visualization of the above results was mainly realized by R software and GraphPad Prism (v8.0.2). *p* < 0.05 was defined as a significant criterion in this study.

## Results

### Altered Expression of Fanconi Anemia Pathway-Related Genes and Pan-Cancer Analysis

Pan-cancer analysis based on 33 cancers from The Cancer Genome Atlas (TCGA) was performed to evaluate FARGs expression pattern, epigenetic information, and prognostic prediction values (Table 1). Visualization using the GSCALite platform revealed that genes exhibited varying expression patterns (Figure 1B). Genes were classified into three groups: “extremely significant FARGs (esFARGs)”, “general significant FARGs (gsFARGs)”, and “not significant FARGs (nsFARGs)”, according to the frequency of molecular differential expression (Figure 1C). In addition, *RPS27AP11* was significantly expressed in more than 10 cancer types (Supplementary Figure S1), so *RPS27AP11* was also in the first category. For esFARGs (Figure 1F), survival analysis revealed that most with high expression levels were associated with poor survival outcomes in ACC, LGG, KIRC, KICH, KIRP, MESO, and SKCM, and with good outcomes in THYM, READ, and CESC (Figure 1D). CNV analysis showed that BRCA2 (29%) and BRCA1 (17%) were the two most frequently mutated genes and missense mutations had the highest degree of occurrence (Figure 1E). Moreover, Pearson correlation indicated that most FARG expression levels were significantly associated with CNV frequently in cancers, particularly in LUSC, CESC, UCS, BRCA, and OV (Figure 1G). Furthermore, the FARG methylation level, epigenetic information with important reference meaning, was analyzed and visualized using GSCALite platform. As Figure 1H showed, *FANCI*, *-B*, *-G*, *-A*, and *RFC3* methylation levels were significantly downregulated in UCEC and LIHC while *XRCC3* methylation levels were upregulated in PRAD. Notably, when we focused on the association between methylation levels and gene expression, the results showed a broad negative association (Figure 1I). However, lower gene methylation levels can suggest cancer prognostic outcomes except for in 10 of 20 esFARGs for KIRC prediction (Supplementary Figure S1B). To further evaluate the molecular process and mechanism by which esFARGs are involved, GSVA analysis was performed and pointed that esFARGs could activate cell cycle as well as cell apoptosis and inhibit RAS/MAPK signaling pathway as well as hormone ER (Figure 1J). We also attempted to identify weaknesses in the gene list by conducting a drug sensitivity analysis based on GDSC and CTRP. In general, low expression of *FANCD2*, *BLM*, *BRCA1*, *TOPBP1*, and *CHEK1* was significantly associated with drug resistance in the patient. High expression of genes was associated with patient resistance to a few drugs, including Trametinib, Selumetinib, RDEA119, and PD-0325901 (Supplementary Figures S1C,D).

**TABLE 1:** FARGs and its location on the chromosome.

Gene	Chr	Start	End
APITD1/CENPS/FAAP16	1	10430443	10442809
ATM	11	108222484	108369102
ATR	3	142449235	142578826
ATRIP	3	48446710	48467645
BACH1	21	29194071	29630751
BLM	15	90717327	90816165
BRCA1	17	43044295	43170245
BRCA2	13	32315474	32400266
BRIP1	17	61681266	61863521
CHEK1	11	125625136	125676255
CLK2	1	155262868	155278491
DCLRE1A	10	113834725	113854383
DCLRE1B	1	113905141	113914086
EME1	17	50373220	50381483
EME2	16	1773207	1781708
ERCC1	19	45407333	45478828
ERCC4	16	13920157	13952345
FAAP100/C17orf70	17	81539885	81553961
FAAP20/C1orf86	1	2184461	2212720
FAAP24/C19orf40	19	32972209	32978222
FAH	15	80152490	80186946
FAN1/MTMR15	15	30903852	30943108
FANCA	16	89737549	89816657
FANCB	X	14843407	14873069
FANCC	9	95099054	95317709
FANCD2	3	10026414	10101930
FANCE	6	35452361	35467103
FANCF	11	22622519	22626787
FANCG	9	35073835	35080016
FANCI	15	89243949	89317261
FANCL	2	58159243	58241372
FANCM	14	45135940	45200890
FBXW11	5	171861549	172006873
HES1	3	194136145	194138732
HUS1	7	47695730	47979581
MLH1	3	36993332	37050918
MRE11A	11	94415578	94493908
MUS81/SLX3	11	65857126	65867653
NBN	8	89933336	90003228
PALB2	16	23603160	23641310
PMS2/PMS2CL	7	5973239	6009125
POLH	6	43576150	43615660
POLI	18	54269404	54321266
POLK	5	75511756	75601144
POLN	4	2071918	2242121
RAD1	5	34905264	34918989
RAD17	5	69369293	69414801
RAD50	5	132556019	132646344
RAD51	15	40694774	40732339
RAD9A	11	67317871	67398410
REV1	2	99400475	99490035
REV3L	6	111299028	111483715
RFC2	7	74231499	74254458
RFC3	13	33818049	33966558
RFC4	3	186789880	186807058
RFC5	12	118013588	118033130
RMI1	9	83980711	84004074
RMI2/C16orf75	16	11249619	11351762
RPA1	17	1829702	1900082
RPA2	1	27891524	27914746
RPA3	7	7636518	7718607
RPA4	X	96883908	96885467
RPS27A	2	55231903	55235853
RPS27AP11	6	113581463	113581999
SLX1A/GIYD1/SLX1	16	30193887	30197561
SLX1B/GIYD2	16	29454501	29458219
SLX4/FANCP/BTBD12	16	3581181	3611598
STRA13/CENPX/FAAP10	17	82018702	82024107
TELO2	16	1493344	1510457
TOP3A	17	18271428	18315007
TOPBP1	3	133598175	133661893
UBA52	19	18571730	18577550
UBB	17	16380798	16382745
UBC	12	124911604	124917368
UBE2T	1	202331657	202341980
USP1	1	62436297	62451804
WDR48	3	39051998	39096671
XRCC3	14	103697609	103715504
ZBTB32	19	35704527	35717038

For the gsFARGs, a PPI network of tightly linked proteins similar to esFARGs was constructed (Supplementary Figures S2A,B). In contrast to esFARGs, it was difficult to find coincident expression patterns in gsFARGs that had potential prognostic effect in a certain cancer (Supplementary Figure S2C). In KIRC, the high expressions of *RPA4*, *RAD9A*, *FANCL*, *FAAP100*, *TELO2,* and *CLK2* were associated with poor outcome, while *FANCC*, *RAD50*, and *FBXW11* showed the opposite effect. CNV analysis results showed that *ATM* (22%) was the most frequently mutated gene among cancers (Supplementary Figure S2D) and a result similar to that of esFARGs demonstrated a close connection between CNV and mRNA, especially in BRCA (Supplementary Figure S2E). *FANCE* methylation levels in different cancers showed the opposite trend, upregulated in KIRP and downregulated in LUSC, while *FANCC* methylation was upregulated in THCA and downregulated in UCEC (Supplementary Figure S2F). Possibly, regarding this phenomenon, the same gene has opposite prognostic functions in different cancers (Supplementary Figure S2G); however, gene methylation was inversely correlated with expression in most cases, expect for *RAD1*, *ATR,* and *ATM* (Supplementary Figure S2H). Pathway enrichment analysis demonstrated that gsFARGs could activate cell cycle and DNA damage response (Supplementary Figure S2I). Drug sensitivity analysis indicated that patient resistance to trametinib, RDEA119, and selumetinib related to high expression of *FANCC*, *USP1*, and *RMI1*, while resistance to FK866, PIK-93, NPK76-II-72-1, and navitociax showed the opposite mechanism (Supplementary Figures S2J,K).

When nsFARGs were focused, *ERCC1*, *ERCC4,* and *MUS81* were key elements in the PPI network, with extensive links formed with each member (Supplementary Figures S3A,B). Although more than half of the genes in KIRC, ACC, and BRCA were significant prognostic indicators, the direction of association suggested that complex cancer-specific mechanisms were involved, distinct from the common mechanisms in esFARGs (Supplementary Figure S3C). Four genes, *REV1* (25%), *ERCC4* (24%), *MLH1* (23%), and *NBN* (20%) were mutated more than 20% of the time in patients and missense mutation still had the highest degree of occurrence (Supplementary Figure S3D). CNV frequency was significantly correlated with RNA expression (Supplementary Figure S3E). Our results did not indicate broad differences in the level of methylation in cancer, and the observed association between methylation and prognosis was sparse (Supplementary Figures S3F,G). Only the results of correlation analysis between methylation level and gene expression were similar to those of two other groups (Supplementary Figure S3H). Pathway enrichment analysis showed that nsFARGs mainly activate cell cycle, the same as other FARG groups, yet few genes provided information on inhibiting pathways (Supplementary Figure S3I). As for drug sensitivity analysis, *RAD17* was associated with the reactivity of various drugs (Supplementary Figures S3J,K).

In brief, we found that FARGs are abnormal in a variety of cancers. The identification of such abnormalities and early pursuit of therapeutic measures are likely to be the key mechanism to solve the occurrence and development of some cancers.

### Establishment of FARG-Associated Prediction Score and Prognostic Model Based on the FARG Expression in Cervical Cancer

**FIGURE 2:**
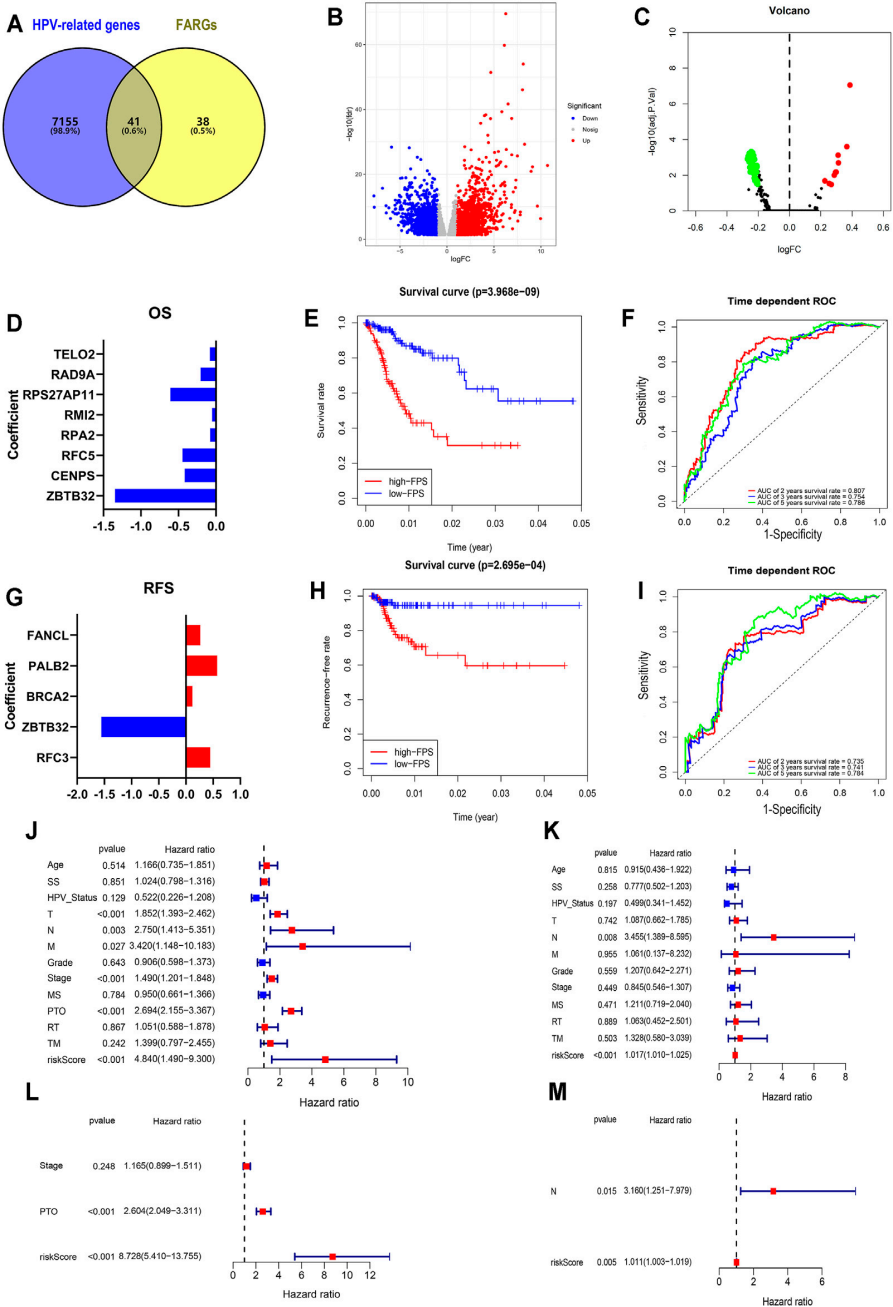
Establishment and validation of FPS in CC. **(A)** The overlap of HPV-related genes and FARGs. **(B)** Volcano plot of different genes in HPV+ (*n* = 286) and HPV− (*n* = 19) subgroups of TCGA CESC patients. **(C)** GSVA analyses of FARGs in CESC. **(D)** The filtered variables from OS-LASSO model and coefficients. **(E)** The Kaplan-Meier survival curves for CC patients with high and low FPS. **(F)** ROC curves described the predictive ability of OS-LASSO model for 2-, 3-, and 5-years survival probabilities. **(G)** The filtered variables from RFS-LASSO model and coefficients. **(H)** The KM analyses for CC patients with high and low FPS. **(I)** ROC curves described the predictive ability of RFS-LASSO model for 2-, 3-, and 5-years survival probabilities. **(J)** The correlation between clinicopathological parameters included FPS and OS using univariate Cox regression. **(K)** The correlation between clinicopathological parameters included FPS and RFS using univariate Cox regression. **(L)** The correlation between significant features and OS by multivariate Cox regression analysis. **(M)** The correlation between significant features and RFS by multivariate Cox regression analysis. SS, smoking status; MS, menopause status; PTO, primary therapy outcome; RT, radiation therapy; TM, target or molecular therapy.

Mentioned above, inactivation of DNA repair by FARGs will cause genomic instability in patients, which will lead to a higher success rate of HPV viral integration into the patient’s genome and increase the risk of developing HPV associated cancers such as CC (Spardy et al., 2007; Hoskins et al., 2008; Hoskins et al., 2009). We explored clues about the prognosis of CC, a cancer with persistent infection of HPV virus as its exact etiology, through FARGs. To evaluate the HPV-related effect of FARGs in CC, significant differences in the expression patterns between HPV+ and HPV- patients were investigated and over 7,000 differential expressed genes (DEGs) were identified; 41 of 79 FARGs involved (Figures 2A,B). A GSVA analysis based on FARGs was carried out to further explore the potential impact of FARGs on CC, and the result showed that different channels mainly concentrate on various “ABNORMAL” processes, which illustrated the indispensable role of FARGs in maintaining normal biochemical pathways in humans (Figure 2C and Supplementary Table S3). Furthermore, spearman correlation analysis was conducted between FARGs and 380 high-frequency genes (Hu et al., 2015), which are DNA breakpoints caused by HPV-DNA integration. As shown in Supplementary Figure S4A and Table 2, there was a surprisingly broad correlation between FARGs expressions and hot-spot gene expression, almost all of which were positive (|r| > 0.400, *p* < 0.050).

**TABLE 2:** Correlation between HPV integrated heat gene and FARGs (Top 50).

Hot spots	FAG	cor_R	*p*-value
ATR	TOPBP1	0.817	<0.001
BLM	FANCI	0.804	<0.001
KLHL28	FANCM	0.790	<0.001
DNAJC19	RFC4	0.707	<0.001
PALB2	TOPBP1	0.679	<0.001
ATP11B	TOPBP1	0.669	<0.001
PALB2	RMI1	0.661	<0.001
SLC25A36	TOPBP1	0.656	<0.001
BRCA1	BRIP1	0.651	<0.001
PDIK1L	RPA2	0.621	<0.001
BLM	RAD51	0.615	<0.001
BRCA1	BRCA2	0.612	<0.001
PALB2	FANCM	0.605	<0.001
BRCA1	FANCI	0.597	<0.001
ATR	RMI1	0.597	<0.001
CCR7	ZBTB32	0.587	<0.001
ATR	PALB2	0.587	<0.001
PALB2	BRIP1	0.582	<0.001
DEK	TOPBP1	0.580	<0.001
PTPN13	PALB2	0.579	<0.001
KLHL28	PALB2	0.578	<0.001
IREB2	BLM	0.577	<0.001
TBL1XR1	RFC4	0.576	<0.001
ATP11B	PALB2	0.575	<0.001
BRCA1	RFC5	0.569	<0.001
USP4	FANCD2	0.566	<0.001
TBL1XR1	TOPBP1	0.561	<0.001
BRCA1	FANCM	0.561	<0.001
DEK	RFC5	0.560	<0.001
BLM	EME1	0.557	<0.001
ARCN1	CHEK1	0.556	<0.001
CDC73	PALB2	0.554	<0.001
IREB2	FANCI	0.554	<0.001
FANCC	RMI1	0.551	<0.001
BRCA1	FANCD2	0.549	<0.001
BLM	FANCD2	0.549	<0.001
BRCA1	EME1	0.547	<0.001
GVINP1	ZBTB32	0.545	<0.001
HEATR1	PALB2	0.544	<0.001
GPD2	PALB2	0.542	<0.001
BRCA1	RMI1	0.539	<0.001
BRCA1	TOPBP1	0.538	<0.001
FANCC	FANCG	0.538	<0.001
RPA3	CENPS	0.535	<0.001
BLM	BRIP1	0.534	<0.001
CDC73	TOPBP1	0.533	<0.001
IREB2	FANCM	0.530	<0.001
BLM	BRCA1	0.529	<0.001
BRCA1	BLM	0.529	<0.001
BRD1	PALB2	0.520	<0.001

To expound the role of FARGs in prognostic predictive value, we developed univariate Cox regression analyses. The outcomes suggested that *ZBTB32* (HR = 0.186, *p* < 0.001; Table 3), *CENPS* (HR = 0.482, *p* = 0.003), *RFC5* (HR = 0.515, *p* = 0.003), *RPA2* (HR = 0.599, *p* = 0.005), *RMI2* (HR = 0.671, *p* = 0.007), *RPS27AP11* (HR = 0.492, *p* = 0.027), *RFC4* (HR = 0.681, *p* = 0.028), *RAD9A* (HR = 0.603, *p* = 0.036), *TELO2* (HR = 0.576, *p* = 0.043), *PMS2* (HR = 1.997, *p* = 0.015), *RAD50* (HR = 2.107, *p* = 0.006), *HUS1* (HR = 2.981, *p* = 0.001), and *PMS2CL* (HR = 3.551, *p* = 0.002) were the CC prognosis-associated factors for CC OS model fitting, while *ZBTB32* (HR = 0.112, *p* = 0.009), *RFC3* (HR = 1.493, *p* = 0.004), *BRCA2* (HR = 2.810, *p* = 0.010), *PALB2* (HR = 3.142, *p* = 0.024), and *FANCL* (HR = 2.339, *p* = 0.041) were filtered for CC RFS model fitting. Next, LASSO (the last absolute shrinkage and selection operator) Cox regression analyses were performed to select eight OS-related genes and five RFS-related genes, and a risk score named FPS was developed (Figures 2D,G, Supplementary Figures S4B,C). When the patients were divided into high- and low-FPS groups according to the median value of FPS, the Kaplan-Meier survival plots of CC OS (*p* < 0.001) and RFS (*p* < 0.001) showed different trends with the 5-years AUC almost reaching 0.8 (Figures 2E,F; Figures 2H,I). The overlapping of the calibration curve between the predictive values from the nomogram model and the actual observations demonstrated the accuracy of this model (Supplementary Figures S4D,E). Decision curve analysis (DCA) with 2-, 3-, and 5-years was performed and showed that FPS was superior to the other clinical characters (Supplementary Figures S4F,G). The high-FPS group exhibited low expression of *ZBTB32* and *CENPS*, high expression of *PALB2* and *BRCA2*, and reduced OS and RFS (Supplementary Figures S5A,B).

**TABLE 3:** Significant FARGs screened out by univariable cox regression analysis for FPS model fitting.

OS related gene					RFS related gene				
id	HR	HR.95L	HR.95H	*p*-value	id	HR	HR.95L	HR.95H	*p*-value
ZBTB32	0.186	0.072	0.479	0.000	RFC3	2.493	1.342	4.630	0.004
CENPS	0.482	0.296	0.785	0.003	ZBTB32	0.112	0.021	0.583	0.009
RFC5	0.515	0.332	0.800	0.003	BRCA2	2.810	1.283	6.156	0.010
RPA2	0.599	0.419	0.857	0.005	PALB2	3.142	1.163	8.487	0.024
RMI2	0.671	0.503	0.895	0.007	FANCL	2.339	1.034	5.290	0.041
RPS27AP11	0.492	0.262	0.924	0.027					
RFC4	0.681	0.484	0.958	0.028					
RAD9A	0.603	0.376	0.966	0.035					
TELO2	0.576	0.337	0.983	0.043					
PMS2	1.997	1.141	3.495	0.015					
RAD50	2.106	1.241	3.576	0.006					
HUS1	2.980	1.587	5.599	0.001					
PMS2CL	3.551	1.623	7.767	0.002					

Univariate and multivariate Cox regression analyses were performed to assess whether the above elements were independent prognostic factors of clinical pathological features including age, smoking status (SS), HPV status, TNM staging, grade, stage, menopause status (MS), primary therapy outcome (PTO), radiation therapy (RT), and target or molecular therapy (TM). For OS, the results demonstrated that FPS of univariate Cox regression analysis was 4.840 (1.490-9.300) (*p* < 0.001; Figure 2J), while that of multivariate Cox regression analysis was 8.728 (5.410-13.755) (*p* < 0.001; Figure 2L). The results of RFS-associated analysis also suggested that FPS was indeed the only robust CC predictor (*P*s < 0.005, HRs > 1; Figures 2K,M). In addition, by analyzing the expression patterns of FARGs in different FPS CC groups, we found that more FARGs were upregulated in high-FPS group of OS (Supplementary Figure S6C). Conversely, most FARGs were downregulated in the high-FPS group of RFS (Supplementary Figure S6D). Moreover, to test whether the FARG-based prognostic model had predictive function in other cancers, we downloaded information for the head and neck squamous cell carcinoma (HNSC), an HPV-associated cancer and other important gynecological tumors including breast invasive carcinoma (BRCA), ovarian serous cystadenocarcinoma (OV) and uterine corpus endometrial carcinoma (UCEC) to verify FPS-OS model. GSE44001, a CC dataset with patient RFS information, was also downloaded for FPS-RFS model validation. Results indicated that the prognostic model based on FARGs could effectively classify the prognostic risk of the above cancers with good predictive effect (Supplementary Figure S6). These results indicate that FARGs play an essential role in the occurrence and prognosis of CC, and that FPS can be served as a signature for CC prognostic risk prediction.

### Subgroup Identification Based on FARGs-Associated Pognostic Risk Score of FA-Related Genes and Development of FA-Related Genes-Associated Immune Prognostic Risk Score in Cervical Cancer

**FIGURE 3:**
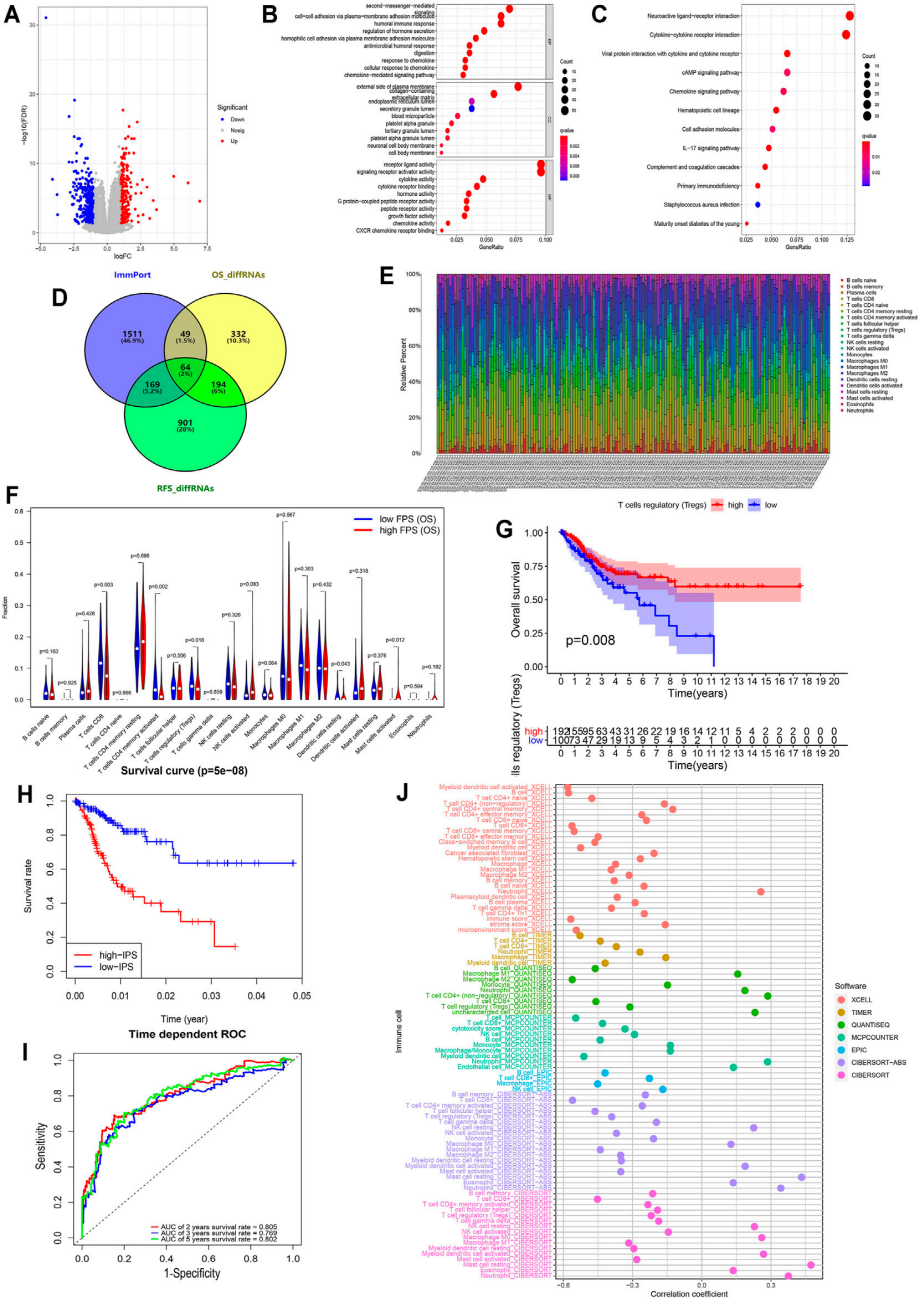
Immune infiltration in CESC among the two risk FPS groups and construction immune signature IPS based on FARGs. **(A)** Volcano plot of DEGs in high-FPS (*n* = 146) compared to low-FPS (n = 146) groups, |log_2_FC| > 1 and *p*-value < 0.05 served as the cutoff. **(B,C)** The GO terms **(B)** and KEGG pathways **(C)** enriched based on the DEGs. **(D)** Venn diagram showing 64 genes contained in the immune gene list from ImmPort Shared Data website, OS-related DEGs and RFS-related DEGs, while 49 OS unique DEGs and 169 RFS unique DEGs were also displayed. **(E)** The stacked column chart exhibited the proportion of 22 immune cells in 309 patients. **(F)** Violin plot showed the abundance and comparison of different immune cells in different FPS groups. **(G)** Kaplan-Meier survival curves for patients with different infiltrated Tregs. **(H)** Kaplan-Meier survival curves for patient OS with high and low IPS based on immune signature. **(I)** The ROC curves based on IPS for 2-, 3-, and 5-years OS probabilities. **(J)** The correlation analysis of IPS and immune pattern acquired by xcell, TIMER, quanTIseq, MCP-counter, EPIC, and CIBERSORT.

To assess the discrepancy of different risk groups, we screened DEGs between low- and high-FPS groups and performed gene ontology (GO) and Kyoto Encyclopedia of Genes and Genomes (KEGG) pathway enrichment analysis of OS and RFS predictive models. A total of the 404 upregulated and 236 downregulated genes in high-FPS groups of OS, 745 upregulated and 583 downregulated genes in high-FPS groups of RFS were identified relative to low-FPS groups, respectively (Figure 3A and Supplementary Figure S7A). Functional enrichment analysis results demonstrated that immune related biological processes contributed significantly (Figures 3B,C; Supplementary Figures S7B,C). Interestingly, when these DEGs were compared with immune-related genes obtained from Immport database, we found that 258 genes could be identified in both OS and RFS patterns (Figure 3D). Besides, 49 OS special immune genes and 169 RFS special immune genes were screened simultaneously.

CIBERSORT was used to evaluate the difference in the abundance of immune infiltration in CC patients (Figure 3E). Correlation analysis illustrated that CD8^+^ T cells were negatively related to CD4^+^ T cells memory resting (Supplementary Figure S7D; R = −0.450, *p* < 0.050) and M0 macrophages (R = −0.510, *p* < 0.050). The comparison of CC cases revealed that CD8 T cells (*p* = 0.003), resting CD4 memory T cells (*p* = 0.002), regulatory T cells (Tregs; *p* = 0.018), resting dendritic cells (*p* = 0.043), and activated mast cells (*p* = 0.012) were significantly different in low- and high-FPS groups for OS (Figure 3F), while CD8 T cells (*p* = 0.033), resting CD4 memory T cells (*p* = 0.025), regulatory T cells (Tregs; *p* = 0.021), and activated dendritic cells (*p* = 0.002) were different for RFS (Supplementary Figure S7E). Kaplan-Meier survival analysis revealed that Tregs infiltration was associate with better OS outcome (*p* = 0.008; Figure 3G) as well as M2 macrophages (*p* < 0.001; Supplementary Figure S7F), activated CD4 memory T cells (*p* = 0.001), CD8 T cells (*p* = 0.004), and resting dendritic cells (*p* = 0.024). In contrast, the high infiltration level of M0 macrophages (*p* = 0.007), activated mast cells (*p* < 0.001), neutrophils (*p* = 0.036), and resting CD4 memory T cells (*p* = 0.009) had no protective action for CC patients, according to these results.

To further probe the potential associations of FARGs and immune processes, key immune-associated genes related to FARGs and CC prognosis were selected for evaluation (Figure 3D). Based on univariate Cox analysis, a total of 50 immune-related genes were identified for OS model fitting, as well as 14 genes for RFS (Supplementary Table S4). Next, LASSO Cox regression analysis identified core immune elements as immune signatures that were used to calculate subsequent risk scores (Supplementary Figures S8A,B). Based on immune characteristics, IPS was calculated through coefficients and gene expression, then CC samples were divided into two different risk groups according to the IPS median. Different trends were observed in the two groups OS, and the 2- and 5-years AUC were larger than 0.8 (Figures 3H,I), while the RFS result indicated that the different trends with 2-, 3-, and 5-years AUC values were close to 0.8 (Supplementary Figures S8C,D). In the OS prediction model, the high-IPS group showed high expression of *CXCL8* and *EREG*, low expression of *CCR7* and *ZAP70*, and reduced OS. In addition, the high-IPS group displayed low expression of *CCL1*, *NCR1,* and *CD244*, high expression of *INSL4*, and reduced RFS in the RFS prediction model (Supplementary Figures S8E,F). In addition, correlation analysis based on FARGs and IPS-associated signatures was developed, and the results revealed that *RFC3*, *ZBTB32*, *BRCA2,* and *RPA2* acted as significant connectors in these two clusters of genes at the mRNA level (Supplementary Figure S7G; Rs > 0.3, *P*s < 0.05), implying that the bridge between FARGs and the immune processes is based on these molecules. At the end of this section, we analyzed the correlation between IPS and the abundance of immune cells calculated by five different methods, including xcell, TIMER, quanTIseq, MCP-counter, and EPIC, to detect the association between IPS and immune infiltration. Figure 3J showed a significant negative association between IPS and the abundance of T cells, B cells, M2 macrophage, and NK cells, but a relatively strong positive correlation was found between neutrophils cells and mast cells abundance. These data demonstrate that FARGs were correlated with the immune genes and IPS could be served as a reliable signature for CC prognostic prediction for both OS and RFS.

### Cluster Identification Based on Consensus Clustering of FA-Related Genes and a FA-Related Gene-Associated Prognostic Adhesion Signature Construction in Cervical Cancer

**FIGURE 4:**
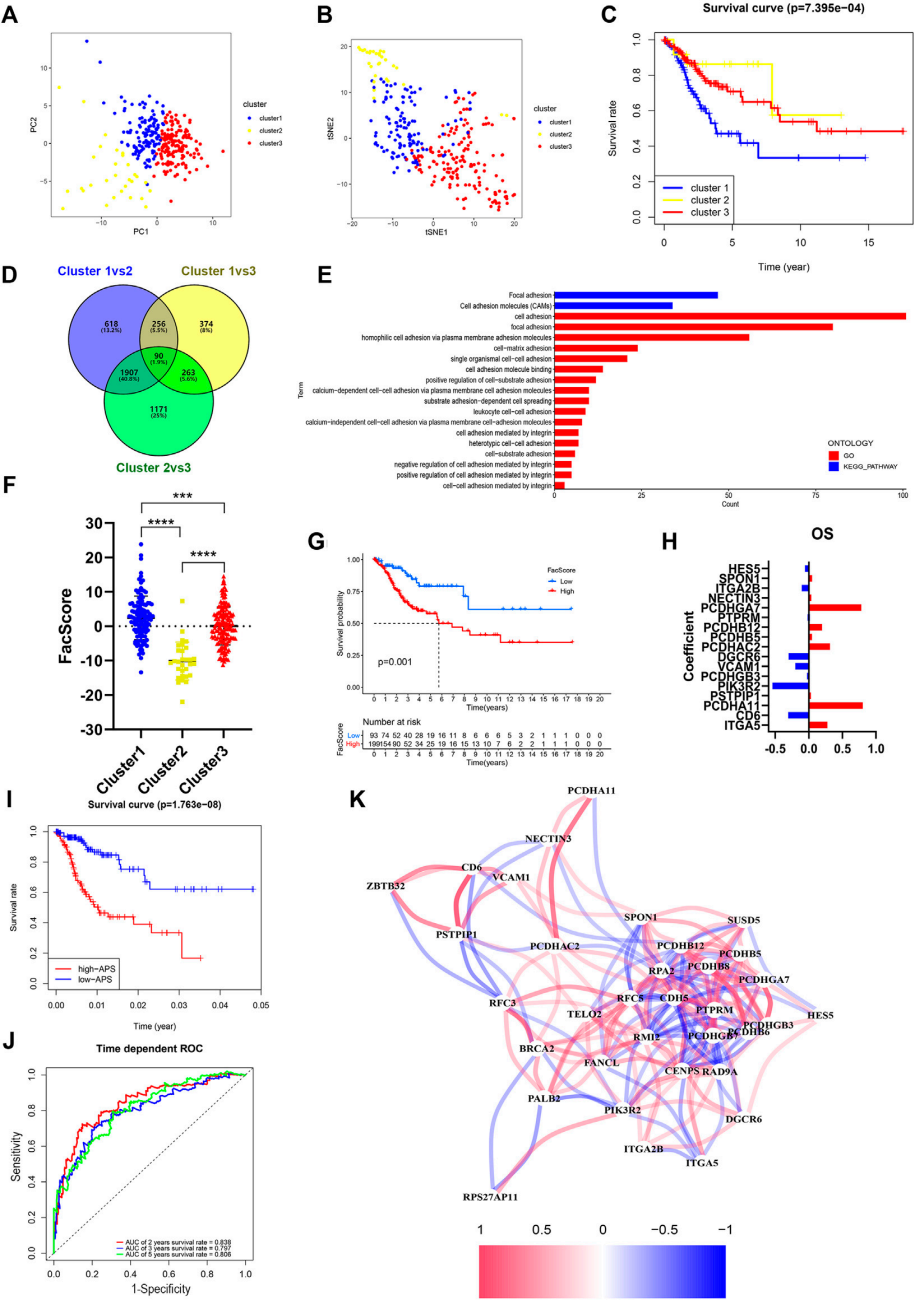
Identification and characteristic description of the consensus cluster and build cell adhesion signature APS based on FARGs. **(A,B)** Consensus clustering for k = 3 based on FARGs expression pattern and visualization by PCA **(A)** and t-SNE **(B)**. **(C)** Kaplan-Meier survival curve for patients in different clusters. **(D)** Venn diagram showing the DEGs obtained from the comparison between each of the two subgroups. The cutoff value is |log_2_FC| > 1 and *p*-value < 0.05. **(E)** The function enrichment result based on the DEGs and the bar chart showing the cell adhesion related pathways. **(F)** Representation of the cluster character by FacScore calculated by adhesion genes expression pattern. *, *p* < 0.05; **, *p* < 0.01; ***, *p* < 0.001. **(G)** Kaplan-Meier survival curves for patients with high and low FacScore. **(H)** The signature coefficients of OS. **(I)** Kaplan-Meier survival curves for patients with high and low APS based on cell adhesion signature. **(J)** ROC curves based on APS for 2-, 3-, and 5-years survival probabilities. **(K)** Correlation network of FARGs and cell adhesion associated genes. PCA, principal component analysis; t-SNE, t-distributed stochastic neighbor embedding.

To determine the potential function of FARGs, consensus clustering analysis was carried out to discriminate the FARG expression patterns and separate CC patients into three subgroups (Supplementary Figure S9A). Patients grouped well in cluster testing by principal component analysis (PCA; Figure 4A) and t-distributed stochastic neighbor embedding (t-SNE; Figure 4B) analysis. Subsequently, Kaplan-Meier survival analysis based on subgroups demonstrated that cluster 1 had the shortest OS trend (*p* < 0.001; Figure 4C), while no significant difference was observed in RFS (*p* = 0.510; Supplementary Figure S9B). To further assess the functions of three clusters, DEGs were screened among clusters and a total of 90 shared DEGs were identified (Supplementary Figure S9C and Figure 4D). Subsequently, functional enrichment analysis was performed based on these intersecting DEGs and cell adhesion related biological processes and signaling pathways were enriched (Figure 4E), which indicated that clusters might have major differences in this aspect. As focal adhesion and cell adhesion moleculars (CAMs) were considered to be highly related to the invasion and metastasis of malignant tumors, we speculated that FARGs might affect the malignant progression of CC by regulating cell adhesion-related pathways. To clarify this hypothesis, an adhesion function score, named Facscore, based on 90 shared DEGs, was calculated to quantitatively describe the cell adhesion degree of each CC sample. Obviously, there were significant differences among clusters: cluster 1 had the highest Facscore and cluster 2 had the lowest score (Figure 4F). According to the median of Facscore, CC patients were divided into two groups with high and low Facscore and then the Kaplan-Meier survival analysis was performed. The result suggested that patients with high Facscore were associated with the poor prognosis of both OS (*p* = 0.001; Figure 4G) and RFS (*p* = 0.028; Supplementary Figure S9D).

As many cell adhesion regulators had been shown to be involved in tumor initiation and evolvement, we performed a global analysis using these 90 DEGs to detect the prognostic predictive values. Univariate Cox analysis showed that 39 regulators were significantly associated with CC OS, while 22 regulators were significantly associated with RFS (Supplementary Table S5). To distinguish more powerful prognostic predictors, the LASSO Cox regression model was constructed, and 17 key elements were identified for OS predictive as well as 6 for RFS (Supplementary Figures S9E,G and Figure 4H). Results demonstrated that high expressions of *PCDHGA7*, *PCDHB12*, *PCDHAC2*, *PCDHA11*, and *ITGA5* were associated with poor OS outcome in CC patients, but *ITGA2B*, *DGCR6*, *VCAM1*, *CD6*, and *PIK3R2* were protective factors. In the RFS model, *PCDHA11*, *PCDHB8*, *PCDHB6*, *PCDHGB7*, *SUSD5*, and *CDH5* were all risk elements for CC patients. In the subsequent analysis, we found that the calculated adhesion-related prognostic risk score (APS) based on the above variables could also be regarded as an essential signature for CC OS prediction (*p* < 0.001) with 2- and 5-years AUC larger than 0.8 (Figures 4I,J). However, no significant difference was observed in RFS analyses (*p* = 0.552; Supplementary Figure S9H), even though the AUC was larger than 0.7 (Supplementary Figure S9I). These results showed that cell adhesion-related regulators had vital prognostic values in CC. To further explore the correlation between FARGs and APS, we developed a network based on mRNA expression (Figure 4K). The results demonstrated that there were extensive correlation connections between FARGs and APS-associated genes, which were mainly regulated by *RFC5*, *RPA2*, *PMI2*, *CENPS*, *ZBTB32*, and *RAD9A* (Rs > 0.3, *P*s < 0.05). These results revealed a non-negligible link between FARGs and cell adhesion-related processes in CC.

### Establishment of a Comprehensive Prognostic Model of Cervical Cancer Based on FA-Related Gene Regulators, Immune Signatures, and Cell Adhesion Indicators

**FIGURE 5:**
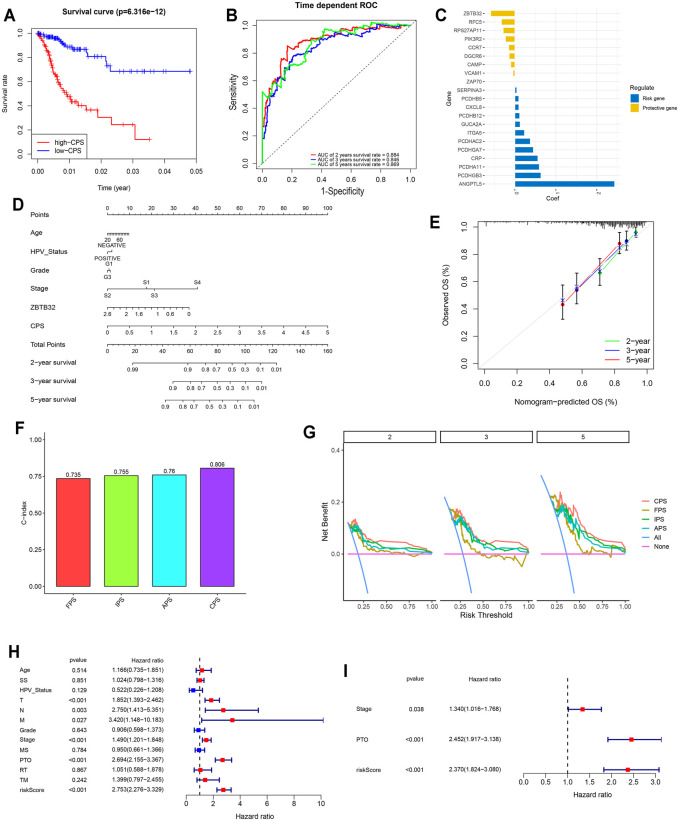
Comprehensive signature was identified based on FARGs and played vital roles in the prognosis of CC. **(A)** Kaplan-Meier survival curves for patients with high and low CPS based on gene signature. **(B)** ROC curves based on CPS for 2-, 3-, and 5-years survival probabilities. **(C)** The detailed coefficient calculated by multivariate Cox regression using LASSO. **(D)** The nomogram prognosis prediction model. **(E)** The calibration plots suggested the comparison between prediction and actual outcome for 2-, 3-, and 5-years survival probabilities in the nomogram model. **(F)** C-index comparison of FPS, IPS, APS, and CPS. **(G)** DCA curves indicated the comparison between four prediction risk scores. **(H,I)** The univariate **(H)** and multivariate **(I)** Cox regression analysis between clinical features and OS. SS, smoking status; MS, menopause status; PTO, primary therapy outcome; RT, radiation therapy; TM, target or molecular therapy.

Since immune signatures and cell adhesion indicators, identified based on analyses of FARGs, had an indispensable role for CC OS prediction, we aimed to integrate signatures and construct a comprehensive prognostic model for CC patient survival risk prediction in a more accurate way (Table 4). According to LASSO Cox analysis, the comprehensive prediction score (CPS) was calculated by 21 of 35 candidate genes and can classify the samples as high- and low-risk, with the 2-, 3-, and 5-years AUC larger than 0.8 (Figures 5A–C, Supplementary Figure S10A). Among these elements, high expression of *CXCL8*, *ITGA5*, *PCDHAC2*, *PCDHA11*, and *CRP* was associated with poor OS outcome, while the increased OS trend was related to high expression of *ZBTB32*, *RFC5*, *VCAM1*, *CAMP*, and *RPS27AP11* (Supplementary Figures S10B,D). Patient information was shown in Table 5, which revealed that CPS had significant differences in the groups containing T (*p* = 0.005), stage (*p* = 0.024), PTO (*p* < 0.001), RT (*p* = 0.010), and TM (*p* < 0.001). When patients were divided by the CPS, FPS, IPS, APS, and clusters, we found a high degree of consistency among these risk groups, and most of the cluster 1 cases were enriched in the high-risk groups (Supplementary Figure S10E).

**TABLE 4:** Significant genes screened out by univariable cox regression analysis for CPS model fitting.

id	HR	HR.95L	HR.95H	*p*-value	Source
ANGPTL5	60.021	6.596	546.162	<0.001	IPS
CAMP	0.632	0.420	0.952	0.028	IPS
CCR7	0.569	0.421	0.769	<0.001	IPS
CD6	0.486	0.339	0.699	<0.001	IPS
CENPS	0.482	0.296	0.785	0.003	FPS
CRP	2.310	1.502	3.554	<0.001	IPS
CXCL8	1.290	1.142	1.457	<0.001	IPS
DGCR6	0.410	0.215	0.783	0.007	APS
EREG	1.401	1.163	1.687	<0.001	IPS
GUCA2A	1.263	1.019	1.565	0.033	IPS
HES5	0.643	0.416	0.992	0.046	APS
ITGA2B	0.684	0.478	0.977	0.037	APS
ITGA5	1.510	1.268	1.797	<0.001	APS
NECTIN3	1.344	1.029	1.755	0.030	APS
PAEP	1.240	1.082	1.421	0.002	IPS
PCDHA11	4.606	1.965	10.794	<0.001	APS
PCDHAC2	2.224	1.234	4.009	0.008	APS
PCDHB12	2.267	1.203	4.272	0.011	APS
PCDHB5	1.864	1.173	2.961	0.008	APS
PCDHGA7	4.978	1.229	20.165	0.025	APS
PCDHGB3	17.254	2.300	129.445	0.006	APS
PIK3R2	0.099	0.021	0.468	0.004	APS
PSTPIP1	0.556	0.387	0.799	0.002	APS
PTPRM	1.368	1.058	1.769	0.017	APS
RAD9A	0.603	0.376	0.966	0.035	FPS
RFC5	0.515	0.332	0.800	0.003	FPS
RMI2	0.671	0.503	0.895	0.007	FPS
RPA2	0.599	0.419	0.857	0.005	FPS
RPS27AP11	0.492	0.262	0.924	0.027	FPS
SERPINA3	1.682	1.191	2.376	0.003	IPS
SPON1	1.211	1.011	1.449	0.037	APS
TELO2	0.576	0.337	0.983	0.043	FPS
VCAM1	0.692	0.531	0.902	0.007	IPS
ZAP70	0.554	0.406	0.756	<0.001	IPS
ZBTB32	0.186	0.072	0.479	<0.001	FPS

**TABLE 5:** Comparison of clinical characteristics based on CPS risk group.

Character	Feature	High	Low	Count	*p*-value
Age	>46	72	69	141	0.815
	<46	74	77	151	
Smoking	Ever	26	25	51	0.205
	Current	33	31	64	
	No	65	76	141	
	Else	22	14	36	
Abortion	Yes	46	48	94	0.708
	No	25	37	62	
	Else	75	61	136	
Pregncy	Yes	120	123	243	0.754
	No	26	23	49	
HPV_Status	Positive	133	141	274	0.083
	Negative	13	5	18	
T	1	57	78	135	**0.005**
	2	26	40	66	
	3	13	4	17	
	4	8	2	10	
	Else	42	22	64	
N	0	50	77	127	0.352
	1	26	28	54	
	Else	70	41	111	
M	0	42	63	105	0.978
	1	5	6	11	
	Else	99	77	176	
Grade	1	8	11	19	0.650
	2	64	63	127	
	3	53	62	115	
	4	0	1	1	
	Else	21	9	30	
Stage	1	73	51	124	**0.024**
	2	23	32	55	
	3	16	18	34	
	4	4	12	16	
	Else	1	4	5	
Menopause_Status	Pre	57	67	124	0.712
	Peri	12	13	25	
	Post	41	38	79	
	Else	36	28	64	
Primary_Therapy_Outcome	Complete Remission/response	57	98	155	**0.000**
	Partial remission/response	9	1	10	
	Stable disease	2	4	6	
	Progressive disease	17	5	22	
	Else	61	38	99	
Radiation_Therapy	Yes	74	99	173	**0.010**
	No	37	27	64	
	Else	35	20	55	
Targeted/Molecular_Therapy	Yes	48	82	130	**0.000**
	No	32	31	63	
	Else	66	33	99	

Bold fonts indicate significant differences between groups (p-value < 0.05).

To assess the survival probabilities of CC patients, we established a nomogram prognostic prediction model based on prognostic predictors including age, smoking status, abortion, pregnancy, HPV status, grade, and stage (Figure 5D). The calibration curve indicated the accuracy (Figure 5E). When the FPS, IPS, APS, and CPS were compared, it was evident that the CPS C-index (C-index = 0.806 for CPS; Figure 5F), decision curve benefit (Figure 5G) as well as the AUC at 2-, 3-, and 5-years survival prediction (Supplementary Figure S10C) were significantly higher than others (C-index = 0.735, 0.755, and 0.76 for FPS, IPS, and APS), suggesting that with the relevant molecules increasing, CPS had an optimal predictive effect. Subsequently, univariate and multivariate Cox regression analyses were performed to assess CPS prognostic predictive role in CC as well as clinical pathological features including age, SS, HPV status, T, N, M, grade, stage, MS, PTO, RT, and TM. Results demonstrated that CPS of univariate Cox regression analysis was 2.753 (2.276-3.329) (*p* < 0.001; Figure 5G), while that of multivariate Cox regression analysis was 2.370 (1.824-3.080) (*p* < 0.001; Figure 5H). Apart from this, PTO was another vital factor for OS in CC.

### Machine-Learning Model Derived Gene Signature Predictive of Cervical Cancer Prognosis

**FIGURE 6:**
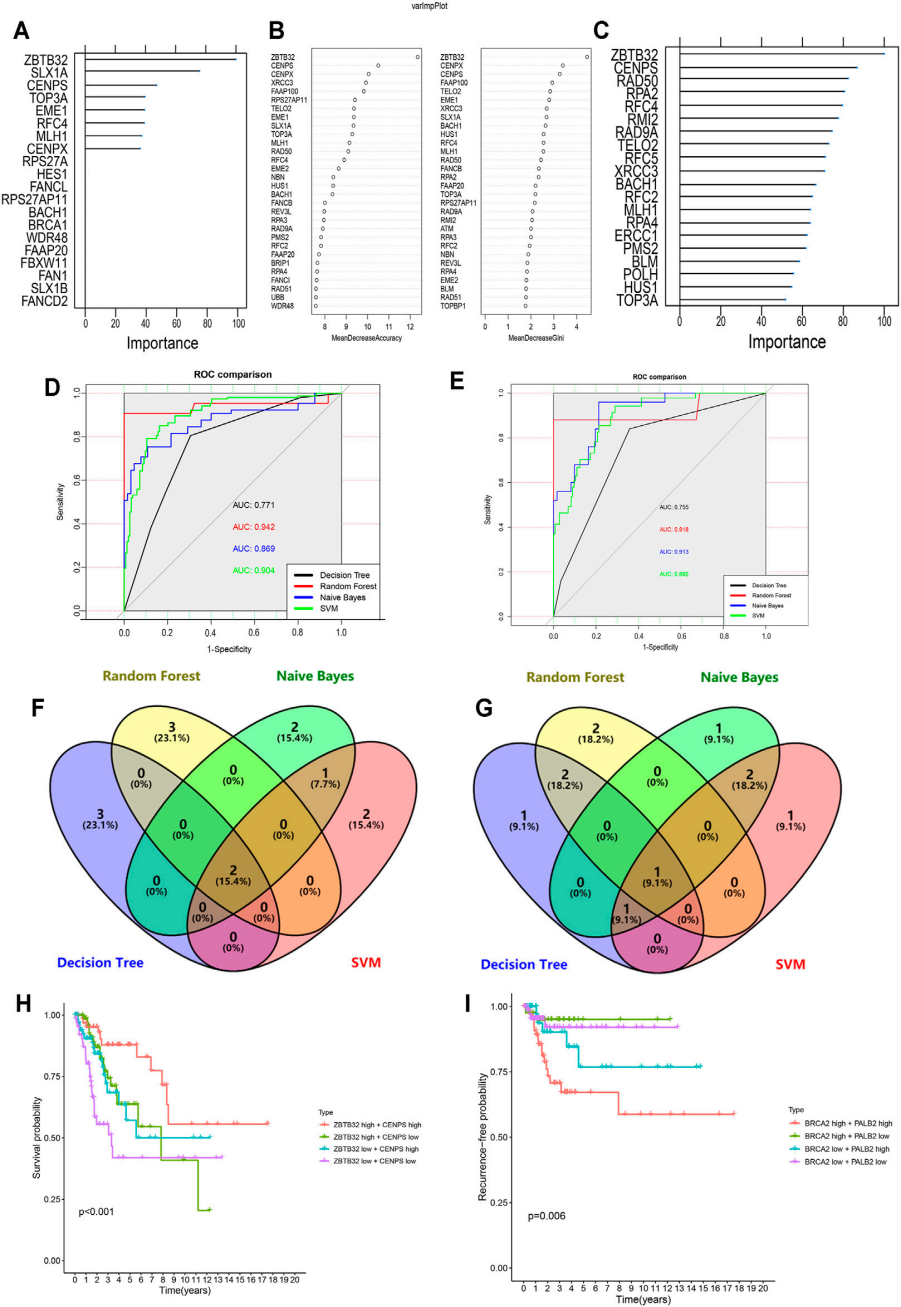
Machine learning-based CC prognostic predictor for both OS and RFS. **(A,C)** Variable importance screening based on decision tree **(A)**, random forest **(B)**, and naïve bayes **(C)** algorithm. **(D,E)** ROC curves based on machine learning methods for OS **(D)** and RFS **(E)** probabilities. **(F,G)** Venn diagram showing the shared candidate genes contained in four machine learning methods for OS **(F)** and RFS **(G)**. **(H)** Multivariate Kaplan-Meier survival curves for patients with different expression of *ZBTB32* and *CENPS*, which were filtered by LASSO-Cox regression algorithm and machine learning methods for OS. **(I)** Multivariate Kaplan-Meier survival curves for patients with different expression of *PALB2* and *BRCA2*, which were filtered by LASSO-Cox regression algorithm and machine learning methods for RFS.

In recent years, the application of machine learning in clinical diseases has been increasing, showing extremely high predictive performance in terms of outcome prediction and variables screening (Bernhofer et al., 2021; Ni et al., 2021; Sundar et al., 2021). To validate our predictive model and its ingredients, machine-learning models were developed based on FARGs. Before commencing, we used the synthetic minority over-sampling technique (SMOTE) method to generate virtual samples to compensate for the data imbalance. Subsequently, 70% of the samples were randomly selected as the training set, and the remaining samples were the validation set, and a fivefold cross-validation method was used in the model-training process. The result of the decision tree (DT) for OS prediction showed that *ZBTB32*, *SLX1A*, *CENPS*, *TOP3A*, and *EME1* were the top five variables among all factors (Figure 6A and Supplementary Figure S11A). *ZBTB32*, *CENPX*, *CENPS*, *FAAP100*, and *TELO2* were selected by random forest (RF; number of trees = 300; Figure 6B and Supplementary Figure S11B). Moreover, *ZBTB32*, *CENPS*, *RAD50*, *RPA2*, and *RFC4* were identified by naïve bayes (NB) model (Figure 6C), and *ZBTB32*, *CENPS*, *RAD50*, *PMS2*, and *HUS1* were recognized using support vector machine model (SVM) of OS prediction (Table 6). The ROC curves showed that the RF model had the highest accuracy (AUC = 0.942; Figure 6D). For the RFS prediction results, the RF model had the highest accuracy at 0.918, followed by the SVM model at 0.914 (Supplementary Figures S11C,G, Table 6 and Figure 6E). The overlapped factors among these algorithms, as well as LASSO Cox regression model, *ZBTB32* and *CENPS* were the identical elements indicating vital predictive values (Figure 6F). *PALB2* was the only shared FARG associated with RFS in CC (Figure 6G). Considering the fact that *PALB2* is an important BRCAx-associated candidate for breast cancer and that evidence indicated that a close interaction exists between *PALB2* and *BRCA2* (Hu et al., 2020), combined with the results that *BRCA2* was recognized by LASSO Cox, DT, SVM, and NB models, we chose *BRCA2* as an RFS predictor.

**TABLE 6:** ROC curve variable importance of SVM model for CC prognosis prediction.

OS		RFS	
Gene	Importance	Gene	Importance
ZBTB32	100.00	PALB2	100.00
CENPS	84.95	RFC3	77.00
RAD50	80.05	BRCA2	75.24
PMS2	78.43	FANCL	60.12
HUS1	64.53	USP1	52.81
FAN1	63.42	ZBTB32	52.74
RFC5	61.15	DCLRE1A	50.52
RFC4	60.95	TOPBP1	46.26
RFC2	55.01	FANCF	43.67
RPA2	54.13	FANCB	40.99
FAH	53.26	RAD1	40.52
RMI2	51.97	PMS2	40.20
MLH1	51.50	REV1	40.15
FANCG	49.54	UBC	37.35
RAD1	49.04	POLI	36.89
BLM	48.84	NBN	36.29
RAD9A	47.96	ATM	35.99
POLH	45.83	FAH	35.15
PMS2CL	45.19	REV3L	32.28
XRCC3	44.72	XRCC3	32.26

Subsequently, the Kaplan-Meier survival analysis was performed on these four CC signatures using the GEPIA tool. The results demonstrated that patients with high levels of *ZBTB32* had longer OS than those with low levels (HR = 0.530, *p* = 0.007; Supplementary Figure S11H). However, although patients with high expression of *CENPS* might have longer OS than those with low expression, the difference was not significant (HR = 0.510, *p* = 0.058; Supplementary Figure S11I). Similarly, the results indicated that *PALB2* and *BRCA2* were both risk factors for DFS in CC, although the difference was not obvious (Supplementary Figures S11J,K). When the survival analysis was carried out using two selected signatures, we found that CC patients with high expression of both *ZBTB32* and *CENPS* had a more distinct tendency to get more OS time than those with low expression, and more significant than analyses of a single FARG (*p* < 0.001; Figure 6H). As for RFS, our results showed that patients with high expressions of *PALB2* and *BRCA2* were more likely to have unfavorable outcomes, but interestingly, groups with low expression of both did not have the best outcomes (*p* = 0.006; Figure 6I). The high-*BRCA2* low-*PALB2* expression population had the highest RFS ratio, suggesting a complicated potential mechanism of CC RFS. These results were derived from the correlation between FARGs and CC clinical outcomes using a machine-learning model to identify the key elements and provide a stable method based on FARGs for CC predicting.

### Further Analysis of Key Signatures in Cervical Cancer

**FIGURE 7:**
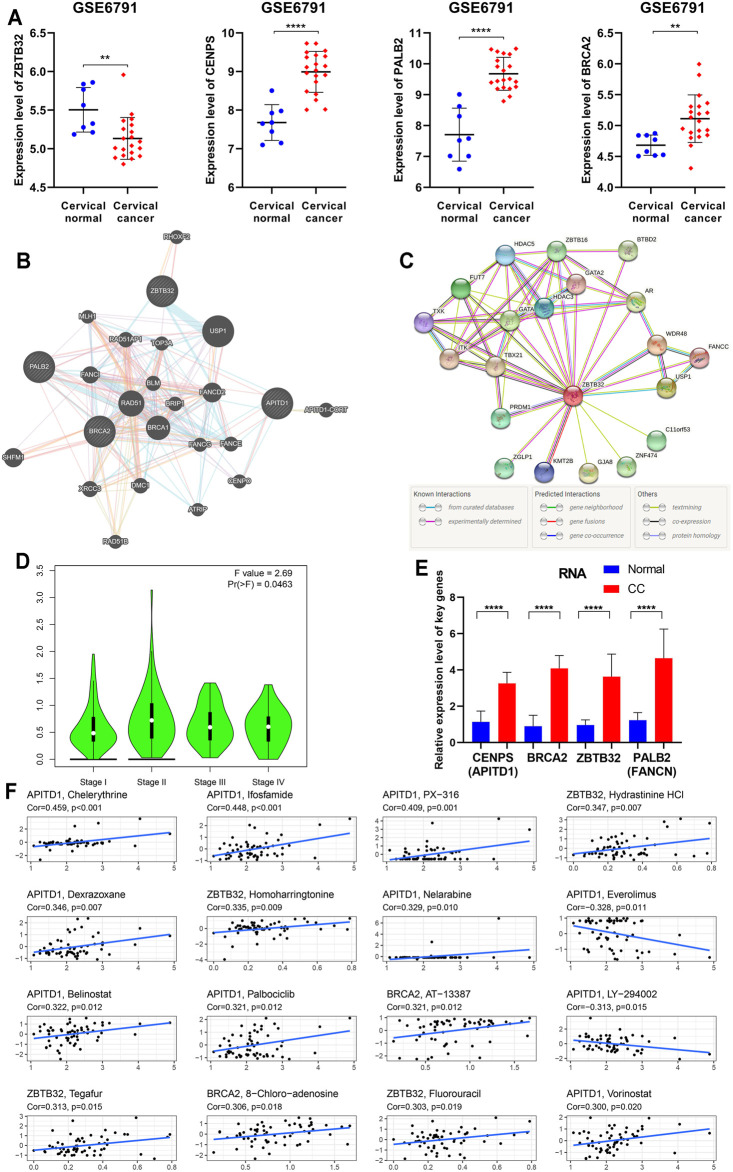
Further bioinformation analyses and validation of the four essential FARGs related to CC prognosis. **(A)** The expression pattern of *ZBTB32*, *PALB2*, *CENPS,* and *BRCA2* between CC patients and normal control at RNA level from GSE6791. **(B)** The PPI network showed the four essential FARGs protein action pattern and associations. **(C)** The PPI network of *ZBTB32*. **(D)** The violin plot showed *ZBTB32* mRNA expression in various clinical stages of CC. **(E)** The mRNA expression and comparison between 19 CC tissues and 22 corresponding normal tissues by qPCR. **(F)** The scatter diagram showed the association between the four FARGs and drugs, which might provide new clues to uncover potential mechanisms for CC prognosis.

To clarify the findings above, we examined the expression of four key FARGs at RNA level and assessed the interaction at protein level. Box plots of GSE6791 showed that *PALB2*, *CENPS*, and *BRCA2* were upregulated in CC patients, but *ZBTB32* was downregulated (Figure 7A). These observations agreed with the results of TCGA (Supplementary Figure S12A) and GSE63514 (Supplementary Figure S12B), except *ZBTB32*, suggesting that *ZBTB32* might not be a stable DEG in CC. Immunohistochemical results also exhibited the abnormal expression of the above targets in cervical tissues (Supplementary Figure S12C). Subsequently, the protein-protein interaction (PPI) network was executed with respect to these four regulators, and it was observed that numerous FARGs and connection with each node existed, but with no direct attachment between the four regulators, except BRCA2 and PALB2 (Figure 7B). When one-center PPI of ZBTB32 was constructed, we studied that a large immune-associated network included GATA, HDAC3, TBX21, in addition to the FA-related network (Figure 7C). However, networks that were highly relevant to the FA pathways were identified only in the PALB2, CENPS, and BRCA2 PPI networks (Supplementary Figure S12D). The results suggested that ZBTB32 might protect CC patients from adverse outcomes mainly by affecting immune-related regulatory processes, rather than merely by affecting FA pathway. Moreover, *ZBTB32* expression was significantly different among CC stages (*p* = 0.046; Figure 7D), suggesting the value of early clinical screening as a biomarker. Unfortunately, the other three were not suitable (*P*s > 0.05; Supplementary Figure S12E). The qPCR results emphasized the difference of four signatures (Figure 7E) in CC. To elucidate the potential mechanism of *ZBTB32* in CC, we developed GSEA analysis to identify the affected pathways. Cell immune and cell adhesion-associated biological processes were enriched in quantity (Supplementary Figures S12F,G, Supplementary Tables S6,7), which agreed with our previous analyses. Considering that studies have regarded the high correlation between key molecule and immune checkpoints as an essential signature in the immune regulation process, we performed a spearman correlation analysis between these four signatures and immune checkpoints. The results showed that *ZBTB32* was significantly correlated with most of the immune checkpoints, but few were observed for the other signatures (Supplementary Figures S12H,I, Supplementary Table S8). Finally, a drug sensitive analysis was carried out to explore the possible clinical diagnosis and treatment methods for *ZBTB32* and other signatures. As Figure 7F shows, the high expression of *CENPS* was positive related to patients resistant to chelerythrine (R = 0.459, *p* < 0.001), ifosfamide (R = 0.448, *p* < 0.001), and PX-316 (R = 0.409, *p* = 0.001), while high expression of *ZBTB32* was associated with hydrastinine HCl (R = 0.347, *p* = 0.007) and homoharringtonine (R = 0.335, *p* = 0.009) resistance. To further investigate the potential function of *ZBTB32* in CC, *ZBTB32* overexpression vector was conducted (Supplementary Figures S13A,B). Through cell transfection and cell phenotype assays, we observed a significant tumor suppressor role for *ZBTB32* in CC cells: *ZBTB32* upregulation caused a decrease in the cell proliferation (*P*s < 0.001; Supplementary Figures S13C,D) and a significant decline in migratory (*P*s < 0.001; Supplementary Figure S13E) and invasive (*P*s < 0.001; Supplementary Figure S13F) abilities of HeLa and SiHa cells. Notably, we detected significant changes in the immune signatures and cell adhesion genes with the upregulation of *ZBTB32* (Supplementary Figures S13G,H).

In summary, these findings supported our assumption that FARGs had important functions in the occurrence, development, and prognosis of CC, and these mechanisms had been proved in the analysis to be achieved by influencing the processes of both immunity and cell adhesion.

## Discussion

In this study, we explored the prognostic value of FARGs in CC and revealed their possible biological mechanisms in CC progression. As an important pathway for repairing DNA damage, the FA pathway has received extensive attention in recent years. However, there are few reports on predicting of CC prognosis using FARGs. Here, we performed a pan-cancer analysis based on FARGs using the GSCA online tool and indicated that FARGs might influence the occurrence, development, and prognosis of CC by affecting HPV genome integration, immune response, and cell adhesion processes. FARG was shown to be differentially expressed and tended to concentrate on similar functional pathways in various cancers (Figures 1B,K). FARGs play an important role in DNA repair, as well as in protecting the human genome during genome integration of viruses such as HPV (Ruiz-Torres et al., 2021; Sauter et al., 2021). Based on this information, we found that some sites in FARGs were HPV integration hot-spots, and expression of FARGs was moderately or strongly correlated with the expression of the genes where the integration hot-spots were located. CC, a severe disease caused by persistent infection with HPV, was selected for primary analysis. We identified the important FARGs in the CC prognostic model and grouped patients into three clusters according to FARG expression. The groups with different FPS differed mainly in immune processes, while the differences between the three clusters were mainly in cellular adhesion. According to our results, *ZBTB32* and *CENPS* were regarded as protective elements for OS, while *PALB2* and *BRCA2* acted as risk factors for RFS in CC, but these results were not all in agreement with previous studies in carcinoma, perhaps due to different molecular mechanisms and different genetic mutations in patients (Krona et al., 2004; Breast Cancer Association et al., 2021; Padella et al., 2021; Satyananda et al., 2021). Unexpectedly, we identified that *ZBTB32* could function as an early signature for CC clinical treatment. Moreover, we found that *ZBTB32* might be regarded as a common indicator for both OS and RFS according to LASSO Cox analysis, DT, RF, and SVM models, though not the naïve bayes model. Importantly, in predicting the function of *ZBTB32*, we found that it might be involved in both immune processes and cell adhesion pathways, which were shown in analyses of FARGs. Studies indicated that ZBTB32 acts as a master regulator of virus-driven NK cell proliferation, which can promote the proliferation outbreak of NK cells after receiving the signal from IRF8 when virus is exposed (Adams et al., 2018). Although a few studies have demonstrated that ZBTB32 plays an important role in immune processes, little research can be found indicating that FA pathway is related to cell adhesion. Subsequently, we constructed a comprehensive model for CC outcome prediction by integrating elements from FPS, IPS, and APS, with over 90% accuracy. In addition, we also performed drug sensitivity analysis for four molecules, including *ZBTB32*, to predict drug resistance as a function of gene expression and to provide a theoretical basis for chemotherapeutic treatment. Several drugs containing chelerythrine and hydrastinine HCl were found to be strongly correlated with *ZBTB32* and *CENPS*.

In the present study, we investigated the prognostic value and potential regulatory mechanism of FARGs in CC using the LASSO Cox model, machine-learning models, and consensus clustering analysis. *ZBTB32* played a crucial role in CC prognosis by influencing immune-related processes and cell adhesion pathways. Research demonstrates that *CENPS* can promote cell apoptosis by combining with p53, which has a strong anti-tumor effect in neuroblastoma (Krona et al., 2004). Due to the powerful tumor suppressor function, we found that patients with high expressions of both had longer OS. It has been reported that the closely connected roles of *BRCA2* and *PALB2* are of great significance in the process of anti-gene mutation, and the frequent mutation and downregulation of them may affect the efficiency of DNA repair and lead to poor outcomes (Pan et al., 2020; Golan et al., 2021). In the pan-cancer analysis, we served *BRCA2* as an esFARG, and *BRCA2* had the most frequent mutation (29%; Figure 1D). As one of the gsFARGs, the mutation frequency of *PALB2* was only 6%, but it ranks sixth among a large number in this group (Supplementary Figure S2D). Unexpectedly, Kaplan-Meier analysis demonstrated that when both *PALB2* and *BRCA2* expression were high, CC patients had significantly shorter RFS. However, the population with high expression of *BRCA2* and low expression of *PALB2* had a longer RFS time. We speculated that this complex result might be due to the different degrees of mutation of the two genes in different patients. Interestingly, we identified a pseudogene, *RPS27AP11* (Gene ID: 728590), in the OS model. Although there is no report about *RPS27AP11*, relevant research has been sufficient to indicate the indispensable regulatory mechanism of pseudogenes in cancers. Hu et al. identified that pseudogene *SUMOP13* depletion in HCC cells can restrain cell growth and lung metastasis by upregulated expression of E-cadherin, an epithelial marker, and downregulated the expression of the mesenchymal marker vimentin as well as *MMP-2* and *MMP-9* (Hu et al., 2021). Jing et al. showed that *NAMPTP1* (nicotinamide phosphoribosyltransferase pseudogene 1) can act as an miRNA target, ultimately affecting the prognosis of pancreatic cancer by participating in an *NAMPTP1*/*HCG11*-*hsa-miR-26b-5p*-*COL12A* competing endogenous RNA network (Jing et al., 2020). These studies suggested a potential mechanism of pseudogenes in organisms, which might provide new ideas for future molecular exploration.

We identified a 10-immune gene signature containing *GUCA2A*, *CAMP*, *SERPINA3*, *PAEP*, *EREG*, *ANGPTL5*, *CCR7*, *ZAP70*, *CRP*, and *CXCL8* for CC OS prediction; *CCR7*, *CAMP*, *ZAP70*, *SERPINA3*, *CXCL8*, *ANGPTL5,* and *CRP* were retained in the CPS construction. According to the immune infiltration analysis based on CC patients from TCGA, T cell function was the main characteristic of immune cells, related to the longer OS time (Supplementary Figure S6F). As a common receptor in lymphoid tissue, CCR7 plays an important role in the activation of T and B cells (Gollmer et al., 2009; Zhao et al., 2014). Studies have shown that CCR7 can activate the migration of memory T cells to inflammatory tissues, which is vital for the immune process. This may explain our results, in which activation of CD8^+^ T cells, as well as the high abundance of memory T cells, significantly prolongs the OS time of CC patients. Surprisingly, the results showed that high Treg infiltration was significantly associated with good prognosis of patients, which was quite opposite to the phenomenon widely reported thus far (Banerjee et al., 2021; Dahlhoff et al., 2021; Peng et al., 2021). In previous reports, little literature has mentioned that Treg can participate in the immune process as a protective factor. Studies have shown that Treg is a protective factor for the reconstitution of immune function (Gaardbo et al., 2014), in patients with extremely scarce immune system. We speculate that Treg may have dual roles in cancer, but the specific regulatory mechanism still needs to be reported in subsequent experiments. ZAP70 is a key signaling molecule of T cells. It has been reported that the enzyme encoded by ZAP70 can be recognized by T cell antigen receptor to phosphorylate and activate signal transduction, which couples the activated T cells with downstream signal pathway (zum Buschenfelde et al., 2010). It is worth noting that *ZAP70* deletion can cause T cell deficiency diseases with CD8^+^ T cell selective deletion (Negishi et al., 1995; Wiest et al., 1997). In addition, *CXCL8* is closely related to the formation of tumor growth environment. It has been reported that CXCL8 inhibits the cell communication between cancer tissue and stroma, as well as the angiogenesis activity of endothelial cells (Lee et al., 2015). As an immune-associated signature, *CXCL8* has been revealed to affect the prognosis of CC through the *circRNAs*-*miRNAs*-*CXCL8* network (Yan et al., 2021). Our study illustrated the outstanding prediction effects of 10-immune signature, revealing the essential role of FARGs and immune regulation in CC.

In a similar process, consensus clustering analysis based on FARGs was performed to separate the CC population into three subgroups with different Facscore. Cell adhesion is a series of processes which are widely studied and related to tumor evolution. However, few studies have examined the relationship between cell adhesion and FA pathway. In our model, the protocadherin family (PCDH) is abundant in terms of OS or RFS. Studies have shown that PCDHs are mainly expressed in the nervous system (Wang et al., 2002), and previous studies have shown that PCDH19 can be regulated by TBR2 and further affect the expansion of mammalian cortical coordinated neurogenesis and the precise assembly of microcircuits (Lv et al., 2019). In the field of cancer, Gao et al. found that PCDH10 can directly participate in the negative regulation of the EGFR/Akt/β-Catenin signaling pathway, thereby inhibiting the occurrence and development of colorectal cancer (Jao et al., 2021). In conclusion, limited studies imply that the potential regulatory mechanisms of PCDHs in different physiological and pathological processes need to be elucidated.

The limitations of this study should be considered. First, to fully explain the possible effects of FARGs in various cancers, we tried to identify the genes related to the FA pathway obtained from GSEA, PathCards, and other databases, instead of taking the intersection of them or screening the core members. Although more variables can be included in the pan-cancer analysis, it is easy to identify the non-core FARGs as the key elements in the model construction process of CC. It is certain that our model is reliable and accurate based on FARGs for CC prognosis prediction. In addition, in order to solve the problem of CC sample groups imbalance, we used the SMOTE method to generate many virtual samples, expanding the group of adverse outcomes, which will greatly improve the efficiency of machine learning, but also leads to doubts about the authenticity of data and the feasibility of application. Unfortunately, we were unable to find other suitable datasets for external validation. The findings in this paper need to be validated in other datasets and prospective experiment examinations.

## Conclusion

In summary, our study provided a systematic analysis based on FARGs in TCGA patients and discovered the prognostic value of FARGs, which showed good accuracy. Moreover, immune processes and cell adhesion pathways are revealed to be involved in the regulation of CC prognosis. This analysis also provides clues for clinical treatment.

## Data Availability

The datasets presented in this study can be found in online repositories. The names of the repository/repositories and accession number(s) can be found below: https://www.ncbi.nlm.nih.gov/geo/, GSE6791/63514/44001, https://portal.gdc.cancer.gov/, TCGA.
