# Supplementary material for: Fanconi Anemia Pathway Genes Advance Cervical Cancer via Immune Regulation and Cell Adhesion
Source: Front Cell Dev Biol. 2021 Nov 15;9:734794. doi: 10.3389/fcell.2021.734794 (PMC8634638; doi:10.3389/fcell.2021.734794)
Supplement: Supplementary file 2 [file Table1.docx]

Supplementary Material

# Supplementary Methods

**Cell culture and transient transfection**

Human cervical cancer (CC) cell lines HeLa bought from Keygen Biotech (Nanjing, China) and SiHa bought from the Cell Resource Center, Peking Union Medical College (which is the headquarter of National Infrastructure of Cell Line Resource, NSTI). All cells were cultured in DMEM medium (HyClone, Logan, UT, USA) with 10% fetal bovine serum (Gibico, Carlsbad, CA, USA) and 1% penicillin-streptomycin solution in a humidified 37℃, 5% CO_2_ atmosphere. ZBTB32 overexpression plasmid and pcDNA3.1 empty vector (Generay Biotech, Shanghai, China) were transiently transfected using Lipofectamine 2000 (Invitrogen, Carlsbad, CA, USA) according to manufacturer’s instructions. Empty or overexpression plasmids after the cells were cultured for 48 hours, the cells were collected and RNA extracted or functionally analyzed. Briefly, 5 × 10^5^ cells were plated in 6-well plates for 24 hours and subsequently incubated with the transfection mixture for 48 hours before cell collection and RNA extraction or functional analysis.

The sequence of ZBTB32 over-expression plasmid is as follows (Forward):

| 1  51  101  151  201  251  301  351  401  451  501  551  601  651  701  751  801  851  901  951  1001  1051  1101  1151  1201  1251  1301  1351  1401  1451  1501  1551  1601  1651  1701  1751  1801  1851  1901  1951  2001  2051  2101  2151  2201  2251  2301  2351  2401  2451  2501  2551  2601  2651  2701  2751  2801  2851  2901  2951  3001  3051  3101  3151  3201  3251  3301  3351  3401  3451  3501  3551  3601  3651  3701  3751  3801  3851  3901  3951  4001  4051  4101  4151  4201  4251  4301  4351  4401  4451  4501  4551  4601  4651  4701  4751  4801  4851  4901  4951  5001  5051  5101  5151  5201  5251  5301  5351  5401  5451  5501  5551  5601  5651  5701  5751  5801  5851  5901  5951  6001 | GACGGATCGG GAGATCTCCC GATCCCCTAT GGTGCACTCT CAGTACAATC  TGCTCTGATG CCGCATAGTT AAGCCAGTAT CTGCTCCCTG CTTGTGTGTT  GGAGGTCGCT GAGTAGTGCG CGAGCAAAAT TTAAGCTACA ACAAGGCAAG  GCTTGACCGA CAATTGCATG AAGAATCTGC TTAGGGTTAG GCGTTTTGCG  CTGCTTCGCG ATGTACGGGC CAGATATACG CGTTGACATT GATTATTGAC  TAGTTATTAA TAGTAATCAA TTACGGGGTC ATTAGTTCAT AGCCCATATA  TGGAGTTCCG CGTTACATAA CTTACGGTAA ATGGCCCGCC TGGCTGACCG  CCCAACGACC CCCGCCCATT GACGTCAATA ATGACGTATG TTCCCATAGT  AACGCCAATA GGGACTTTCC ATTGACGTCA ATGGGTGGAG TATTTACGGT  AAACTGCCCA CTTGGCAGTA CATCAAGTGT ATCATATGCC AAGTACGCCC  CCTATTGACG TCAATGACGG TAAATGGCCC GCCTGGCATT ATGCCCAGTA  CATGACCTTA TGGGACTTTC CTACTTGGCA GTACATCTAC GTATTAGTCA  TCGCTATTAC CATGGTGATG CGGTTTTGGC AGTACATCAA TGGGCGTGGA  TAGCGGTTTG ACTCACGGGG ATTTCCAAGT CTCCACCCCA TTGACGTCAA  TGGGAGTTTG TTTTGGCACC AAAATCAACG GGACTTTCCA AAATGTCGTA  ACAACTCCGC CCCATTGACG CAAATGGGCG GTAGGCGTGT ACGGTGGGAG  GTCTATATAA GCAGAGCTCT CTGGCTAACT AGAGAACCCA CTGCTTACTG  GCTTATCGAA ATTAATACGA CTCACTATAG GGAGACCCAA GCTGGCTAGC  GTTTAAACTT AAGCTTGGTA CCGAGCTCGG ATCCGCCACC ATGGAAGAGT  CTGATCAGGG GCACACAGGC GCACTTGCAA CCTGTGCGGG TCATGAGGAC  AAGGCAGGCT GCCCACCTCG CCCGCACCCT CCCCCGGCCC CTCCTGCTCG  GTCTCGGCCC TATGCGTGCT CTGTCTGTGG AAAGAGGTTT TCACTCAAGC  ATCAGATGGA GACGCACTAC CGAGTCCACA CAGGAGAGAA GCCCTTCTCC  TGTAGCCTTT GTCCTCAGCG CTCCCGGGAC TTCTCGGCCA TGACCAAGCA  CCTGCGGACA CACGGGGCCG CTCCGTACCG CTGCTCCCTG TGCGGGGCCG  GCTGTCCCAG CCTGGCCTCC ATGCAGGCGC ACATGCGCGG TCACTCGCCC  AGCCAACTCC CGCCCGGATG GACCATCCGC TCCACCTTCC TCTACTCCTC  CTCGAGGCCG TCTCGGCCCT CGACCTCTCC CTGTTGTCCT TCTTCCTCCA  CCACCTGAGA ATTCTGCAGA TATCCAGCAC AGTGGCGGCC GCTCGAGTCT  AGAGGGCCCG TTTAAACCCG CTGATCAGCC TCGACTGTGC CTTCTAGTTG  CCAGCCATCT GTTGTTTGCC CCTCCCCCGT GCCTTCCTTG ACCCTGGAAG  GTGCCACTCC CACTGTCCTT TCCTAATAAA ATGAGGAAAT TGCATCGCAT  TGTCTGAGTA GGTGTCATTC TATTCTGGGG GGTGGGGTGG GGCAGGACAG  CAAGGGGGAG GATTGGGAAG ACAATAGCAG GCATGCTGGG GATGCGGTGG  GCTCTATGGC TTCTGAGGCG GAAAGAACCA GCTGGGGCTC TAGGGGGTAT  CCCCACGCGC CCTGTAGCGG CGCATTAAGC GCGGCGGGTG TGGTGGTTAC  GCGCAGCGTG ACCGCTACAC TTGCCAGCGC CCTAGCGCCC GCTCCTTTCG  CTTTCTTCCC TTCCTTTCTC GCCACGTTCG CCGGCTTTCC CCGTCAAGCT  CTAAATCGGG GGCTCCCTTT AGGGTTCCGA TTTAGTGCTT TACGGCACCT  CGACCCCAAA AAACTTGATT AGGGTGATGG TTCACGTAGT GGGCCATCGC  CCTGATAGAC GGTTTTTCGC CCTTTGACGT TGGAGTCCAC GTTCTTTAAT  AGTGGACTCT TGTTCCAAAC TGGAACAACA CTCAACCCTA TCTCGGTCTA  TTCTTTTGAT TTATAAGGGA TTTTGCCGAT TTCGGCCTAT TGGTTAAAAA  ATGAGCTGAT TTAACAAAAA TTTAACGCGA ATTAATTCTG TGGAATGTGT  GTCAGTTAGG GTGTGGAAAG TCCCCAGGCT CCCCAGCAGG CAGAAGTATG  CAAAGCATGC ATCTCAATTA GTCAGCAACC AGGTGTGGAA AGTCCCCAGG  CTCCCCAGCA GGCAGAAGTA TGCAAAGCAT GCATCTCAAT TAGTCAGCAA  CCATAGTCCC GCCCCTAACT CCGCCCATCC CGCCCCTAAC TCCGCCCAGT  TCCGCCCATT CTCCGCCCCA TGGCTGACTA ATTTTTTTTA TTTATGCAGA  GGCCGAGGCC GCCTCTGCCT CTGAGCTATT CCAGAAGTAG TGAGGAGGCT  TTTTTGGAGG CCTAGGCTTT TGCAAAAAGC TCCCGGGAGC TTGTATATCC  ATTTTCGGAT CTGATCAAGA GACAGGATGA GGATCGTTTC GCATGATTGA  ACAAGATGGA TTGCACGCAG GTTCTCCGGC CGCTTGGGTG GAGAGGCTAT  TCGGCTATGA CTGGGCACAA CAGACAATCG GCTGCTCTGA TGCCGCCGTG  TTCCGGCTGT CAGCGCAGGG GCGCCCGGTT CTTTTTGTCA AGACCGACCT  GTCCGGTGCC CTGAATGAAC TGCAGGACGA GGCAGCGCGG CTATCGTGGC  TGGCCACGAC GGGCGTTCCT TGCGCAGCTG TGCTCGACGT TGTCACTGAA  GCGGGAAGGG ACTGGCTGCT ATTGGGCGAA GTGCCGGGGC AGGATCTCCT  GTCATCTCAC CTTGCTCCTG CCGAGAAAGT ATCCATCATG GCTGATGCAA  TGCGGCGGCT GCATACGCTT GATCCGGCTA CCTGCCCATT CGACCACCAA  GCGAAACATC GCATCGAGCG AGCACGTACT CGGATGGAAG CCGGTCTTGT  CGATCAGGAT GATCTGGACG AAGAGCATCA GGGGCTCGCG CCAGCCGAAC  TGTTCGCCAG GCTCAAGGCG CGCATGCCCG ACGGCGAGGA TCTCGTCGTG  ACCCATGGCG ATGCCTGCTT GCCGAATATC ATGGTGGAAA ATGGCCGCTT  TTCTGGATTC ATCGACTGTG GCCGGCTGGG TGTGGCGGAC CGCTATCAGG  ACATAGCGTT GGCTACCCGT GATATTGCTG AAGAGCTTGG CGGCGAATGG  GCTGACCGCT TCCTCGTGCT TTACGGTATC GCCGCTCCCG ATTCGCAGCG  CATCGCCTTC TATCGCCTTC TTGACGAGTT CTTCTGAGCG GGACTCTGGG  GTTCGAAATG ACCGACCAAG CGACGCCCAA CCTGCCATCA CGAGATTTCG  ATTCCACCGC CGCCTTCTAT GAAAGGTTGG GCTTCGGAAT CGTTTTCCGG  GACGCCGGCT GGATGATCCT CCAGCGCGGG GATCTCATGC TGGAGTTCTT  CGCCCACCCC AACTTGTTTA TTGCAGCTTA TAATGGTTAC AAATAAAGCA  ATAGCATCAC AAATTTCACA AATAAAGCAT TTTTTTCACT GCATTCTAGT  TGTGGTTTGT CCAAACTCAT CAATGTATCT TATCATGTCT GTATACCGTC  GACCTCTAGC TAGAGCTTGG CGTAATCATG GTCATAGCTG TTTCCTGTGT  GAAATTGTTA TCCGCTCACA ATTCCACACA ACATACGAGC CGGAAGCATA  AAGTGTAAAG CCTGGGGTGC CTAATGAGTG AGCTAACTCA CATTAATTGC  GTTGCGCTCA CTGCCCGCTT TCCAGTCGGG AAACCTGTCG TGCCAGCTGC  ATTAATGAAT CGGCCAACGC GCGGGGAGAG GCGGTTTGCG TATTGGGCGC  TCTTCCGCTT CCTCGCTCAC TGACTCGCTG CGCTCGGTCG TTCGGCTGCG  GCGAGCGGTA TCAGCTCACT CAAAGGCGGT AATACGGTTA TCCACAGAAT  CAGGGGATAA CGCAGGAAAG AACATGTGAG CAAAAGGCCA GCAAAAGGCC  AGGAACCGTA AAAAGGCCGC GTTGCTGGCG TTTTTCCATA GGCTCCGCCC  CCCTGACGAG CATCACAAAA ATCGACGCTC AAGTCAGAGG TGGCGAAACC  CGACAGGACT ATAAAGATAC CAGGCGTTTC CCCCTGGAAG CTCCCTCGTG  CGCTCTCCTG TTCCGACCCT GCCGCTTACC GGATACCTGT CCGCCTTTCT  CCCTTCGGGA AGCGTGGCGC TTTCTCATAG CTCACGCTGT AGGTATCTCA  GTTCGGTGTA GGTCGTTCGC TCCAAGCTGG GCTGTGTGCA CGAACCCCCC  GTTCAGCCCG ACCGCTGCGC CTTATCCGGT AACTATCGTC TTGAGTCCAA  CCCGGTAAGA CACGACTTAT CGCCACTGGC AGCAGCCACT GGTAACAGGA  TTAGCAGAGC GAGGTATGTA GGCGGTGCTA CAGAGTTCTT GAAGTGGTGG  CCTAACTACG GCTACACTAG AAGAACAGTA TTTGGTATCT GCGCTCTGCT  GAAGCCAGTT ACCTTCGGAA AAAGAGTTGG TAGCTCTTGA TCCGGCAAAC  AAACCACCGC TGGTAGCGGT TTTTTTGTTT GCAAGCAGCA GATTACGCGC  AGAAAAAAAG GATCTCAAGA AGATCCTTTG ATCTTTTCTA CGGGGTCTGA  CGCTCAGTGG AACGAAAACT CACGTTAAGG GATTTTGGTC ATGAGATTAT  CAAAAAGGAT CTTCACCTAG ATCCTTTTAA ATTAAAAATG AAGTTTTAAA  TCAATCTAAA GTATATATGA GTAAACTTGG TCTGACAGTT ACCAATGCTT  AATCAGTGAG GCACCTATCT CAGCGATCTG TCTATTTCGT TCATCCATAG  TTGCCTGACT CCCCGTCGTG TAGATAACTA CGATACGGGA GGGCTTACCA  TCTGGCCCCA GTGCTGCAAT GATACCGCGA GACCCACGCT CACCGGCTCC  AGATTTATCA GCAATAAACC AGCCAGCCGG AAGGGCCGAG CGCAGAAGTG  GTCCTGCAAC TTTATCCGCC TCCATCCAGT CTATTAATTG TTGCCGGGAA  GCTAGAGTAA GTAGTTCGCC AGTTAATAGT TTGCGCAACG TTGTTGCCAT  TGCTACAGGC ATCGTGGTGT CACGCTCGTC GTTTGGTATG GCTTCATTCA  GCTCCGGTTC CCAACGATCA AGGCGAGTTA CATGATCCCC CATGTTGTGC  AAAAAAGCGG TTAGCTCCTT CGGTCCTCCG ATCGTTGTCA GAAGTAAGTT  GGCCGCAGTG TTATCACTCA TGGTTATGGC AGCACTGCAT AATTCTCTTA  CTGTCATGCC ATCCGTAAGA TGCTTTTCTG TGACTGGTGA GTACTCAACC  AAGTCATTCT GAGAATAGTG TATGCGGCGA CCGAGTTGCT CTTGCCCGGC  GTCAATACGG GATAATACCG CGCCACATAG CAGAACTTTA AAAGTGCTCA  TCATTGGAAA ACGTTCTTCG GGGCGAAAAC TCTCAAGGAT CTTACCGCTG  TTGAGATCCA GTTCGATGTA ACCCACTCGT GCACCCAACT GATCTTCAGC  ATCTTTTACT TTCACCAGCG TTTCTGGGTG AGCAAAAACA GGAAGGCAAA  ATGCCGCAAA AAAGGGAATA AGGGCGACAC GGAAATGTTG AATACTCATA  CTCTTCCTTT TTCAATATTA TTGAAGCATT TATCAGGGTT ATTGTCTCAT  GAGCGGATAC ATATTTGAAT GTATTTAGAA AAATAAACAA ATAGGGGTTC  CGCGCACATT TCCCCGAAAA GTGCCACCTG ACGTC |
| --- | --- |

**RNA extraction and qRT-PCR**

Total RNA was isolated by TRIzol reagent (Invitrogen) and subsequently reverse transcribed with application of cDNA Reverse Transcription Kit (Vazyme Biotech, Nanjing, China). Real-time PCR was carried out using SYBR® Green PCR kit (Vazyme Biotech, Nanjing, China). The housekeeping gene GAPDH was used as loading controls. Premier sequences were listed in Table S2.

**CCK-8 assays and Transwell assays**

The transfected cells (5 × 10^3^ cells/well) were seeded in 96-well plates and cells of each group were set up in parallel with 6 duplicate holes. At 24, 36, 48 or 60 hours respectively, 10μl CCK-8 reagent (Vazyme Biotech, Nanjing, China) was added to each plate for another 2 hours of incubation at 37°C. The absorbance was then tested at a wavelength of 450 nm (OD 450 value). For invasion assays, after 48 hours of transfection, 4×10^5^ cells were added to the upper compartment of Matrigel-coated (BD, Franklin Lakes, NJ, USA) invasion chambers (8-mm pore size, Corning, New York, USA) and 600 μL of DMEM supplemented with 15% FBS was added to the lower chamber. After 24 hours, cells at the bottom of the chamber were fixed with 100% methanol and stained with 0.1% crystal violet. Air dried cells counted under a microscope and five randomly fields were counted randomly in each well. Migration was also measured according to the above method without Matrigel.

**Immunohistochemistry staining**

Human CC tissues were used for immunohistochemistry experiments, and the adjacent tissues from this patient were used as normal tissue controls. ZBTB32 and PALB2 antibody used were purchased from Novus Biologicals (NBP2-62707, NBP2-50232; US) at a dilution of 1:500. After the staining was completed, scanning analysis was performed using CaseViewer v2.4 software, and the proportion of brown areas was used as the basis for judging the expression amount of target proteins in each tissue.

# Supplementary Figure Legends

**Figure S1 Pan-cancer analyses of extremely significant FARGs among tumors.**

A) The RNA pattern of RPS27AP11 among 33 cancers. B) The association between OS and methylation level. C-D) Drug sensitive analysis between extremely significant FARGs expression obtained from cancer cell lines and GDSC (C) and CTRP (D) database.

**Figure S2 Pan-cancer analyses of general significant FARGs among tumors.**

A-B) The PPI network (A) and the edge numbers involved in network (B). C) The relation between FARGs and overall survival among cancers. D) Type and frequency analysis of CNV mutations in extremely significant FARGs (Top 10). E) Pearson correlation between CNV and RNA expression. F) General significant FARGs methylation level comparison between cancers and normal samples. G) The association between OS and methylation level. H) Spearman correlation analyses of gene methylation and expression. I) The essential pathway enrichment analyses of general significant FARGs by GSVA. J-K) Drug sensitive analysis between extremely significant FARGs expression obtained from cancer cell lines and GDSC (J) and CTRP (K) database.

**Figure S3 Pan-cancer analyses of not significant FARGs among tumors.**

A-B) The PPI network (A) and the edge numbers involved in network (B). C) The relation between FARGs and overall survival among cancers. D) Type and frequency analysis of CNV mutations in extremely significant FARGs (Top 10). E) Pearson correlation between CNV and RNA expression. F) Not significant FARGs methylation level comparison between cancers and normal samples. G) The association between OS and methylation level. H) Spearman correlation analyses of gene methylation and expression. I) The essential pathway enrichment analyses of not significant FARGs by GSVA. J-K) Drug sensitive analysis between extremely significant FARGs expression obtained from cancer cell lines and GDSC (J) and CTRP (K) database.

**Figure S4 Establishment of FPS in CC.**

A) The correlation between HPV integration hot pot genes and FARGs. B-C) The fitting process of LASSO-Cox regression for both OS (B) and RFS (C). D-E) The nomogram prognosis prediction model containing Age, HPV_Status, Grade, Stage, and FPS for both OS (D, left) and RFS (E, left). The calibration plots suggested the comparison between prediction and actual outcome for 2-, 3-, and 5-year survival probabilities in the nomogram model for both OS (D, right) and RFS (E, right). F-G) Decision curve analysis for the evaluation of the net benefits of FPS, Age, HPV_Status, Grade, and Stage at 2-, 3-, and 5-year for both OS model (F) and RFS model (G).

**Figure S5 Survival curves and heatmap of candidate genes.**

A-B) Distribution of the FPS, survival status, and the relative expression of candidate genes among CC patients for both OS (A) and RFS (B). C-D) The heatmap showed the different expression level of FARGs in both OS (C) and RFS (D) groups.

**Figure S6 Validation of FPS in HPV-related cancers and major gynecologic tumors.**

A-D) Kaplan-Meier survival curves and ROC curves showed a reasonable discriminative effect of FPS-OS in both BRCA (A), HNSC (B), OV (C) and UCEC (D). E) The FPS-RFS validation by GSE4401, a CC dataset.

**Figure S7 Immune infiltration in CESC among the two risk FPS groups.**

A) Volcano plot of DEGs in high-FPS (n = 117) compared to low-FPS (n = 117) groups, |log2FC| > 1 and P-value < 0.05 served as the cutoff. B-C) The GO terms (B) and KEGG pathways (C) enriched based on the DEGs. D) Spearman correlation analysis of the 22 immune cells in 309 CC patients. E) Violin plot showed the abundance and comparison of different immune cells in different FPS groups. F) Kaplan-Meier survival curves for patients with significant infiltrated features (P < 0.05). G) Spearman correlation analysis among FARGs and IPS-associated signatures at RNA level (Rs > 0.3, Ps < 0.05).

**Figure S8 Construction immune signature IPS based on FARGs.**

A-B) The fitting process of LASSO-Cox regression for both OS (A) and RFS (B) based on immune signatures. C) Kaplan-Meier survival curves for patients RFS with high and low IPS based on immune signature. D) The ROC curves based on IPS for 2-, 3- and 5-year RFS probabilities. E-F) Distribution of the IPS, survival status, and the relative expression of candidate genes among CC patients for both OS (E) and RFS (F).

**Figure S9 Identification and characteristic description of the consensus cluster and build cell adhesion signature APS based on FARGs.**

A) Line chart showed the optimal number selection and the change of total within sum of square. B) Kaplan-Meier RFS curve for patients in different clusters. C) Volcano plot of DEGs in each of the two clusters comparison. D) Kaplan-Meier RFS curves for patients with high and low FacScore. E-F) The fitting process of LASSO-Cox regression for both OS (E) and RFS (F) based on cell adhesion signatures. G) The signature coefficients of RFS. H) Kaplan-Meier survival curves for patients RFS with high and low APS based on immune signature. I) The ROC curves based on APS for 2-, 3- and 5-year RFS probabilities.

**Figure S10 Comprehensive signature was identified based on FARGs and played vital roles in the prognosis of CC.**

A) The fitting process of LASSO-Cox regression for CC OS based on the FPS-, IPS- and APS- signatures. B) Distribution of the CPS, OS, survival status, and the relative expression of candidate genes among CC patients. C) ROC curve comparison at 2-, 3-, and 5-year survival probabilities. D) Heatmap showed FARGs expression pattern in low- and high- CPS groups. E) Heatmap showed that FARGs expression pattern in CC clinical features including FPS, IPS, APS and CPS.

**Figure S11 Machine learning-based CC prognostic predictor for both OS and RFS.**

A) The decision tree for OS prognostic model fitting of CC patients based on FARGs. B) The process of tree number determination in random forest fitting process for OS outcome. C) The decision tree for RFS prognostic model fitting of CC patients based on FARGs. D-E) Variable importance screening based on decision tree (D) and naïve bayes (E) algorithm fitting process. F) The process of tree number determination in random forest fitting process for RFS outcome. G) Variable importance screening based on random forest algorithm fitting progress. H-I) Kaplan-Meier OS curves for patients with ZBTB32 (H) and CENPS (I). J-K) Kaplan-Meier RFS curves for patients with PALB2 (J) and BRCA2 (K).

**Figure S12 Further bioinformation analyses and validation of the four essential FARGs related to CC prognosis.**

A) The expression pattern of ZBTB32, PALB2, CENPS, and BRCA2 between CC patients and normal control at RNA level from TCGA-CESC. B) The expression pattern of ZBTB32, PALB2, CENPS, and BRCA2 between CC patients and normal control at RNA level from GSE63514. C) The expression pattern of ZBTB32, PALB2, and BRCA2 between CC tissues and paired normal tissues by immunohistochemistry. D) The PPI networks of CENPS, PALB2, and BRCA2. E) The violin plots showed CENPS, PALB2, and BRCA2 mRNA expression in various clinical stages of CC. F-G) GSEA analyses of ZBTB32 using GO terms (F) and KEGG pathways (G). H-I) Spearman correlation between ZBTB32, CENPS, PALB2, and BRCA2 mRNA expression level and immune checkpoints (H) and P values (I).

**Figure S13 ZBTB32 overexpression inhibited CC cells proliferation.**

A) Structural diagram of ZBTB32 overexpression plasmid. B) QRT-PCR determined expression efficiency of transfection with ZBTB32 in HeLa and SiHa cells. C-D) The CCK-8 assay was conducted to measure cell proliferation in HeLa (C) and SiHa (D) cells after transfection with ZBTB32. E-F) Transwell assays were used for testing the migration and invasion ability of HeLa and SiHa cells after transfection with ZBTB32. G-H) QRT-PCR was performed for detecting key genes expression. OE, overexpression; * *P* < 0.05, ** *P* < 0.01, *** *P* < 0.001, **** *P* < 0.0001.

# Supplementary Tables

## Table S1. Details of human tissue samples used for qPCR assays

| **ID** | **Type** | **Age** | **Gender** | **Pathology** |
| --- | --- | --- | --- | --- |
| C01 | Ca | 72 | female | Uterine squamous cell carcinoma stage IIA1 |
| C02 | Ca | 41 | female | Right pelvic lymph node showed cancer metastasis 1 / 1 |
| C03 | Ca | 53 | female | Squamous cell carcinoma grade II-III; >Full thickness of 2 / 3 cervix; left pelvic lymph node can be seen cancer metastasis 1 / 12 |
| C04 | Ca | 49 | female | Squamous cell carcinoma grade III, the depth of cancer tissue invasion approximately 9 / 13 of the full thickness of the cervical wall, visible multifocal intravascular tumor thrombus; left and right pelvic cavity, left and right common iliac, presacral lymph node cancer metastasis T1B2N1 |
| C05 | Ca | 74 | female | Squamous cell carcinoma; full thickness of the muscular wall of the cervical canal (outer 1 / 2) |
| C06 | Ca | 66 | female | Squamous cell carcinoma Grade I |
| C07 | Ca | 46 | female | Squamous cell carcinoma; 1 / 2 layer within fibromuscular layer |
| C08 | Ca | 53 | female | Squamous cell carcinoma; stage IB1 |
| C09 | Ca | 53 | female | Squamous cell carcinoma; full thickness of cervical wall |
| C10 | Ca | 77 | female | Invasive squamous cell carcinoma |
| C11 | Ca | 50 | female | Cervical squamous cell carcinoma |
| C12 | Ca | 32 | female | Cervical squamous cell carcinoma |
| C13 | Ca | 34 | female | Cervical squamous cell carcinoma |
| C14 | Ca | 64 | female | Cervical squamous cell carcinoma |
| C15 | Ca | 57 | female | Cervical squamous cell carcinoma |
| C16 | Ca | 53 | female | Cervical squamous cell carcinoma |
| C17 | Ca | 66 | female | Cervical squamous cell carcinoma |
| C18 | Ca | 45 | female | Cervical squamous cell carcinoma |
| C19 | Ca | 43 | female | Cervical squamous cell carcinoma |
| N01 | N | 48 | female | Fibroid, normal CC tissue |
| N02 | N | 63 | female | Fibroid, normal CC tissue |
| N03 | N | 42 | female | Fibroids; cavity occupation, endometrial polyps; chronic cervicitis |
| N04 | N | 54 | female | Fibroid; intrauterine ring |
| N05 | N | 43 | female | Multiple fibroids in uterus; cyst in left ovary |
| N06 | N | 46 | female | Fibroid, normal CC tissue |
| N07 | N | 61 | female | Uterine prolapse III degree |
| N08 | N | 47 | female | Fibroids; adenomyosis |
| N09 | N | 41 | female | Chronic inflammation, squamous metaplasia |
| N10 | N | 50 | female | Paracancerous tissues |
| N11 | N | 32 | female | Paracancerous tissues |
| N12 | N | 34 | female | Normal cervical tissue |
| N13 | N | 62 | female | Fibroid, normal CC tissue |
| N14 | N | 64 | female | Normal cervical tissue |
| N15 | N | 57 | female | Paracancerous tissues |
| N16 | N | 53 | female | Paracancerous tissues |
| N17 | N | 66 | female | Paracancerous tissues |
| N18 | N | 53 | female | Normal cervical tissue |
| N19 | N | 45 | female | Paracancerous tissues |
| N20 | N | 43 | female | Paracancerous tissues |
| N21 | N | 48 | female | Normal cervical tissue |
| N22 | N | 49 | female | Uterine fibroid, normal cervical cancer tissue |

## Table S2. Primer sequences of key signatures for qPCR

| **GeneSymbol** | **Premier_Sequence** |  |
| --- | --- | --- |
| ZBTB32 | TGGCTCTGATCGGCTGGTACAG | Forward |
|  | CTCCTGGCTCCCTACGGTGATC | Reverse |
| ZAP70 | GAAGGCAGACACGGAAGAGATGATG | Forward |
|  | CCAATGAGCCGCACGATGTAGG | Reverse |
| VCAM1 | ACACACAGGTGGGACACAAATAAGG | Forward |
|  | GCTCCAAGGATCACGACCATCTTC | Reverse |
| RPA2 | CTGCTTTCTGCCACTTTGGTTGATG | Forward |
|  | ACAATGTTGGTTGGAGCCTTCTCTG | Reverse |
| PSTPIP1 | TGCGTTCTGTTCTCCTTGGTGTG | Forward |
|  | CTTCCCTTGTTCCTTTGCCTCCTC | Reverse |
| RFC3 | AGCCTCTGGGTGGACAAGTATCG | Forward |
|  | GCACCTGATGGTCCGTACACTAAC | Reverse |
| PCDHGB3 | GGGCAGAGGCGAATGCTATTTCTC | Forward |
|  | GGAATAGCGTAGCGGATCGGTTC | Reverse |
| PCDHGA7 | AAGCAAGAGAAGCAGGACGACTATG | Forward |
|  | AGGAGGATGGAGAGCAGGAAGAATC | Reverse |
| PCDHB5 | AGGTACAGGTGCCCGAGAACAG | Forward |
|  | TGGTTGAGTAACTTCATCGCCTTGG | Reverse |
| PCDHB12 | GAGCAGAGCCGAGTACAACATCAC | Forward |
|  | CGAACAGGGCGTAGGAAGTTTGG | Reverse |
| PCDHAC2 | CGGGAGTTCTTTGTGACTGTGGAG | Forward |
|  | GGCTGCTGACGAGTTGGTTGAG | Reverse |
| PCDHA11 | CCGTTCGGCTGTTAGTCCAAGTC | Forward |
|  | TCGGTCTGTGGCGTTTAGTTTGAG | Reverse |
| NECTIN3 | AGGTGGGCAGGGTGTAGAATGG | Forward |
|  | CGGCTTTCAGTCAATAGGGTCTTCC | Reverse |
| ITGA5 | CCTCCCAATTTCAGACTCCCATTCC | Forward |
|  | CCGTCAGCACCTTCAAGAAGTACC | Reverse |
| ITGA2B | GCAATTCTAGCCACCATGAGTCCAG | Forward |
|  | CTCCTCCTCCTTCCCTTCAGATTCC | Reverse |
| CXCL8 | AACTTTCAGAGACAGCAGAGCACAC | Forward |
|  | CACACAGTGAGATGGTTCCTTCCG | Reverse |
| CENPS | ACAGAGGCTAAAGGCAGCAGT | Forward |
|  | GCCGCAATGGTCTGTTTGCT | Reverse |
| CD6 | CCACCGACAACGATGACTACGATG | Forward |
|  | GGAAAGGGAGTAGGTAGAGCAGGAG | Reverse |
| CD244 | GTGGCTCTGTCTTGCTTGGTCTC | Forward |
|  | CTCGTCCAGGTAGGTGAGGTTCC | Reverse |
| CCR7 | GCTGTGGTCGTGGTCTTCATAGTC | Forward |
|  | AGGCGATGTTGAGTTGCTTACTGAG | Reverse |
| CCL1 | CGCCTTGGACACAGTTGGATGG | Forward |
|  | GAATGGTGTAGGGCTGGTAGTTTCG | Reverse |
| CAMP | ATGCTAACCTCTACCGCCTCCTG | Forward |
|  | CAATCCTCTGGTGACTGCTGTGTC | Reverse |
| BRCA2 | AGGGAAGCTTCATAAGTCAGTCTCA | Forward |
|  | TGAAGCATCTGATACCTGGACAGA | Reverse |
| PALB2 | GGATCTCTCACCGCAGCTAA | Forward |
|  | ACTCAGGCCCAACATCAAGT | Reverse |
| β-actin | ATCCGCAAAGACCTGT | Forward |
|  | GGGTGTAACGCAACTAAG | Reverse |

## Table S3. GSVA analysis of FARGs in cervical cancer data

| **id** | **logFC** | ***P*-value** | **adj.*P*.Val** |
| --- | --- | --- | --- |
| GOCC_NUCLEOLUS | 0.387 | 0.000 | 0.000 |
| HP_HYPERTONIA | 0.366 | 0.000 | 0.000 |
| HP_UPPER_MOTOR_NEURON_DYSFUNCTION | 0.366 | 0.000 | 0.000 |
| HP_ABNORMALITY_OF_THE_SYNOVIA | -0.244 | 0.000 | 0.000 |
| HP_JOINT_DISLOCATION | -0.236 | 0.000 | 0.001 |
| HP_LOWER_EXTREMITY_JOINT_DISLOCATION | -0.236 | 0.000 | 0.001 |
| HP_ABNORMALITY_OF_LOWER_LIMB_JOINT | -0.236 | 0.000 | 0.001 |
| HP_ABNORMALITY_OF_THE_CALVARIA | -0.258 | 0.000 | 0.001 |
| GOMF_ENDONUCLEASE_ACTIVITY | 0.310 | 0.000 | 0.001 |
| HP_ABNORMAL_SIZE_OF_THE_PALPEBRAL_FISSURES | -0.229 | 0.000 | 0.001 |
| HP_ABNORMAL_PENIS_MORPHOLOGY | -0.254 | 0.000 | 0.001 |
| HP_ABNORMALITY_OF_THE_URETHRA | -0.254 | 0.000 | 0.001 |
| HP_PES_PLANUS | -0.254 | 0.000 | 0.001 |
| HP_HYPERTROPHIC_CARDIOMYOPATHY | -0.255 | 0.000 | 0.001 |
| HP_ABNORMAL_BONE_STRUCTURE | -0.255 | 0.000 | 0.001 |
| HP_ABNORMALITY_OF_BONE_MINERAL_DENSITY | -0.255 | 0.000 | 0.001 |
| HP_FACIAL_ASYMMETRY | -0.264 | 0.000 | 0.001 |
| HP_SHORT_LONG_BONE | -0.264 | 0.000 | 0.001 |
| HP_UPPER_LIMB_UNDERGROWTH | -0.264 | 0.000 | 0.001 |
| HP_LIMB_UNDERGROWTH | -0.264 | 0.000 | 0.001 |
| HP_ABNORMAL_FOREARM_BONE_MORPHOLOGY | -0.264 | 0.000 | 0.001 |
| HP_DISPLACEMENT_OF_THE_URETHRAL_MEATUS | -0.264 | 0.000 | 0.001 |
| HP_ABNORMALITY_OF_GLOBE_SIZE | -0.234 | 0.000 | 0.001 |
| HP_ABNORMALITY_OF_LONG_BONE_MORPHOLOGY | -0.229 | 0.000 | 0.001 |
| HP_HYPERREFLEXIA | -0.242 | 0.000 | 0.001 |
| HP_HYDROURETER | -0.269 | 0.000 | 0.001 |
| HP_HYDROCEPHALUS | -0.269 | 0.000 | 0.001 |
| HP_PROPTOSIS | -0.269 | 0.000 | 0.001 |
| HP_BICORNUATE_UTERUS | -0.269 | 0.000 | 0.001 |
| HP_TRIPHALANGEAL_THUMB | -0.269 | 0.000 | 0.001 |
| HP_CLUBBING | -0.269 | 0.000 | 0.001 |
| HP_ABNORMAL_ILEUM_MORPHOLOGY | -0.269 | 0.000 | 0.001 |
| HP_ABNORMALITY_OF_THE_AMNIOTIC_FLUID | -0.269 | 0.000 | 0.001 |
| HP_OLIGOHYDRAMNIOS | -0.269 | 0.000 | 0.001 |
| HP_SIDEROBLASTIC_ANEMIA | -0.269 | 0.000 | 0.001 |
| HP_ABNORMALITY_OF_THE_SMALL_INTESTINE | -0.269 | 0.000 | 0.001 |
| HP_ABNORMAL_DUODENUM_MORPHOLOGY | -0.269 | 0.000 | 0.001 |
| HP_AGANGLIONIC_MEGACOLON | -0.269 | 0.000 | 0.001 |
| HP_ABNORMALITY_OF_FEMUR_MORPHOLOGY | -0.269 | 0.000 | 0.001 |
| HP_ABNORMALITY_OF_THE_CEREBROSPINAL_FLUID | -0.269 | 0.000 | 0.001 |
| HP_PYRIDOXINE_RESPONSIVE_SIDEROBLASTIC_ANEMIA | -0.269 | 0.000 | 0.001 |
| HP_APLASIA_HYPOPLASIA_OF_THE_ULNA | -0.269 | 0.000 | 0.001 |
| HP_EXTERNAL_EAR_MALFORMATION | -0.269 | 0.000 | 0.001 |
| HP_ABNORMALITY_OF_THE_PHALANGES_OF_THE_TOES | -0.269 | 0.000 | 0.001 |
| HP_APLASIA_HYPOPLASIA_OF_THE_UVULA | -0.269 | 0.000 | 0.001 |
| HP_ABSENT_TESTIS | -0.269 | 0.000 | 0.001 |
| HP_ANEMIA_OF_INADEQUATE_PRODUCTION | -0.269 | 0.000 | 0.001 |
| HP_ABNORMAL_SHAPE_OF_THE_FRONTAL_REGION | -0.269 | 0.000 | 0.001 |
| HP_SHORT_PALPEBRAL_FISSURE | -0.269 | 0.000 | 0.001 |
| HP_ABNORMAL_URETER_MORPHOLOGY | -0.269 | 0.000 | 0.001 |
| HP_ABNORMALITY_OF_THE_TESTIS_SIZE | -0.269 | 0.000 | 0.001 |
| HP_ARTERIOVENOUS_MALFORMATION | -0.269 | 0.000 | 0.001 |
| HP_ABNORMALITY_OF_THE_PREPUTIUM | -0.269 | 0.000 | 0.001 |
| HP_CLUBBING_OF_TOES | -0.269 | 0.000 | 0.001 |
| HP_DUODENAL_STENOSIS | -0.269 | 0.000 | 0.001 |
| HP_ABNORMAL_SHAPE_OF_THE_PALPEBRAL_FISSURE | -0.269 | 0.000 | 0.001 |
| HP_ABNORMALITY_OF_THE_PERIPHERAL_NERVOUS_SYSTEM | -0.269 | 0.000 | 0.001 |
| HP_ABNORMALITY_OF_THE_URETER | -0.259 | 0.000 | 0.001 |
| HP_ABNORMALITY_IRIS_MORPHOLOGY | -0.259 | 0.000 | 0.001 |
| HP_ABNORMALITY_OF_PRENATAL_DEVELOPMENT_OR_BIRTH | -0.259 | 0.000 | 0.001 |
| HP_TETRALOGY_OF_FALLOT | -0.259 | 0.000 | 0.001 |
| HP_PATENT_DUCTUS_ARTERIOSUS | -0.259 | 0.000 | 0.001 |
| HP_ABNORMAL_AORTIC_VALVE_MORPHOLOGY | -0.259 | 0.000 | 0.001 |
| HP_ABNORMAL_HEART_VALVE_MORPHOLOGY | -0.259 | 0.000 | 0.001 |
| HP_ABNORMALITY_OF_THE_SPINAL_CORD | -0.259 | 0.000 | 0.001 |
| HP_TRACHEOESOPHAGEAL_FISTULA | -0.259 | 0.000 | 0.001 |
| HP_ABNORMAL_TRACHEA_MORPHOLOGY | -0.259 | 0.000 | 0.001 |
| HP_ABNORMAL_CAROTID_ARTERY_MORPHOLOGY | -0.259 | 0.000 | 0.001 |
| HP_SPINAL_DYSRAPHISM | -0.259 | 0.000 | 0.001 |
| HP_ABNORMAL_CONNECTION_OF_THE_CARDIAC_SEGMENTS | -0.259 | 0.000 | 0.001 |
| HP_CONGENITAL_MALFORMATION_OF_THE_GREAT_ARTERIES | -0.259 | 0.000 | 0.001 |
| HP_ABNORMAL_LOCALIZATION_OF_KIDNEY | -0.259 | 0.000 | 0.001 |
| HP_ABNORMAL_NEURAL_TUBE_MORPHOLOGY | -0.259 | 0.000 | 0.001 |
| HP_APLASIA_HYPOPLASIA_AFFECTING_THE_EYE | -0.231 | 0.000 | 0.001 |
| HP_ABNORMALITY_OF_THE_FOREARM | -0.223 | 0.000 | 0.001 |
| HP_HYPERPIGMENTATION_OF_THE_SKIN | -0.247 | 0.000 | 0.001 |
| HP_ABNORMAL_MYOCARDIUM_MORPHOLOGY | -0.241 | 0.000 | 0.002 |
| HP_ABNORMALITY_OF_THE_UVULA | -0.258 | 0.000 | 0.002 |
| HP_ABNORMAL_SOFT_PALATE_MORPHOLOGY | -0.258 | 0.000 | 0.002 |
| HP_ABNORMAL_UVEA_MORPHOLOGY | -0.247 | 0.000 | 0.002 |
| HP_GAIT_DISTURBANCE | 0.313 | 0.000 | 0.002 |
| HP_RENAL_INSUFFICIENCY | -0.232 | 0.000 | 0.002 |
| HP_ABNORMAL_RENAL_PHYSIOLOGY | -0.232 | 0.000 | 0.002 |
| HP_IRREGULAR_HYPERPIGMENTATION | -0.244 | 0.000 | 0.003 |
| HP_ABNORMAL_JOINT_MORPHOLOGY | -0.208 | 0.000 | 0.003 |
| HP_ABNORMALITY_OF_TOE | -0.207 | 0.000 | 0.003 |
| HP_ABNORMALITY_OF_PELVIC_GIRDLE_BONE_MORPHOLOGY | -0.207 | 0.000 | 0.003 |
| HP_ABNORMALITY_OF_THE_CURVATURE_OF_THE_VERTEBRAL_COLUMN | -0.207 | 0.000 | 0.003 |
| HP_CRYPTORCHIDISM | -0.216 | 0.000 | 0.004 |
| HP_HYPOPIGMENTATION_OF_THE_SKIN | -0.254 | 0.000 | 0.004 |
| HP_ABNORMAL_LOWER_LIMB_BONE_MORPHOLOGY | -0.211 | 0.000 | 0.004 |
| HP_ABNORMALITY_OF_THE_ABDOMINAL_WALL | -0.223 | 0.000 | 0.004 |
| HP_ABNORMAL_REFLEX | -0.214 | 0.000 | 0.005 |
| HP_ABNORMAL_FINGER_PHALANX_MORPHOLOGY | -0.219 | 0.000 | 0.005 |
| HP_ABNORMALITY_OF_THE_LOWER_URINARY_TRACT | -0.220 | 0.000 | 0.006 |
| HP_ABNORMAL_UMBILICUS_MORPHOLOGY | -0.227 | 0.000 | 0.006 |
| HP_ABNORMAL_CARDIAC_SEPTUM_MORPHOLOGY | -0.227 | 0.000 | 0.006 |
| HP_ABNORMAL_AORTIC_MORPHOLOGY | -0.227 | 0.000 | 0.006 |
| HP_ABNORMAL_CARDIAC_ATRIUM_MORPHOLOGY | -0.227 | 0.000 | 0.006 |
| HP_APLASIA_HYPOPLASIA_AFFECTING_THE_ANTERIOR_SEGMENT_OF_THE_EYE | -0.227 | 0.000 | 0.006 |
| HP_RENAL_HYPOPLASIA_APLASIA | -0.227 | 0.000 | 0.006 |
| HP_ABNORMAL_SYSTEMIC_ARTERIAL_MORPHOLOGY | -0.227 | 0.000 | 0.006 |
| HP_ABNORMALITY_OF_THE_CURVATURE_OF_THE_CORNEA | -0.227 | 0.000 | 0.006 |
| HP_HERNIA | -0.227 | 0.000 | 0.006 |
| HP_SLOPING_FOREHEAD | -0.228 | 0.000 | 0.006 |
| HP_ABNORMALITY_OF_THE_FOREHEAD | -0.197 | 0.000 | 0.006 |
| HP_ABNORMAL_ESOPHAGUS_MORPHOLOGY | -0.243 | 0.000 | 0.006 |
| HP_ASTIGMATISM | -0.234 | 0.000 | 0.006 |
| HP_TRIANGULAR_SHAPED_PHALANGES_OF_THE_HAND | -0.234 | 0.000 | 0.006 |
| HP_DECREASED_FERTILITY | -0.250 | 0.000 | 0.007 |
| HP_DOLICHOCEPHALY | -0.250 | 0.000 | 0.007 |
| HP_HYPOPIGMENTED_SKIN_PATCHES | -0.250 | 0.000 | 0.007 |
| HP_MYELODYSPLASIA | -0.250 | 0.000 | 0.007 |
| HP_DECREASED_FERTILITY_IN_MALES | -0.250 | 0.000 | 0.007 |
| HP_ABNORMALITY_OF_THE_LENS | -0.208 | 0.000 | 0.007 |
| HP_OCULAR_ANTERIOR_SEGMENT_DYSGENESIS | -0.208 | 0.000 | 0.007 |
| HP_FUNCTIONAL_ABNORMALITY_OF_MALE_INTERNAL_GENITALIA | -0.218 | 0.000 | 0.007 |
| HP_ABNORMAL_MALE_REPRODUCTIVE_SYSTEM_PHYSIOLOGY | -0.218 | 0.000 | 0.007 |
| HP_NEUROLOGICAL_SPEECH_IMPAIRMENT | 0.299 | 0.000 | 0.007 |
| HP_ABNORMALITY_OF_GLOBE_LOCATION | -0.211 | 0.000 | 0.007 |
| HP_ABNORMAL_PERIPHERAL_NERVOUS_SYSTEM_MORPHOLOGY | 0.294 | 0.000 | 0.007 |
| HP_ABNORMALITY_OF_SKIN_PIGMENTATION | -0.217 | 0.000 | 0.008 |
| HP_ABNORMALITY_OF_THE_CHOANAE | -0.231 | 0.000 | 0.008 |
| HP_ABNORMAL_THROMBOCYTE_MORPHOLOGY | -0.231 | 0.000 | 0.008 |
| HP_ABNORMAL_ERYTHROCYTE_MORPHOLOGY | -0.231 | 0.000 | 0.008 |
| HP_ANAL_ATRESIA | -0.231 | 0.000 | 0.008 |
| HP_ABNORMALITY_OF_THE_ANUS | -0.231 | 0.000 | 0.008 |
| HP_ABNORMAL_UPPER_LIMB_BONE_MORPHOLOGY | -0.192 | 0.000 | 0.010 |
| HP_APLASIA_HYPOPLASIA_OF_THE_RADIUS | -0.223 | 0.000 | 0.010 |
| HP_APLASIA_HYPOPLASIA_INVOLVING_FOREARM_BONES | -0.223 | 0.000 | 0.010 |
| HP_CLINICAL_COURSE | 0.286 | 0.000 | 0.010 |
| HP_ABNORMAL_TESTIS_MORPHOLOGY | -0.193 | 0.000 | 0.010 |
| HP_ABNORMAL_EXTERNAL_GENITALIA | -0.193 | 0.000 | 0.010 |
| HP_ABNORMALITY_OF_THE_LIVER | -0.195 | 0.000 | 0.013 |
| HP_EPICANTHUS | -0.217 | 0.000 | 0.014 |
| HP_ABNORMALITY_OF_THE_ULNA | -0.217 | 0.000 | 0.014 |
| HP_INTRAUTERINE_GROWTH_RETARDATION | -0.196 | 0.000 | 0.014 |
| HP_FINGER_SYNDACTYLY | -0.225 | 0.000 | 0.015 |
| HP_APLASIA_HYPOPLASIA_OF_FINGERS | -0.225 | 0.000 | 0.015 |
| HP_ABNORMAL_CRANIAL_NERVE_PHYSIOLOGY | -0.225 | 0.000 | 0.015 |
| HP_ABNORMAL_THUMB_MORPHOLOGY | -0.194 | 0.000 | 0.017 |
| HP_ABNORMALITY_OF_THE_UTERUS | -0.215 | 0.000 | 0.017 |
| HP_ABNORMAL_UTERUS_MORPHOLOGY | -0.215 | 0.000 | 0.017 |
| HP_ABNORMALITY_OF_THE_OUTER_EAR | -0.190 | 0.000 | 0.018 |
| HP_ABNORMALITY_OF_THE_CEREBRAL_VENTRICLES | -0.212 | 0.000 | 0.018 |
| HP_VENTRICULOMEGALY | -0.212 | 0.000 | 0.018 |
| HP_ABNORMALITY_OF_THE_PALPEBRAL_FISSURES | -0.181 | 0.000 | 0.018 |
| HP_ABNORMAL_SPERMATOGENESIS | -0.220 | 0.000 | 0.020 |
| GOBP_MITOTIC_DNA_INTEGRITY_CHECKPOINT | 0.226 | 0.000 | 0.021 |
| HP_ABNORMAL_VASCULAR_MORPHOLOGY | -0.204 | 0.000 | 0.022 |
| HP_ABNORMAL_MORPHOLOGY_OF_THE_GREAT_VESSELS | -0.204 | 0.000 | 0.022 |
| HP_HYPERTELORISM | -0.208 | 0.000 | 0.025 |
| HP_TOE_SYNDACTYLY | -0.208 | 0.000 | 0.025 |
| HP_RECURRENT_URINARY_TRACT_INFECTIONS | -0.209 | 0.000 | 0.029 |
| GOMF_ENDODEOXYRIBONUCLEASE_ACTIVITY | 0.255 | 0.000 | 0.029 |
| HP_UPSLANTED_PALPEBRAL_FISSURE | -0.203 | 0.000 | 0.032 |
| GOBP_REGULATION_OF_TELOMERE_MAINTENANCE | 0.267 | 0.000 | 0.033 |
| HP_LEUKOPENIA | -0.199 | 0.000 | 0.037 |
| HP_APLASIA_HYPOPLASIA_INVOLVING_BONES_OF_THE_UPPER_LIMBS | -0.195 | 0.000 | 0.039 |
| HP_HYPOGONADISM | -0.181 | 0.000 | 0.046 |

## Table S4. Significant FARGs screened out by univariable cox regression analysis for IPS model fitting

| **OS related gene** | | | | |  | **RFS related gene** | | | | |
| --- | --- | --- | --- | --- | --- | --- | --- | --- | --- | --- |
| **id** | **HR** | **HR.95L** | **HR.95H** | ***P*-value** |  | **id** | **HR** | **HR.95L** | **HR.95H** | ***P*-value** |
| ANGPTL5 | 60.021 | 6.596 | 546.162 | 0.000 |  | REG1A | 1.281 | 1.067 | 1.538 | 0.008 |
| C8G | 0.585 | 0.369 | 0.930 | 0.023 |  | BMP3 | 1.377 | 1.064 | 1.781 | 0.015 |
| CAMP | 0.632 | 0.420 | 0.952 | 0.028 |  | CCL1 | 0.000 | 0.000 | 0.239 | 0.019 |
| CCL19 | 0.816 | 0.713 | 0.933 | 0.003 |  | INSL4 | 1.597 | 1.074 | 2.377 | 0.021 |
| CCL5 | 0.820 | 0.714 | 0.941 | 0.005 |  | CLEC4M | 0.002 | 0.234 | 0.000 | 0.021 |
| CCR7 | 0.569 | 0.421 | 0.769 | 0.000 |  | NCR1 | 0.095 | 0.012 | 0.741 | 0.025 |
| CD19 | 0.489 | 0.287 | 0.833 | 0.009 |  | UCN2 | 0.629 | 0.416 | 0.949 | 0.027 |
| CD3D | 0.746 | 0.627 | 0.889 | 0.001 |  | GNLY | 0.733 | 0.556 | 0.967 | 0.028 |
| CD48 | 0.687 | 0.538 | 0.876 | 0.003 |  | ZAP70 | 0.578 | 0.353 | 0.944 | 0.029 |
| CD79A | 0.803 | 0.671 | 0.960 | 0.016 |  | CD244 | 0.324 | 0.118 | 0.894 | 0.029 |
| CD79B | 0.626 | 0.443 | 0.883 | 0.008 |  | IL18RAP | 0.344 | 0.123 | 0.957 | 0.041 |
| CD8A | 0.740 | 0.607 | 0.902 | 0.003 |  | XCL2 | 0.582 | 0.345 | 0.982 | 0.043 |
| CD8B | 0.770 | 0.625 | 0.947 | 0.013 |  | FASLG | 0.553 | 0.309 | 0.988 | 0.046 |
| CHIT1 | 0.591 | 0.420 | 0.832 | 0.003 |  | TNFRSF9 | 0.378 | 0.144 | 0.989 | 0.047 |
| CRP | 2.310 | 1.502 | 3.554 | 0.000 |  |  |  |  |  |  |
| CSF2 | 1.291 | 1.048 | 1.591 | 0.016 |  |  |  |  |  |  |
| CTLA4 | 0.651 | 0.481 | 0.881 | 0.005 |  |  |  |  |  |  |
| CXCL2 | 1.242 | 1.088 | 1.418 | 0.001 |  |  |  |  |  |  |
| CXCL8 | 1.290 | 1.142 | 1.457 | 0.000 |  |  |  |  |  |  |
| CXCL9 | 0.894 | 0.799 | 0.999 | 0.048 |  |  |  |  |  |  |
| CXCR3 | 0.672 | 0.514 | 0.879 | 0.004 |  |  |  |  |  |  |
| CXCR6 | 0.612 | 0.452 | 0.827 | 0.001 |  |  |  |  |  |  |
| EREG | 1.401 | 1.163 | 1.687 | 0.000 |  |  |  |  |  |  |
| FGF5 | 2.019 | 1.156 | 3.527 | 0.014 |  |  |  |  |  |  |
| GALP | 11.882 | 1.478 | 95.502 | 0.020 |  |  |  |  |  |  |
| GUCA2A | 1.263 | 1.019 | 1.565 | 0.033 |  |  |  |  |  |  |
| HCST | 0.789 | 0.645 | 0.965 | 0.021 |  |  |  |  |  |  |
| ICOS | 0.524 | 0.342 | 0.803 | 0.003 |  |  |  |  |  |  |
| IL11 | 1.238 | 1.024 | 1.498 | 0.028 |  |  |  |  |  |  |
| IL17C | 1.982 | 1.185 | 3.316 | 0.009 |  |  |  |  |  |  |
| IL22RA2 | 0.408 | 0.193 | 0.861 | 0.019 |  |  |  |  |  |  |
| IL6 | 1.187 | 1.003 | 1.404 | 0.046 |  |  |  |  |  |  |
| INSL4 | 1.396 | 1.010 | 1.930 | 0.043 |  |  |  |  |  |  |
| LBP | 1.305 | 1.065 | 1.599 | 0.010 |  |  |  |  |  |  |
| LTA | 0.536 | 0.301 | 0.955 | 0.034 |  |  |  |  |  |  |
| PAEP | 1.240 | 1.082 | 1.421 | 0.002 |  |  |  |  |  |  |
| PF4 | 1.393 | 1.012 | 1.917 | 0.042 |  |  |  |  |  |  |
| PGC | 1.297 | 1.001 | 1.681 | 0.049 |  |  |  |  |  |  |
| PTGS2 | 1.202 | 1.040 | 1.390 | 0.013 |  |  |  |  |  |  |
| PTX3 | 1.405 | 1.062 | 1.860 | 0.017 |  |  |  |  |  |  |
| REG1A | 1.217 | 1.054 | 1.406 | 0.008 |  |  |  |  |  |  |
| SERPINA3 | 1.682 | 1.191 | 2.376 | 0.003 |  |  |  |  |  |  |
| SH2D1A | 0.563 | 0.387 | 0.819 | 0.003 |  |  |  |  |  |  |
| STC1 | 1.239 | 1.080 | 1.421 | 0.002 |  |  |  |  |  |  |
| TNFRSF11B | 1.295 | 1.087 | 1.544 | 0.004 |  |  |  |  |  |  |
| TNFRSF13B | 0.281 | 0.105 | 0.756 | 0.012 |  |  |  |  |  |  |
| UCN3 | 1.622 | 1.122 | 2.345 | 0.010 |  |  |  |  |  |  |
| VCAM1 | 0.692 | 0.531 | 0.902 | 0.007 |  |  |  |  |  |  |
| XCR1 | 0.364 | 0.136 | 0.972 | 0.044 |  |  |  |  |  |  |
| ZAP70 | 0.554 | 0.406 | 0.756 | 0.000 |  |  |  |  |  |  |

## Table S5. Significant FARGs screened out by univariable cox regression analysis for APS model fitting

| **OS related gene** | | | | |  | **RFS related gene** | | | | |
| --- | --- | --- | --- | --- | --- | --- | --- | --- | --- | --- |
| **id** | **HR** | **HR.95L** | **HR.95H** | ***P*-value** |  | **id** | **HR** | **HR.95L** | **HR.95H** | ***P*-value** |
| ADAM17 | 1.423 | 1.039 | 1.950 | 0.028 |  | ADAM12 | 1.446 | 1.003 | 2.084 | 0.048 |
| ADAM9 | 1.652 | 1.201 | 2.273 | 0.002 |  | CDH11 | 1.566 | 1.107 | 2.216 | 0.011 |
| CD6 | 0.486 | 0.339 | 0.699 | 0.000 |  | CDH5 | 2.287 | 1.553 | 3.368 | 0.000 |
| CDH5 | 1.364 | 1.035 | 1.798 | 0.027 |  | COL3A1 | 1.280 | 1.030 | 1.591 | 0.026 |
| COL4A1 | 1.326 | 1.083 | 1.623 | 0.006 |  | DDR2 | 1.794 | 1.104 | 2.915 | 0.018 |
| COL4A6 | 1.249 | 1.037 | 1.504 | 0.019 |  | HRAS | 0.553 | 0.315 | 0.969 | 0.039 |
| DGCR6 | 0.410 | 0.215 | 0.783 | 0.007 |  | MFAP4 | 1.418 | 1.107 | 1.815 | 0.006 |
| FAT1 | 1.275 | 1.002 | 1.623 | 0.048 |  | MPDZ | 1.998 | 1.177 | 3.392 | 0.010 |
| HES5 | 0.643 | 0.416 | 0.992 | 0.046 |  | PCDHA11 | 4.348 | 1.183 | 15.987 | 0.027 |
| HLA-DMA | 0.851 | 0.728 | 0.995 | 0.043 |  | PCDHAC2 | 2.350 | 1.044 | 5.289 | 0.039 |
| HLA-DPB1 | 0.805 | 0.692 | 0.935 | 0.005 |  | PCDHB6 | 2.755 | 1.313 | 5.781 | 0.007 |
| HLA-DRB1 | 0.862 | 0.758 | 0.979 | 0.023 |  | PCDHB8 | 1.904 | 1.100 | 3.294 | 0.021 |
| HSPG2 | 1.324 | 1.048 | 1.672 | 0.019 |  | PCDHGB7 | 4.159 | 1.574 | 10.988 | 0.004 |
| ILK | 1.585 | 1.004 | 2.501 | 0.048 |  | PDGFD | 1.900 | 1.147 | 3.147 | 0.013 |
| ITGA2B | 0.684 | 0.478 | 0.977 | 0.037 |  | PDGFRA | 1.726 | 1.173 | 2.539 | 0.006 |
| ITGA5 | 1.510 | 1.268 | 1.797 | 0.000 |  | POSTN | 1.255 | 1.034 | 1.522 | 0.022 |
| ITGB1 | 1.517 | 1.169 | 1.969 | 0.002 |  | PTPRM | 1.514 | 1.006 | 2.279 | 0.047 |
| ITGB1.1 | 1.517 | 1.169 | 1.969 | 0.002 |  | S1PR1 | 1.855 | 1.107 | 3.109 | 0.019 |
| LAMC2 | 1.170 | 1.014 | 1.350 | 0.032 |  | SORBS1 | 1.881 | 1.046 | 3.383 | 0.035 |
| MPDZ | 1.485 | 1.066 | 2.070 | 0.020 |  | SPOCK1 | 1.356 | 1.006 | 1.829 | 0.046 |
| NECTIN3 | 1.344 | 1.029 | 1.755 | 0.030 |  | SUSD5 | 2.379 | 1.372 | 4.126 | 0.002 |
| PCDHA11 | 4.606 | 1.965 | 10.794 | 0.000 |  | VCAN | 1.374 | 1.051 | 1.797 | 0.020 |
| PCDHAC2 | 2.224 | 1.234 | 4.009 | 0.008 |  |  |  |  |  |  |
| PCDHB12 | 2.267 | 1.203 | 4.272 | 0.011 |  |  |  |  |  |  |
| PCDHB5 | 1.864 | 1.173 | 2.961 | 0.008 |  |  |  |  |  |  |
| PCDHGA10 | 1.556 | 1.056 | 2.293 | 0.025 |  |  |  |  |  |  |
| PCDHGA5 | 5.508 | 1.260 | 24.075 | 0.023 |  |  |  |  |  |  |
| PCDHGA7 | 4.978 | 1.229 | 20.165 | 0.025 |  |  |  |  |  |  |
| PCDHGB2 | 1.556 | 1.005 | 2.411 | 0.048 |  |  |  |  |  |  |
| PCDHGB3 | 17.254 | 2.300 | 129.445 | 0.006 |  |  |  |  |  |  |
| PCDHGB6 | 5.321 | 1.013 | 27.941 | 0.048 |  |  |  |  |  |  |
| PCDHGC3 | 1.354 | 1.071 | 1.712 | 0.011 |  |  |  |  |  |  |
| PCDHGC4 | 72.926 | 1.883 | 2823.912 | 0.021 |  |  |  |  |  |  |
| PIK3R2 | 0.099 | 0.021 | 0.468 | 0.004 |  |  |  |  |  |  |
| POSTN | 1.185 | 1.046 | 1.342 | 0.008 |  |  |  |  |  |  |
| PSTPIP1 | 0.556 | 0.387 | 0.799 | 0.002 |  |  |  |  |  |  |
| PTPRM | 1.368 | 1.058 | 1.769 | 0.017 |  |  |  |  |  |  |
| SPON1 | 1.211 | 1.011 | 1.449 | 0.037 |  |  |  |  |  |  |
| VCAM1 | 0.692 | 0.531 | 0.902 | 0.007 |  |  |  |  |  |  |

## Table S6. GSEA analysis of *ZBTB32* using KEGG pathways in CC

| **ID** | **setSize** | **enrichmentScore** | **NES** | ***P*-value** | **rank** |
| --- | --- | --- | --- | --- | --- |
| KEGG_OLFACTORY_TRANSDUCTION | 383 | -0.476 | -1.338 | 0.014 | 4832 |
| KEGG_HEMATOPOIETIC_CELL_LINEAGE | 85 | 0.667 | 1.723 | 0.020 | 9181 |
| KEGG_PRIMARY_IMMUNODEFICIENCY | 35 | 0.760 | 1.661 | 0.020 | 7933 |
| KEGG_MATURITY_ONSET_DIABETES_OF_THE_YOUNG | 25 | -0.764 | -1.503 | 0.022 | 8042 |
| KEGG_B_CELL_RECEPTOR_SIGNALING_PATHWAY | 75 | 0.640 | 1.583 | 0.022 | 10403 |
| KEGG_CELL_ADHESION_MOLECULES_CAMS | 131 | 0.549 | 1.454 | 0.024 | 13761 |
| KEGG_T_CELL_RECEPTOR_SIGNALING_PATHWAY | 108 | 0.672 | 1.713 | 0.024 | 10403 |
| KEGG_CYTOKINE_CYTOKINE_RECEPTOR_INTERACTION | 264 | 0.483 | 1.395 | 0.026 | 9920 |
| KEGG_ASTHMA | 28 | 0.690 | 1.489 | 0.038 | 14235 |
| KEGG_ALLOGRAFT_REJECTION | 35 | 0.712 | 1.555 | 0.041 | 12798 |
| KEGG_GLYOXYLATE_AND_DICARBOXYLATE_METABOLISM | 16 | 0.818 | 1.605 | 0.041 | 2738 |
| KEGG_GRAFT_VERSUS_HOST_DISEASE | 37 | 0.717 | 1.567 | 0.043 | 12798 |
| KEGG_LEISHMANIA_INFECTION | 69 | 0.623 | 1.496 | 0.043 | 14235 |
| KEGG_INTESTINAL_IMMUNE_NETWORK_FOR_IGA_PRODUCTION | 46 | 0.701 | 1.571 | 0.043 | 12805 |
| KEGG_SYSTEMIC_LUPUS_ERYTHEMATOSUS | 135 | 0.517 | 1.378 | 0.048 | 15087 |
| KEGG_NATURAL_KILLER_CELL_MEDIATED_CYTOTOXICITY | 132 | 0.530 | 1.401 | 0.049 | 10313 |

## Table S7. GSEA analysis of ZBTB32 using GO geneset in CC

| **ID** | **setSize** | **enrichmentScore** | **NES** | ***P*-value** | **rank** |
| --- | --- | --- | --- | --- | --- |
| GOMF_BASAL_TRANSCRIPTION_MACHINERY_BINDING | 68 | -0.699 | -1.533 | 0.018 | 294 |
| GOMF_RNA_POLYMERASE_BINDING | 72 | -0.672 | -1.489 | 0.018 | 294 |
| GOBP_REGULATION_OF_CYTOPLASMIC_TRANSLATION | 21 | -0.831 | -1.562 | 0.018 | 3112 |
| GOBP_RESPONSE_TO_PLATELET_DERIVED_GROWTH_FACTOR | 21 | -0.821 | -1.544 | 0.018 | 147 |
| GOMF_PROTEIN_DISULFIDE_ISOMERASE_ACTIVITY | 19 | -0.818 | -1.556 | 0.018 | 1107 |
| GOBP_NEGATIVE_REGULATION_OF_METALLOPEPTIDASE_ACTIVITY | 10 | -0.926 | -1.632 | 0.018 | 128 |
| GOBP_POSITIVE_REGULATION_OF_CHOLESTEROL_METABOLIC_PROCESS | 10 | -0.900 | -1.586 | 0.018 | 39 |
| GOBP_POSITIVE_REGULATION_OF_CYTOPLASMIC_TRANSLATION | 12 | -0.911 | -1.594 | 0.018 | 3112 |
| GOBP_CD4_POSITIVE_ALPHA_BETA_T_CELL_DIFFERENTIATION | 80 | 0.632 | 1.635 | 0.019 | 8631 |
| GOBP_CELLULAR_RESPONSE_TO_STEROL | 24 | -0.736 | -1.427 | 0.019 | 39 |
| GOBP_POSITIVE_REGULATION_OF_INTERLEUKIN_6_PRODUCTION | 84 | 0.577 | 1.490 | 0.019 | 16066 |
| GOBP_REGULATION_OF_ACUTE_INFLAMMATORY_RESPONSE_TO_ANTIGENIC_STIMULUS | 14 | 0.839 | 1.489 | 0.019 | 5014 |
| GOBP_REGULATION_OF_LEUKOCYTE_APOPTOTIC_PROCESS | 80 | 0.637 | 1.647 | 0.019 | 14858 |
| HP_HEPATITIS | 84 | 0.600 | 1.550 | 0.019 | 7599 |
| GOBP_CELL_ADHESION_MOLECULE_PRODUCTION | 23 | -0.763 | -1.440 | 0.019 | 498 |
| GOBP_ESTABLISHMENT_OF_LYMPHOCYTE_POLARITY | 13 | 0.844 | 1.475 | 0.019 | 6952 |
| GOBP_PROTEIN_MATURATION_BY_IRON_SULFUR_CLUSTER_TRANSFER | 16 | 0.930 | 1.675 | 0.019 | 189 |
| GOBP_REGULATION_OF_METALLOPEPTIDASE_ACTIVITY | 17 | -0.867 | -1.558 | 0.019 | 128 |
| GOBP_MYELOID_DENDRITIC_CELL_ACTIVATION | 29 | 0.754 | 1.517 | 0.020 | 9900 |
| GOBP_NEGATIVE_REGULATION_OF_ANTIGEN_RECEPTOR_MEDIATED_SIGNALING_PATHWAY | 29 | 0.743 | 1.495 | 0.020 | 10363 |
| GOBP_REGULATION_OF_INFLAMMATORY_RESPONSE_TO_ANTIGENIC_STIMULUS | 29 | 0.786 | 1.582 | 0.020 | 5634 |
| GOBP_B_CELL_ACTIVATION_INVOLVED_IN_IMMUNE_RESPONSE | 79 | 0.679 | 1.724 | 0.020 | 8434 |
| GOBP_IRON_ION_HOMEOSTASIS | 86 | 0.570 | 1.473 | 0.020 | 5180 |
| GOBP_LEUKOCYTE_HOMEOSTASIS | 79 | 0.622 | 1.579 | 0.020 | 14574 |
| GOBP_NEGATIVE_REGULATION_OF_CHOLESTEROL_EFFLUX | 15 | -0.822 | -1.459 | 0.020 | 8352 |
| GOBP_NEGATIVE_REGULATION_OF_INTERLEUKIN_12_PRODUCTION | 17 | 0.824 | 1.483 | 0.020 | 6585 |
| GOBP_NEGATIVE_REGULATION_OF_PHOSPHATIDYLINOSITOL_3_KINASE_SIGNALING | 15 | -0.832 | -1.477 | 0.020 | 57 |
| GOBP_PHAGOCYTOSIS_RECOGNITION | 87 | 0.771 | 2.019 | 0.020 | 8498 |
| GOBP_REGULATION_OF_T_CELL_MEDIATED_IMMUNITY | 76 | 0.649 | 1.638 | 0.020 | 12798 |
| GOCC_EXTRINSIC_COMPONENT_OF_MITOCHONDRIAL_INNER_MEMBRANE | 15 | -0.817 | -1.450 | 0.020 | 1698 |
| GOCC_TRANSCRIPTIONALLY_ACTIVE_CHROMATIN | 23 | 0.843 | 1.647 | 0.020 | 3079 |
| GOBP_IRON_SULFUR_CLUSTER_ASSEMBLY | 24 | 0.890 | 1.706 | 0.020 | 189 |
| GOBP_POSITIVE_REGULATION_OF_INFLAMMATORY_RESPONSE | 128 | 0.580 | 1.643 | 0.020 | 11382 |
| GOBP_POSITIVE_REGULATION_OF_INTERLEUKIN_2_PRODUCTION | 30 | 0.736 | 1.505 | 0.020 | 10403 |
| GOBP_POSITIVE_REGULATION_OF_NATURAL_KILLER_CELL_MEDIATED_CYTOTOXICITY | 26 | 0.821 | 1.561 | 0.020 | 7846 |
| GOBP_POSITIVE_REGULATION_OF_T_CELL_MIGRATION | 30 | 0.711 | 1.455 | 0.020 | 9395 |
| GOBP_REGULATION_OF_B_CELL_DIFFERENTIATION | 30 | 0.720 | 1.473 | 0.020 | 11505 |
| GOBP_REGULATION_OF_LEUKOCYTE_MEDIATED_CYTOTOXICITY | 78 | 0.716 | 1.806 | 0.020 | 10312 |
| GOBP_SOMATIC_DIVERSIFICATION_OF_IMMUNE_RECEPTORS | 75 | 0.612 | 1.539 | 0.020 | 7331 |
| GOBP_T_CELL_CHEMOTAXIS | 27 | 0.747 | 1.435 | 0.020 | 8703 |
| GOBP_TYPE_I_INTERFERON_PRODUCTION | 128 | 0.540 | 1.530 | 0.020 | 13967 |
| HP_GASTROINTESTINAL_STROMA_TUMOR | 18 | 0.819 | 1.500 | 0.020 | 2827 |
| HP_SEVERE_INFECTION | 28 | 0.732 | 1.429 | 0.020 | 11815 |
| GOBP_B_CELL_RECEPTOR_SIGNALING_PATHWAY | 120 | 0.797 | 2.219 | 0.021 | 8685 |
| GOBP_COLLAGEN_ACTIVATED_TYROSINE_KINASE_RECEPTOR_SIGNALING_PATHWAY | 12 | 0.857 | 1.454 | 0.021 | 834 |
| GOBP_GRANULOCYTE_CHEMOTAXIS | 120 | 0.586 | 1.631 | 0.021 | 12618 |
| GOBP_MEIOTIC_CELL_CYCLE_PHASE_TRANSITION | 10 | 0.842 | 1.369 | 0.021 | 2183 |
| GOBP_NEGATIVE_REGULATION_OF_IMMUNE_EFFECTOR_PROCESS | 125 | 0.566 | 1.588 | 0.021 | 8703 |
| GOBP_NEGATIVE_REGULATION_OF_LEUKOCYTE_PROLIFERATION | 88 | 0.615 | 1.596 | 0.021 | 10037 |
| GOBP_POSITIVE_REGULATION_OF_NATURAL_KILLER_CELL_MEDIATED_IMMUNITY | 31 | 0.789 | 1.606 | 0.021 | 7846 |
| GOCC_U1_SNRNP | 31 | 0.708 | 1.442 | 0.021 | 5059 |
| HP_CHRONIC_MUCOCUTANEOUS_CANDIDIASIS | 31 | 0.715 | 1.456 | 0.021 | 10831 |
| HP_MENINGITIS | 73 | 0.693 | 1.738 | 0.021 | 13110 |
| GOBP_CELLULAR_EXTRAVASATION | 69 | 0.601 | 1.476 | 0.022 | 15081 |
| GOBP_INTERFERON_GAMMA_MEDIATED_SIGNALING_PATHWAY | 89 | 0.584 | 1.515 | 0.022 | 17572 |
| GOBP_NEUTROPHIL_MIGRATION | 118 | 0.593 | 1.639 | 0.022 | 12618 |
| GOBP_POSITIVE_REGULATION_OF_MITOCHONDRIAL_MEMBRANE_POTENTIAL | 11 | 0.849 | 1.392 | 0.022 | 1434 |
| GOBP_POSITIVE_REGULATION_OF_TUMOR_NECROSIS_FACTOR_SUPERFAMILY_CYTOKINE_PRODUCTION | 89 | 0.587 | 1.522 | 0.022 | 12340 |
| GOBP_REGULATION_OF_PHAGOCYTOSIS | 92 | 0.589 | 1.533 | 0.022 | 10965 |
| GOCC_CHAPERONIN_CONTAINING_T_COMPLEX | 11 | 0.875 | 1.433 | 0.022 | 1278 |
| HP_LEUKOCYTOSIS | 118 | 0.494 | 1.364 | 0.022 | 13660 |
| HP_LYMPHOPENIA | 121 | 0.620 | 1.727 | 0.022 | 13185 |
| HP_SEPSIS | 92 | 0.532 | 1.384 | 0.022 | 13110 |
| HP_UNUSUAL_INFECTION_BY_ANATOMICAL_SITE | 92 | 0.632 | 1.644 | 0.022 | 13110 |
| GOBP_ANTIGEN_PROCESSING_AND_PRESENTATION_OF_PEPTIDE_ANTIGEN_VIA_MHC_CLASS_I | 99 | 0.533 | 1.429 | 0.022 | 19771 |
| GOBP_CHEMOKINE_PRODUCTION | 93 | 0.505 | 1.311 | 0.022 | 18240 |
| GOBP_CYTOKINE_PRODUCTION_INVOLVED_IN_IMMUNE_RESPONSE | 93 | 0.573 | 1.489 | 0.022 | 12763 |
| GOBP_NEGATIVE_REGULATION_OF_LEUKOCYTE_CELL_CELL_ADHESION | 136 | 0.578 | 1.647 | 0.022 | 13917 |
| GOBP_NEGATIVE_REGULATION_OF_RESPONSE_TO_BIOTIC_STIMULUS | 99 | 0.518 | 1.388 | 0.022 | 14135 |
| GOBP_NEUTROPHIL_CHEMOTAXIS | 99 | 0.597 | 1.599 | 0.022 | 12618 |
| GOBP_POSITIVE_REGULATION_OF_PRODUCTION_OF_MOLECULAR_MEDIATOR_OF_IMMUNE_RESPONSE | 99 | 0.573 | 1.535 | 0.022 | 12763 |
| GOBP_REGULATION_OF_ALPHA_BETA_T_CELL_ACTIVATION | 99 | 0.642 | 1.719 | 0.022 | 8703 |
| GOBP_REGULATION_OF_MONONUCLEAR_CELL_MIGRATION | 111 | 0.628 | 1.727 | 0.022 | 15081 |
| GOBP_T_CELL_DIFFERENTIATION_INVOLVED_IN_IMMUNE_RESPONSE | 72 | 0.645 | 1.597 | 0.022 | 8419 |
| GOCC_TERTIARY_GRANULE_MEMBRANE | 72 | 0.607 | 1.504 | 0.022 | 10032 |
| GOMF_CYTOKINE_BINDING | 136 | 0.523 | 1.492 | 0.022 | 14965 |
| GOMF_IMMUNOGLOBULIN_RECEPTOR_BINDING | 68 | 0.833 | 2.042 | 0.022 | 8882 |
| HP_INCREASED_CSF_LACTATE | 99 | 0.530 | 1.418 | 0.022 | 397 |
| HP_LYMPHOMA | 99 | 0.617 | 1.653 | 0.022 | 9427 |
| HP_RECURRENT_SKIN_INFECTIONS | 99 | 0.651 | 1.744 | 0.022 | 11698 |
| HP_RECURRENT_UPPER_RESPIRATORY_TRACT_INFECTIONS | 124 | 0.530 | 1.468 | 0.022 | 7933 |
| GOBP_B_CELL_DIFFERENTIATION | 137 | 0.518 | 1.464 | 0.023 | 8596 |
| GOBP_DENDRITIC_CELL_DIFFERENTIATION | 46 | 0.667 | 1.497 | 0.023 | 11161 |
| GOBP_INFLAMMATORY_RESPONSE_TO_ANTIGENIC_STIMULUS | 59 | 0.666 | 1.537 | 0.023 | 13761 |
| GOBP_INTERFERON_GAMMA_PRODUCTION | 110 | 0.659 | 1.804 | 0.023 | 13771 |
| GOBP_INTERLEUKIN_10_PRODUCTION | 61 | 0.646 | 1.504 | 0.023 | 13771 |
| GOBP_INTERLEUKIN_12_PRODUCTION | 59 | 0.701 | 1.617 | 0.023 | 7823 |
| GOBP_LEUKOCYTE_MEDIATED_CYTOTOXICITY | 110 | 0.686 | 1.877 | 0.023 | 10312 |
| GOBP_LYMPHOCYTE_APOPTOTIC_PROCESS | 71 | 0.681 | 1.669 | 0.023 | 8251 |
| GOBP_LYMPHOCYTE_COSTIMULATION | 59 | 0.710 | 1.639 | 0.023 | 7846 |
| GOBP_MEMBRANE_INVAGINATION | 132 | 0.761 | 2.143 | 0.023 | 8685 |
| GOBP_MONOCYTE_CHEMOTAXIS | 66 | 0.649 | 1.588 | 0.023 | 11559 |
| GOBP_NATURAL_KILLER_CELL_MEDIATED_IMMUNITY | 67 | 0.726 | 1.785 | 0.023 | 9736 |
| GOBP_POSITIVE_REGULATION_OF_ALPHA_BETA_T_CELL_ACTIVATION | 64 | 0.639 | 1.529 | 0.023 | 8703 |
| GOBP_POSITIVE_REGULATION_OF_INTERFERON_GAMMA_PRODUCTION | 64 | 0.723 | 1.731 | 0.023 | 11200 |
| GOBP_POSITIVE_REGULATION_OF_LEUKOCYTE_CHEMOTAXIS | 91 | 0.617 | 1.606 | 0.023 | 12164 |
| GOBP_POSITIVE_REGULATION_OF_LEUKOCYTE_MEDIATED_IMMUNITY | 123 | 0.673 | 1.858 | 0.023 | 7949 |
| GOBP_POSITIVE_REGULATION_OF_LYMPHOCYTE_DIFFERENTIATION | 100 | 0.574 | 1.544 | 0.023 | 15003 |
| GOBP_POSITIVE_REGULATION_OF_PHAGOCYTOSIS | 65 | 0.641 | 1.557 | 0.023 | 10691 |
| GOBP_REGULATION_OF_ALPHA_BETA_T_CELL_DIFFERENTIATION | 66 | 0.627 | 1.535 | 0.023 | 7879 |
| GOBP_REGULATION_OF_B_CELL_PROLIFERATION | 61 | 0.695 | 1.619 | 0.023 | 12658 |
| GOBP_REGULATION_OF_CD4_POSITIVE_ALPHA_BETA_T_CELL_ACTIVATION | 65 | 0.638 | 1.547 | 0.023 | 13771 |
| GOBP_REGULATION_OF_HUMORAL_IMMUNE_RESPONSE | 137 | 0.732 | 2.067 | 0.023 | 9680 |
| GOBP_REGULATION_OF_IMMUNOGLOBULIN_PRODUCTION | 67 | 0.639 | 1.570 | 0.023 | 8703 |
| GOBP_REGULATION_OF_LEUKOCYTE_CHEMOTAXIS | 117 | 0.610 | 1.674 | 0.023 | 12340 |
| GOBP_SOMATIC_DIVERSIFICATION_OF_IMMUNOGLOBULINS | 64 | 0.657 | 1.572 | 0.023 | 7331 |
| GOBP_T_CELL_ACTIVATION_INVOLVED_IN_IMMUNE_RESPONSE | 110 | 0.516 | 1.411 | 0.023 | 7879 |
| GOBP_T_CELL_MEDIATED_CYTOTOXICITY | 45 | 0.660 | 1.476 | 0.023 | 12798 |
| GOBP_T_CELL_MEDIATED_IMMUNITY | 100 | 0.638 | 1.715 | 0.023 | 12798 |
| GOBP_T_CELL_MIGRATION | 66 | 0.682 | 1.671 | 0.023 | 11892 |
| GOBP_T_HELPER_17_TYPE_IMMUNE_RESPONSE | 32 | 0.700 | 1.444 | 0.023 | 8251 |
| GOCC_IMMUNOGLOBULIN_COMPLEX_CIRCULATING | 66 | 0.837 | 2.050 | 0.023 | 8498 |
| GOCC_KERATIN_FILAMENT | 94 | 0.516 | 1.339 | 0.023 | 6703 |
| GOCC_SM_LIKE_PROTEIN_FAMILY_COMPLEX | 110 | 0.483 | 1.322 | 0.023 | 466 |
| GOCC_SPECIFIC_GRANULE_MEMBRANE | 90 | 0.572 | 1.475 | 0.023 | 9918 |
| GOCC_T_CELL_RECEPTOR_COMPLEX | 135 | 0.874 | 2.466 | 0.023 | 6546 |
| GOMF_METAL_CLUSTER_BINDING | 67 | 0.656 | 1.612 | 0.023 | 189 |
| HP_ABNORMAL_CIRCULATING_IGG_LEVEL | 64 | 0.670 | 1.605 | 0.023 | 13556 |
| HP_ABNORMAL_CIRCULATING_IGM_LEVEL | 60 | 0.641 | 1.500 | 0.023 | 13556 |
| HP_ECZEMA | 134 | 0.520 | 1.466 | 0.023 | 13704 |
| HP_GASTROINTESTINAL_INFLAMMATION | 100 | 0.553 | 1.487 | 0.023 | 9322 |
| HP_HEMOLYTIC_ANEMIA | 123 | 0.556 | 1.535 | 0.023 | 7412 |
| HP_HEPATOSPLENOMEGALY | 117 | 0.526 | 1.443 | 0.023 | 7970 |
| HP_INCREASED_CIRCULATING_ANTIBODY_LEVEL | 91 | 0.521 | 1.354 | 0.023 | 13274 |
| HP_NEPHRITIS | 61 | 0.662 | 1.540 | 0.023 | 9680 |
| HP_RECURRENT_LOWER_RESPIRATORY_TRACT_INFECTIONS | 110 | 0.566 | 1.549 | 0.023 | 7933 |
| HP_RECURRENT_PAROXYSMAL_HEADACHE | 32 | 0.713 | 1.471 | 0.023 | 397 |
| HP_SKIN_RASH | 115 | 0.546 | 1.504 | 0.023 | 13274 |
| HP_SYSTEMIC_LUPUS_ERYTHEMATOSUS | 32 | 0.698 | 1.439 | 0.023 | 13813 |
| HP_T_LYMPHOCYTOPENIA | 45 | 0.708 | 1.583 | 0.023 | 13185 |
| HP_VASCULITIS | 65 | 0.669 | 1.625 | 0.023 | 13185 |
| GOBP_B_CELL_PROLIFERATION | 96 | 0.565 | 1.476 | 0.023 | 7288 |
| GOBP_CELLULAR_IRON_ION_HOMEOSTASIS | 70 | 0.611 | 1.496 | 0.023 | 5180 |
| GOBP_FC_RECEPTOR_MEDIATED_STIMULATORY_SIGNALING_PATHWAY | 144 | 0.790 | 2.193 | 0.023 | 9244 |
| GOBP_GRANULOCYTE_MIGRATION | 143 | 0.590 | 1.643 | 0.023 | 12340 |
| GOBP_LYMPHOCYTE_ACTIVATION_INVOLVED_IN_IMMUNE_RESPONSE | 188 | 0.546 | 1.565 | 0.023 | 8474 |
| GOBP_LYMPHOCYTE_HOMEOSTASIS | 57 | 0.665 | 1.518 | 0.023 | 9223 |
| GOBP_LYMPHOCYTE_MIGRATION | 116 | 0.674 | 1.848 | 0.023 | 12981 |
| GOBP_MONONUCLEAR_CELL_MIGRATION | 188 | 0.634 | 1.818 | 0.023 | 13917 |
| GOBP_POSITIVE_REGULATION_OF_B_CELL_ACTIVATION | 139 | 0.770 | 2.153 | 0.023 | 9260 |
| GOBP_POSITIVE_REGULATION_OF_CHEMOTAXIS | 139 | 0.505 | 1.410 | 0.023 | 12547 |
| GOBP_POSITIVE_REGULATION_OF_LEUKOCYTE_MIGRATION | 133 | 0.591 | 1.659 | 0.023 | 14449 |
| GOBP_POSITIVE_REGULATION_OF_MITOTIC_CELL_CYCLE | 116 | 0.552 | 1.513 | 0.023 | 8401 |
| GOBP_POSITIVE_REGULATION_OF_MONONUCLEAR_CELL_MIGRATION | 63 | 0.657 | 1.559 | 0.023 | 9395 |
| GOBP_POSITIVE_T_CELL_SELECTION | 36 | 0.715 | 1.487 | 0.023 | 8631 |
| GOBP_REGULATION_OF_ANTIGEN_RECEPTOR_MEDIATED_SIGNALING_PATHWAY | 63 | 0.732 | 1.737 | 0.023 | 10889 |
| GOBP_REGULATION_OF_COMPLEMENT_ACTIVATION | 114 | 0.751 | 2.068 | 0.023 | 9680 |
| GOBP_REGULATION_OF_LYMPHOCYTE_MIGRATION | 63 | 0.663 | 1.573 | 0.023 | 14965 |
| GOBP_REGULATION_OF_NATURAL_KILLER_CELL_MEDIATED_IMMUNITY | 48 | 0.777 | 1.731 | 0.023 | 7846 |
| GOBP_REGULATION_OF_T_CELL_RECEPTOR_SIGNALING_PATHWAY | 40 | 0.710 | 1.527 | 0.023 | 10403 |
| GOBP_RESPONSE_TO_CHEMOKINE | 95 | 0.578 | 1.500 | 0.023 | 10829 |
| GOBP_RESPONSE_TO_INTERLEUKIN_12 | 50 | 0.711 | 1.556 | 0.023 | 6130 |
| GOBP_T_CELL_SELECTION | 48 | 0.698 | 1.556 | 0.023 | 10831 |
| GOMF_IMMUNE_RECEPTOR_ACTIVITY | 133 | 0.619 | 1.739 | 0.023 | 12805 |
| HP_ABNORMAL_T_CELL_SUBSET_DISTRIBUTION | 47 | 0.719 | 1.605 | 0.023 | 8786 |
| HP_ANTINUCLEAR_ANTIBODY_POSITIVITY | 37 | 0.703 | 1.450 | 0.023 | 10698 |
| HP_SKIN_ULCER | 116 | 0.498 | 1.365 | 0.023 | 13188 |
| GOBP_CD4_POSITIVE_ALPHA_BETA_T_CELL_ACTIVATION | 98 | 0.618 | 1.638 | 0.024 | 8703 |
| GOBP_CELLULAR_DEFENSE_RESPONSE | 53 | 0.707 | 1.562 | 0.024 | 10037 |
| GOBP_HUMORAL_IMMUNE_RESPONSE_MEDIATED_BY_CIRCULATING_IMMUNOGLOBULIN | 145 | 0.784 | 2.180 | 0.024 | 8600 |
| GOBP_IMMUNOGLOBULIN_PRODUCTION_INVOLVED_IN_IMMUNOGLOBULIN_MEDIATED_IMMUNE_RESPONSE | 54 | 0.654 | 1.444 | 0.024 | 9838 |
| GOBP_LYMPHOCYTE_CHEMOTAXIS | 62 | 0.682 | 1.588 | 0.024 | 10884 |
| GOBP_NEGATIVE_REGULATION_OF_CELL_CELL_ADHESION | 187 | 0.544 | 1.553 | 0.024 | 13917 |
| GOBP_NEGATIVE_REGULATION_OF_LYMPHOCYTE_ACTIVATION | 152 | 0.601 | 1.652 | 0.024 | 10334 |
| GOBP_POSITIVE_REGULATION_OF_CELL_KILLING | 62 | 0.733 | 1.707 | 0.024 | 10312 |
| GOBP_POSITIVE_REGULATION_OF_T_CELL_PROLIFERATION | 98 | 0.667 | 1.768 | 0.024 | 11071 |
| GOBP_REGULATION_OF_LYMPHOCYTE_APOPTOTIC_PROCESS | 54 | 0.701 | 1.546 | 0.024 | 8251 |
| GOBP_REGULATION_OF_LYMPHOCYTE_MEDIATED_IMMUNITY | 155 | 0.653 | 1.806 | 0.024 | 8703 |
| GOBP_REGULATION_OF_T_CELL_DIFFERENTIATION | 145 | 0.572 | 1.592 | 0.024 | 14449 |
| GOBP_REGULATION_OF_T_HELPER_CELL_DIFFERENTIATION | 38 | 0.701 | 1.473 | 0.024 | 7879 |
| GOBP_SOMATIC_RECOMBINATION_OF_IMMUNOGLOBULIN_GENE_SEGMENTS | 54 | 0.671 | 1.480 | 0.024 | 7331 |
| GOBP_TUMOR_NECROSIS_FACTOR_MEDIATED_SIGNALING_PATHWAY | 172 | 0.493 | 1.389 | 0.024 | 14375 |
| GOCC_BLOOD_MICROPARTICLE | 145 | 0.625 | 1.739 | 0.024 | 10432 |
| GOCC_SMALL_NUCLEAR_RIBONUCLEOPROTEIN_COMPLEX | 98 | 0.503 | 1.333 | 0.024 | 466 |
| GOMF_MHC_PROTEIN_BINDING | 39 | 0.796 | 1.692 | 0.024 | 7081 |
| HP_ABNORMAL_CIRCULATING_IGA_LEVEL | 62 | 0.664 | 1.546 | 0.024 | 10403 |
| HP_ABNORMAL_CONJUNCTIVA_MORPHOLOGY | 142 | 0.522 | 1.443 | 0.024 | 12798 |
| HP_ABNORMAL_T_CELL_MORPHOLOGY | 62 | 0.693 | 1.614 | 0.024 | 13185 |
| HP_ABNORMALITY_OF_BONE_MARROW_CELL_MORPHOLOGY | 172 | 0.499 | 1.405 | 0.024 | 13882 |
| HP_ABNORMALITY_OF_THE_PHARYNX | 172 | 0.479 | 1.349 | 0.024 | 8212 |
| HP_AUTOIMMUNE_ANTIBODY_POSITIVITY | 62 | 0.674 | 1.568 | 0.024 | 10698 |
| HP_AUTOIMMUNE_THROMBOCYTOPENIA | 39 | 0.702 | 1.492 | 0.024 | 7283 |
| HP_DECREASED_CIRCULATING_IGA_LEVEL | 39 | 0.699 | 1.485 | 0.024 | 12658 |
| HP_DECREASED_CIRCULATING_IGG_LEVEL | 54 | 0.688 | 1.517 | 0.024 | 13556 |
| HP_PANCYTOPENIA | 109 | 0.522 | 1.417 | 0.024 | 13660 |
| HP_RECURRENT_VIRAL_INFECTIONS | 56 | 0.690 | 1.553 | 0.024 | 7933 |
| HP_UNUSUAL_FUNGAL_INFECTION | 62 | 0.643 | 1.496 | 0.024 | 13110 |
| GOBP_ALPHA_BETA_T_CELL_ACTIVATION | 147 | 0.630 | 1.740 | 0.024 | 8703 |
| GOBP_ALPHA_BETA_T_CELL_DIFFERENTIATION | 106 | 0.621 | 1.665 | 0.024 | 8631 |
| GOBP_CELL_KILLING | 176 | 0.557 | 1.564 | 0.024 | 7949 |
| GOBP_NEGATIVE_REGULATION_OF_B_CELL_ACTIVATION | 33 | 0.724 | 1.480 | 0.024 | 10334 |
| GOBP_NEGATIVE_REGULATION_OF_IMMUNE_RESPONSE | 146 | 0.549 | 1.521 | 0.024 | 13917 |
| GOBP_POSITIVE_REGULATION_OF_HEMOPOIESIS | 153 | 0.507 | 1.384 | 0.024 | 15081 |
| GOBP_POSITIVE_REGULATION_OF_LEUKOCYTE_PROLIFERATION | 147 | 0.634 | 1.750 | 0.024 | 11685 |
| GOBP_REGULATION_OF_B_CELL_ACTIVATION | 182 | 0.755 | 2.156 | 0.024 | 9260 |
| GOBP_REGULATION_OF_CELL_KILLING | 97 | 0.712 | 1.852 | 0.024 | 10312 |
| GOBP_REGULATION_OF_LYMPHOCYTE_DIFFERENTIATION | 174 | 0.582 | 1.639 | 0.024 | 15003 |
| GOBP_REGULATION_OF_NATURAL_KILLER_CELL_ACTIVATION | 33 | 0.679 | 1.388 | 0.024 | 13553 |
| GOBP_REGULATION_OF_PRODUCTION_OF_MOLECULAR_MEDIATOR_OF_IMMUNE_RESPONSE | 146 | 0.580 | 1.607 | 0.024 | 12763 |
| GOBP_T_CELL_PROLIFERATION | 194 | 0.615 | 1.753 | 0.024 | 12946 |
| GOBP_T_CELL_RECEPTOR_SIGNALING_PATHWAY | 202 | 0.591 | 1.689 | 0.024 | 12829 |
| GOCC_IMMUNOGLOBULIN_COMPLEX | 153 | 0.845 | 2.308 | 0.024 | 7776 |
| GOMF_ANTIGEN_BINDING | 153 | 0.798 | 2.180 | 0.024 | 8498 |
| GOMF_CHEMOKINE_BINDING | 33 | 0.712 | 1.454 | 0.024 | 8420 |
| GOMF_CYTOKINE_RECEPTOR_ACTIVITY | 97 | 0.605 | 1.573 | 0.024 | 12805 |
| HP_ABNORMAL_NEUTROPHIL_COUNT | 175 | 0.555 | 1.562 | 0.024 | 8786 |
| HP_ABNORMAL_PHARYNX_MORPHOLOGY | 147 | 0.502 | 1.387 | 0.024 | 8212 |
| HP_AUTOIMMUNITY | 171 | 0.551 | 1.542 | 0.024 | 13185 |
| HP_DECREASED_CIRCULATING_ANTIBODY_LEVEL | 153 | 0.627 | 1.712 | 0.024 | 8350 |
| HP_PURPURA | 106 | 0.555 | 1.488 | 0.024 | 13882 |
| GOBP_CELLULAR_RESPONSE_TO_MOLECULE_OF_BACTERIAL_ORIGIN | 201 | 0.570 | 1.630 | 0.025 | 13761 |
| GOBP_IMMUNOGLOBULIN_PRODUCTION | 197 | 0.815 | 2.322 | 0.025 | 7406 |
| GOBP_POSITIVE_REGULATION_OF_ADAPTIVE_IMMUNE_RESPONSE | 101 | 0.608 | 1.607 | 0.025 | 12798 |
| GOBP_POSITIVE_REGULATION_OF_IMMUNE_EFFECTOR_PROCESS | 216 | 0.600 | 1.732 | 0.025 | 12798 |
| GOBP_RECEPTOR_MEDIATED_ENDOCYTOSIS | 324 | 0.618 | 1.865 | 0.025 | 9807 |
| GOBP_REGULATION_OF_LEUKOCYTE_MIGRATION | 201 | 0.580 | 1.658 | 0.025 | 15168 |
| GOBP_REGULATION_OF_T_CELL_ACTIVATION | 324 | 0.604 | 1.822 | 0.025 | 12946 |
| GOBP_REGULATION_OF_T_CELL_APOPTOTIC_PROCESS | 34 | 0.704 | 1.444 | 0.025 | 8251 |
| GOBP_REGULATORY_T_CELL_DIFFERENTIATION | 35 | 0.695 | 1.447 | 0.025 | 10107 |
| GOBP_RESPONSE_TO_INTERFERON_GAMMA | 193 | 0.552 | 1.566 | 0.025 | 14745 |
| GOCC_FICOLIN_1_RICH_GRANULE | 184 | 0.504 | 1.434 | 0.025 | 15666 |
| HP_ABNORMAL_GRANULOCYTE_COUNT | 216 | 0.574 | 1.655 | 0.025 | 13660 |
| HP_ABNORMAL_LYMPHOCYTE_MORPHOLOGY | 158 | 0.613 | 1.691 | 0.025 | 13185 |
| HP_ABNORMALITY_OF_THE_LYMPH_NODES | 197 | 0.570 | 1.624 | 0.025 | 13660 |
| HP_ARTHRITIS | 197 | 0.491 | 1.399 | 0.025 | 10939 |
| HP_DECREASED_CIRCULATING_TOTAL_IGM | 35 | 0.690 | 1.436 | 0.025 | 13556 |
| HP_IMMUNODEFICIENCY | 205 | 0.617 | 1.766 | 0.025 | 13110 |
| HP_INFLAMMATORY_ABNORMALITY_OF_THE_EYE | 195 | 0.557 | 1.582 | 0.025 | 13185 |
| HP_RECURRENT_INFECTION_OF_THE_GASTROINTESTINAL_TRACT | 34 | 0.693 | 1.422 | 0.025 | 7664 |
| HP_RED_EYE | 101 | 0.604 | 1.598 | 0.025 | 12798 |
| GOBP_COMPLEMENT_ACTIVATION | 167 | 0.759 | 2.102 | 0.026 | 9488 |
| GOBP_FC_EPSILON_RECEPTOR_SIGNALING_PATHWAY | 168 | 0.782 | 2.154 | 0.026 | 8498 |
| GOBP_NEGATIVE_REGULATION_OF_CELL_ACTIVATION | 199 | 0.584 | 1.666 | 0.026 | 13917 |
| GOBP_POSITIVE_REGULATION_OF_CELL_CELL_ADHESION | 272 | 0.573 | 1.709 | 0.026 | 12829 |
| GOBP_POSITIVE_REGULATION_OF_CELL_CYCLE_PHASE_TRANSITION | 105 | 0.599 | 1.605 | 0.026 | 11091 |
| GOBP_REGULATION_OF_ADAPTIVE_IMMUNE_RESPONSE | 167 | 0.600 | 1.660 | 0.026 | 12798 |
| GOBP_TUMOR_NECROSIS_FACTOR_SUPERFAMILY_CYTOKINE_PRODUCTION | 164 | 0.488 | 1.347 | 0.026 | 11328 |
| GOCC_SPECIFIC_GRANULE | 159 | 0.480 | 1.314 | 0.026 | 9918 |
| GOCC_TERTIARY_GRANULE | 163 | 0.526 | 1.452 | 0.026 | 10032 |
| HP_ABNORMAL_RENAL_GLOMERULUS_MORPHOLOGY | 167 | 0.485 | 1.342 | 0.026 | 9778 |
| GOBP_B_CELL_MEDIATED_IMMUNITY | 215 | 0.761 | 2.189 | 0.026 | 9838 |
| GOBP_LEUKOCYTE_APOPTOTIC_PROCESS | 102 | 0.615 | 1.618 | 0.026 | 13103 |
| GOBP_MYELOID_LEUKOCYTE_MIGRATION | 211 | 0.537 | 1.532 | 0.026 | 11892 |
| GOBP_REGULATION_OF_LEUKOCYTE_DIFFERENTIATION | 277 | 0.517 | 1.541 | 0.026 | 13771 |
| GOBP_REGULATION_OF_LEUKOCYTE_MEDIATED_IMMUNITY | 207 | 0.637 | 1.816 | 0.026 | 9049 |
| GOCC_PLASMA_MEMBRANE_SIGNALING_RECEPTOR_COMPLEX | 301 | 0.765 | 2.288 | 0.027 | 7015 |
| GOBP_CELL_RECOGNITION | 221 | 0.601 | 1.717 | 0.028 | 8645 |
| GOBP_FC_RECEPTOR_SIGNALING_PATHWAY | 238 | 0.750 | 2.153 | 0.028 | 9244 |
| GOBP_LEUKOCYTE_CHEMOTAXIS | 221 | 0.557 | 1.592 | 0.028 | 12618 |
| GOBP_POSITIVE_REGULATION_OF_LEUKOCYTE_CELL_CELL_ADHESION | 230 | 0.604 | 1.743 | 0.028 | 12829 |
| GOBP_PRODUCTION_OF_MOLECULAR_MEDIATOR_OF_IMMUNE_RESPONSE | 285 | 0.757 | 2.241 | 0.028 | 7406 |
| GOBP_T_CELL_DIFFERENTIATION | 245 | 0.594 | 1.735 | 0.028 | 10403 |
| GOCC_EXTERNAL_SIDE_OF_PLASMA_MEMBRANE | 390 | 0.622 | 1.882 | 0.028 | 8498 |
| HP_LEUKOPENIA | 220 | 0.569 | 1.623 | 0.028 | 13660 |
| GOMF_OLFACTORY_RECEPTOR_ACTIVITY | 417 | -0.480 | -1.349 | 0.028 | 3098 |
| GOBP_ANTIGEN_PROCESSING_AND_PRESENTATION | 232 | 0.493 | 1.417 | 0.029 | 14546 |
| GOBP_ANTIGEN_RECEPTOR_MEDIATED_SIGNALING_PATHWAY | 307 | 0.701 | 2.089 | 0.029 | 10403 |
| GOBP_B_CELL_ACTIVATION | 314 | 0.682 | 2.025 | 0.029 | 8685 |
| GOBP_CELL_CHEMOTAXIS | 298 | 0.518 | 1.533 | 0.029 | 12618 |
| GOBP_CELLULAR_RESPONSE_TO_BIOTIC_STIMULUS | 225 | 0.564 | 1.602 | 0.029 | 13761 |
| GOBP_LEUKOCYTE_PROLIFERATION | 311 | 0.566 | 1.681 | 0.029 | 12680 |
| GOBP_NEGATIVE_REGULATION_OF_IMMUNE_SYSTEM_PROCESS | 394 | 0.546 | 1.643 | 0.029 | 15372 |
| GOBP_POSITIVE_REGULATION_OF_CELL_ACTIVATION | 396 | 0.668 | 2.015 | 0.029 | 9260 |
| GOBP_REGULATION_OF_LEUKOCYTE_PROLIFERATION | 240 | 0.620 | 1.773 | 0.029 | 12798 |
| GOBP_RESPONSE_TO_TUMOR_NECROSIS_FACTOR | 314 | 0.499 | 1.481 | 0.029 | 13038 |
| HP_ABNORMAL_LYMPHOCYTE_PHYSIOLOGY | 247 | 0.561 | 1.633 | 0.029 | 12798 |
| HP_ABNORMAL_MYELOID_LEUKOCYTE_MORPHOLOGY | 314 | 0.492 | 1.460 | 0.029 | 13660 |
| HP_ABNORMALITY_OF_HUMORAL_IMMUNITY | 250 | 0.572 | 1.658 | 0.029 | 12931 |
| HP_ABNORMALITY_OF_NEUTROPHILS | 250 | 0.470 | 1.362 | 0.029 | 13660 |
| HP_HEMATOLOGICAL_NEOPLASM | 241 | 0.516 | 1.485 | 0.029 | 13185 |
| GOBP_SENSORY_PERCEPTION_OF_SMELL | 441 | -0.479 | -1.358 | 0.029 | 4832 |
| GOBP_HUMORAL_IMMUNE_RESPONSE | 369 | 0.549 | 1.634 | 0.029 | 8600 |
| GOBP_PHAGOCYTOSIS | 368 | 0.688 | 2.045 | 0.029 | 9680 |
| GOMF_CYTOKINE_ACTIVITY | 233 | 0.446 | 1.270 | 0.029 | 10829 |
| GOBP_ADAPTIVE_IMMUNE_RESPONSE_BASED_ON_SOMATIC_RECOMBINATION_OF_IMMUNE_RECEPTORS_BUILT_FROM_IMMUNOGLOBULIN_SUPERFAMILY_DOMAINS | 351 | 0.723 | 2.161 | 0.030 | 10341 |
| GOBP_POSITIVE_REGULATION_OF_DEFENSE_RESPONSE | 352 | 0.493 | 1.474 | 0.030 | 11455 |
| GOBP_REGULATION_OF_CELL_CELL_ADHESION | 432 | 0.549 | 1.649 | 0.030 | 12829 |
| GOBP_LYMPHOCYTE_MEDIATED_IMMUNITY | 345 | 0.731 | 2.186 | 0.031 | 9488 |
| GOBP_MONONUCLEAR_CELL_DIFFERENTIATION | 409 | 0.554 | 1.664 | 0.031 | 10517 |
| GOBP_POSITIVE_REGULATION_OF_CYTOKINE_PRODUCTION | 431 | 0.562 | 1.685 | 0.031 | 12763 |
| GOBP_POSITIVE_REGULATION_OF_RESPONSE_TO_BIOTIC_STIMULUS | 253 | 0.498 | 1.437 | 0.031 | 10037 |
| GOBP_REGULATION_OF_HEMOPOIESIS | 411 | 0.493 | 1.482 | 0.031 | 15081 |
| GOBP_REGULATION_OF_IMMUNE_EFFECTOR_PROCESS | 461 | 0.658 | 1.988 | 0.031 | 10408 |
| GOBP_REGULATION_OF_RESPONSE_TO_BIOTIC_STIMULUS | 414 | 0.496 | 1.486 | 0.031 | 13361 |
| GOBP_RESPONSE_TO_MOLECULE_OF_BACTERIAL_ORIGIN | 337 | 0.516 | 1.541 | 0.031 | 13771 |
| GOBP_LEUKOCYTE_CELL_CELL_ADHESION | 360 | 0.597 | 1.758 | 0.032 | 12981 |
| GOBP_POSITIVE_REGULATION_OF_CELL_ADHESION | 420 | 0.496 | 1.482 | 0.032 | 15081 |
| GOBP_REGULATION_OF_LYMPHOCYTE_ACTIVATION | 494 | 0.664 | 2.023 | 0.032 | 10499 |
| GOBP_T_CELL_ACTIVATION | 471 | 0.560 | 1.679 | 0.032 | 10499 |
| HP_ABNORMAL_LEUKOCYTE_COUNT | 356 | 0.530 | 1.577 | 0.032 | 13660 |
| GOBP_IMMUNE_RESPONSE_REGULATING_SIGNALING_PATHWAY | 500 | 0.665 | 2.030 | 0.033 | 10403 |
| GOBP_LEUKOCYTE_MIGRATION | 500 | 0.624 | 1.904 | 0.033 | 11261 |
| GOBP_POSITIVE_REGULATION_OF_RESPONSE_TO_EXTERNAL_STIMULUS | 500 | 0.495 | 1.512 | 0.033 | 12837 |
| GOMF_RNA_POLYMERASE_CORE_ENZYME_BINDING | 56 | -0.699 | -1.617 | 0.033 | 294 |
| HP_ABNORMAL_WAIST_TO_HIP_RATIO | 37 | -0.668 | -1.438 | 0.034 | 11689 |
| GOBP_REGULATION_OF_CHOLESTEROL_EFFLUX | 41 | -0.704 | -1.540 | 0.034 | 8352 |
| GOBP_REGULATION_OF_VASCULAR_ASSOCIATED_SMOOTH_MUSCLE_CELL_PROLIFERATION | 72 | -0.651 | -1.443 | 0.035 | 454 |
| GOBP_CELLULAR_RESPONSE_TO_CHOLESTEROL | 19 | -0.778 | -1.479 | 0.036 | 39 |
| GOBP_REGULATION_OF_VASCULAR_WOUND_HEALING | 11 | -0.852 | -1.508 | 0.036 | 147 |
| GOBP_VASCULAR_WOUND_HEALING | 19 | -0.777 | -1.477 | 0.036 | 147 |
| GOBP_ANTIGEN_PROCESSING_AND_PRESENTATION_OF_EXOGENOUS_PEPTIDE_ANTIGEN_VIA_MHC_CLASS_I | 80 | 0.562 | 1.454 | 0.038 | 13214 |
| GOBP_PRODUCTION_OF_MOLECULAR_MEDIATOR_INVOLVED_IN_INFLAMMATORY_RESPONSE | 81 | 0.570 | 1.468 | 0.038 | 7680 |
| GOBP_IMMUNOLOGICAL_SYNAPSE_FORMATION | 13 | 0.832 | 1.455 | 0.038 | 6952 |
| GOBP_NEGATIVE_REGULATION_OF_STEROL_TRANSPORT | 23 | -0.701 | -1.324 | 0.038 | 8352 |
| GOBP_DENDRITIC_CELL_MIGRATION | 29 | 0.702 | 1.413 | 0.039 | 11568 |
| GOBP_REGULATION_OF_DEFENSE_RESPONSE_TO_VIRUS_BY_VIRUS | 29 | 0.710 | 1.429 | 0.039 | 9575 |
| HP_AUTOIMMUNE_HEMOLYTIC_ANEMIA | 29 | 0.697 | 1.402 | 0.039 | 13185 |
| HP_INTESTINAL_PSEUDO_OBSTRUCTION | 29 | 0.703 | 1.415 | 0.039 | 397 |
| GOBP_NEGATIVE_REGULATION_OF_B_CELL_PROLIFERATION | 17 | 0.786 | 1.414 | 0.040 | 10037 |
| GOBP_PRE_MIRNA_PROCESSING | 15 | -0.794 | -1.410 | 0.040 | 1340 |
| GOBP_REGULATION_OF_RESPONSE_TO_TUMOR_CELL | 17 | 0.783 | 1.409 | 0.040 | 7601 |
| GOBP_T_HELPER_CELL_LINEAGE_COMMITMENT | 17 | 0.770 | 1.386 | 0.040 | 7879 |
| GOBP_REGULATION_OF_MYOBLAST_PROLIFERATION | 18 | 0.780 | 1.429 | 0.041 | 499 |
| GOBP_TOLERANCE_INDUCTION | 28 | 0.729 | 1.424 | 0.041 | 6908 |
| GOCC_CHAPERONE_COMPLEX | 28 | 0.706 | 1.379 | 0.041 | 1278 |
| GOMF_C_C_CHEMOKINE_BINDING | 24 | 0.760 | 1.457 | 0.041 | 8420 |
| HP_ABNORMAL_CELL_PROLIFERATION | 27 | 0.717 | 1.377 | 0.041 | 10313 |
| GOBP_POSITIVE_REGULATION_OF_LYMPHOCYTE_CHEMOTAXIS | 20 | 0.745 | 1.405 | 0.042 | 8703 |
| GOBP_REGULATION_OF_B_CELL_RECEPTOR_SIGNALING_PATHWAY | 25 | 0.762 | 1.426 | 0.042 | 10889 |
| GOCC_TRIGLYCERIDE_RICH_PLASMA_LIPOPROTEIN_PARTICLE | 20 | 0.739 | 1.393 | 0.042 | 11644 |
| GOBP_DETECTION_OF_CHEMICAL_STIMULUS | 495 | -0.460 | -1.320 | 0.042 | 4832 |
| GOBP_MATURE_B_CELL_DIFFERENTIATION | 31 | 0.685 | 1.395 | 0.043 | 8434 |
| GOMF_PEPTIDE_ANTIGEN_BINDING | 31 | 0.690 | 1.405 | 0.043 | 14802 |
| HP_POSTERIOR_PHARYNGEAL_CLEFT | 12 | 0.837 | 1.420 | 0.043 | 2827 |
| GOBP_MYOBLAST_PROLIFERATION | 22 | 0.769 | 1.452 | 0.043 | 499 |
| GOBP_POSITIVE_REGULATION_OF_ANTIGEN_RECEPTOR_MEDIATED_SIGNALING_PATHWAY | 21 | 0.769 | 1.449 | 0.043 | 6794 |
| GOBP_REGULATION_OF_DEFENSE_RESPONSE_TO_VIRUS | 69 | 0.595 | 1.462 | 0.043 | 14216 |
| GOMF_SIALIC_ACID_BINDING | 22 | 0.775 | 1.464 | 0.043 | 7393 |
| HP_AGAMMAGLOBULINEMIA | 19 | 0.758 | 1.387 | 0.043 | 7933 |
| HP_DECREASED_LDL_CHOLESTEROL_CONCENTRATION | 11 | 0.846 | 1.386 | 0.043 | 4267 |
| HP_DISTAL_PERIPHERAL_SENSORY_NEUROPATHY | 19 | 0.746 | 1.364 | 0.043 | 397 |
| HP_ERYTHEMA | 118 | 0.466 | 1.289 | 0.043 | 13621 |
| GOBP_POSITIVE_REGULATION_OF_G1_S_TRANSITION_OF_MITOTIC_CELL_CYCLE | 43 | 0.674 | 1.516 | 0.044 | 11963 |
| GOBP_REGULATION_OF_T_CELL_MIGRATION | 43 | 0.671 | 1.509 | 0.044 | 9395 |
| GOBP_INTERLEUKIN_1_PRODUCTION | 123 | 0.482 | 1.331 | 0.045 | 11030 |
| GOBP_INTERLEUKIN_2_PRODUCTION | 61 | 0.638 | 1.486 | 0.045 | 10403 |
| GOBP_NEGATIVE_REGULATION_OF_T_CELL_PROLIFERATION | 66 | 0.580 | 1.421 | 0.045 | 8703 |
| GOBP_POSITIVE_REGULATION_OF_B_CELL_PROLIFERATION | 40 | 0.658 | 1.415 | 0.047 | 12658 |
| GOBP_POSITIVE_REGULATION_OF_CD4_POSITIVE_ALPHA_BETA_T_CELL_ACTIVATION | 37 | 0.661 | 1.364 | 0.047 | 14235 |
| GOBP_POSITIVE_REGULATION_OF_INTERLEUKIN_1_PRODUCTION | 63 | 0.624 | 1.481 | 0.047 | 11637 |
| GOBP_POSITIVE_REGULATION_OF_INTERLEUKIN_12_PRODUCTION | 40 | 0.672 | 1.447 | 0.047 | 13771 |
| GOBP_POSITIVE_REGULATION_OF_LYMPHOCYTE_MIGRATION | 37 | 0.680 | 1.402 | 0.047 | 13917 |
| GOBP_REGULATION_OF_CD4_POSITIVE_ALPHA_BETA_T_CELL_DIFFERENTIATION | 50 | 0.664 | 1.453 | 0.047 | 7879 |
| GOBP_REGULATION_OF_T_CELL_MEDIATED_CYTOTOXICITY | 37 | 0.670 | 1.383 | 0.047 | 12798 |
| GOBP_T_CELL_APOPTOTIC_PROCESS | 49 | 0.672 | 1.484 | 0.047 | 8251 |
| GOBP_T_CELL_HOMEOSTASIS | 36 | 0.667 | 1.389 | 0.047 | 8474 |
| GOCC_DNA_PACKAGING_COMPLEX | 113 | 0.474 | 1.297 | 0.047 | 17869 |
| HP_ABNORMAL_METABOLIC_BRAIN_IMAGING_BY_MRS | 37 | 0.675 | 1.392 | 0.047 | 397 |
| HP_STOMATITIS | 58 | 0.624 | 1.443 | 0.047 | 13967 |
| GOBP_NEGATIVE_REGULATION_OF_PRODUCTION_OF_MOLECULAR_MEDIATOR_OF_IMMUNE_RESPONSE | 39 | 0.647 | 1.375 | 0.048 | 8703 |
| GOBP_POSITIVE_REGULATION_OF_B_CELL_MEDIATED_IMMUNITY | 38 | 0.666 | 1.399 | 0.048 | 8703 |
| GOBP_POSITIVE_REGULATION_OF_CELL_CYCLE_G1_S_PHASE_TRANSITION | 54 | 0.639 | 1.409 | 0.048 | 11963 |
| GOBP_POSITIVE_REGULATION_OF_INTERLEUKIN_10_PRODUCTION | 38 | 0.660 | 1.386 | 0.048 | 8703 |
| HP_ABNORMAL_B_CELL_MORPHOLOGY | 38 | 0.684 | 1.436 | 0.048 | 13185 |
| HP_ABNORMAL_EOSINOPHIL_MORPHOLOGY | 56 | 0.644 | 1.448 | 0.048 | 13473 |
| HP_ABNORMALITY_OF_T_CELL_PHYSIOLOGY | 53 | 0.653 | 1.441 | 0.048 | 7283 |
| HP_INFLAMMATION_OF_THE_LARGE_INTESTINE | 62 | 0.628 | 1.461 | 0.048 | 9322 |
| HP_ORAL_ULCER | 39 | 0.690 | 1.468 | 0.048 | 7107 |
| GOBP_INTERLEUKIN_1_BETA_PRODUCTION | 106 | 0.478 | 1.283 | 0.049 | 11637 |
| GOBP_MYELOID_LEUKOCYTE_DIFFERENTIATION | 203 | 0.424 | 1.217 | 0.049 | 13661 |
| GOBP_PATTERN_RECOGNITION_RECEPTOR_SIGNALING_PATHWAY | 203 | 0.441 | 1.266 | 0.049 | 14110 |
| GOCC_INTERMEDIATE_FILAMENT | 203 | 0.420 | 1.207 | 0.049 | 6703 |
| HP_NEOPLASM_OF_THE_SKIN | 182 | 0.433 | 1.236 | 0.049 | 19247 |

## Table S8. Correlation analysis of four key genes and immune checkpoints

| **Gene** | **Checkpoint** | **Alias** | **Rs(Spearman)** | ***P*value** |
| --- | --- | --- | --- | --- |
| ZBTB32 | A2AR |  | 0.54 | 9.20E-25 |
| ZBTB32 | CD122 |  | 0.48 | 4.20E-19 |
| ZBTB32 | CD134 | OX40 | 0.57 | 3.20E-27 |
| ZBTB32 | CD137 |  | 0.52 | 9.50E-23 |
| ZBTB32 | CD152 | CTLA-4 | 0.59 | 4.00E-30 |
| ZBTB32 | CD159A | KLRC1 | 0.24 | 2.60E-05 |
| ZBTB32 | CD172A | SIRPA | 0.24 | 2.80E-05 |
| ZBTB32 | CD2 |  | 0.57 | 3.00E-27 |
| ZBTB32 | CD200R1 |  | 0.42 | 2.20E-14 |
| ZBTB32 | CD223 | LAG3 | 0.57 | 7.40E-28 |
| ZBTB32 | CD244 |  | 0.41 | 3.70E-14 |
| ZBTB32 | CD27 |  | 0.62 | 4.00E-34 |
| ZBTB32 | CD272 | BTLA | 0.52 | 3.90E-22 |
| ZBTB32 | CD278 | ICOS | 0.64 | 1.20E-36 |
| ZBTB32 | CD279 | PD-1 | 0.58 | 2.20E-29 |
| ZBTB32 | CD28 |  | 0.58 | 2.60E-29 |
| ZBTB32 | CD357 | GITR | 0.17 | 2.70E-03 |
| ZBTB32 | CD366 | TIM-3 | 0.53 | 1.20E-23 |
| ZBTB32 | CD40 |  | 0.31 | 4.60E-08 |
| ZBTB32 | CD80 |  | 0.54 | 3.20E-24 |
| ZBTB32 | CD94 | KLRD1 | 0.44 | 4.00E-16 |
| ZBTB32 | TIGIT |  | 0.56 | 3.40E-26 |
| ZBTB32 | IDO |  | 0.37 | 1.50E-11 |
| PALB2 | A2AR |  | -0.096 | 0.095 |
| PALB2 | CD122 |  | 0.097 | 0.09 |
| PALB2 | CD134 | OX40 | -0.077 | 0.18 |
| PALB2 | CD137 |  | 0.086 | 0.13 |
| PALB2 | CD152 | CTLA-4 | 0.11 | 0.053 |
| PALB2 | CD159A | KLRC1 | 0.13 | 0.027 |
| PALB2 | CD172A | SIRPA | -0.066 | 0.25 |
| PALB2 | CD2 |  | 0.13 | 0.024 |
| PALB2 | CD200R1 |  | 0.21 | 0.00026 |
| PALB2 | CD223 | LAG3 | 0.089 | 0.12 |
| PALB2 | CD244 |  | 0.099 | 0.082 |
| PALB2 | CD27 |  | 0.049 | 0.4 |
| PALB2 | CD272 | BTLA | 0.13 | 0.021 |
| PALB2 | CD278 | ICOS | 0.082 | 0.15 |
| PALB2 | CD279 | PD-1 | 0.06 | 0.29 |
| PALB2 | CD28 |  | 0.028 | 0.63 |
| PALB2 | CD357 | GITR | 0.24 | 3.10E-05 |
| PALB2 | CD366 | TIM-3 | 0.13 | 0.023 |
| PALB2 | CD40 |  | 0.16 | 0.0044 |
| PALB2 | CD80 |  | 0.11 | 0.06 |
| PALB2 | CD94 | KLRD1 | 0.12 | 0.041 |
| PALB2 | TIGIT |  | 0.15 | 0.0095 |
| PALB2 | IDO |  | 0.17 | 0.0029 |
| BRCA2 | A2AR |  | 0.085 | 0.14 |
| BRCA2 | CD122 |  | 0.23 | 5.30E-05 |
| BRCA2 | CD134 | OX40 | -0.1 | 0.078 |
| BRCA2 | CD137 |  | 0.12 | 0.039 |
| BRCA2 | CD152 | CTLA-4 | -0.066 | 0.25 |
| BRCA2 | CD159A | KLRC1 | 0.013 | 0.81 |
| BRCA2 | CD172A | SIRPA | 0.018 | 0.75 |
| BRCA2 | CD2 |  | -0.013 | 0.82 |
| BRCA2 | CD200R1 |  | 0.19 | 0.00075 |
| BRCA2 | CD223 | LAG3 | -0.13 | 0.02 |
| BRCA2 | CD244 |  | 0.057 | 0.32 |
| BRCA2 | CD27 |  | -0.079 | 0.17 |
| BRCA2 | CD272 | BTLA | -0.045 | 0.43 |
| BRCA2 | CD278 | ICOS | 0.015 | 0.79 |
| BRCA2 | CD279 | PD-1 | -0.02 | 0.73 |
| BRCA2 | CD28 |  | 0.099 | 0.083 |
| BRCA2 | CD357 | GITR | -0.015 | 0.79 |
| BRCA2 | CD366 | TIM-3 | -0.039 | 0.5 |
| BRCA2 | CD40 |  | 0.028 | 0.63 |
| BRCA2 | CD80 |  | -0.0086 | 0.88 |
| BRCA2 | CD94 | KLRD1 | 0.025 | 0.67 |
| BRCA2 | TIGIT |  | 0.036 | 0.53 |
| BRCA2 | IDO |  | 0.12 | 0.041 |
| CENPS | A2AR |  | 0.035 | 0.54 |
| CENPS | CD122 |  | -0.035 | 0.54 |
| CENPS | CD134 | OX40 | -0.073 | 0.2 |
| CENPS | CD137 |  | -0.01 | 0.86 |
| CENPS | CD152 | CTLA-4 | -0.096 | 0.094 |
| CENPS | CD159A | KLRC1 | -0.061 | 0.29 |
| CENPS | CD172A | SIRPA | -0.19 | 0.00062 |
| CENPS | CD2 |  | -0.051 | 0.37 |
| CENPS | CD200R1 |  | 0.036 | 0.53 |
| CENPS | CD223 | LAG3 | -0.15 | 0.011 |
| CENPS | CD244 |  | -0.11 | 0.06 |
| CENPS | CD27 |  | -0.063 | 0.27 |
| CENPS | CD272 | BTLA | -0.027 | 0.64 |
| CENPS | CD278 | ICOS | -0.082 | 0.15 |
| CENPS | CD279 | PD-1 | -0.085 | 0.14 |
| CENPS | CD28 |  | -0.035 | 0.54 |
| CENPS | CD357 | GITR | 0.14 | 0.011 |
| CENPS | CD366 | TIM-3 | -0.1 | 0.082 |
| CENPS | CD40 |  | -0.033 | 0.57 |
| CENPS | CD80 |  | -0.038 | 0.51 |
| CENPS | CD94 | KLRD1 | -0.074 | 0.2 |
| CENPS | TIGIT |  | -0.04 | 0.49 |
| CENPS | IDO |  | 0.048 | 0.41 |
