# Supplementary material for: Fanconi Anemia Pathway Genes Advance Cervical Cancer via Immune Regulation and Cell Adhesion
Source: Front Cell Dev Biol. 2021 Nov 15;9:734794. doi: 10.3389/fcell.2021.734794 (PMC8634638; doi:10.3389/fcell.2021.734794)
Supplement: Supplementary file 3 [file Table3.DOCX]

## Supplement Materials for reviews:

https://www.jianguoyun.com/p/Da6KHlMQ8a7yCRi3tZEE

(00 Peer Review Only.zip — — This file is a worksheet organized in sequence according to the format of our pictures, which contains the uploaded public data)

https://www.jianguoyun.com/p/DZNwVzEQ8a7yCRjQipEE

(01 Transwell.zip —— The original format picture of Transwell experiment inserted in this manuscript (Figure S13E and S13F))
https://www.jianguoyun.com/p/DUQplg4Q8a7yCRjRipEE

(02 IHC_BRCA2.zip —— The original results of IHC inserted in this manuscript include cancer and normal tissue sections (Figure S12C))
https://www.jianguoyun.com/p/Db2PnhoQ8a7yCRjWipEE

(02 IHC_PALB2.zip —— The original results of IHC inserted in this manuscript include cancer and normal tissue sections (Figure S12C))
https://www.jianguoyun.com/p/DWvH9F8Q8a7yCRjPipEE

(02 IHC_ZBTB32.zip —— The original results of IHC inserted in this manuscript include cancer and normal tissue sections (Figure S12C))
